# Accuracy Meets Interpretability for Computational Spectroscopy by Means of Hybrid and Double-Hybrid Functionals

**DOI:** 10.3389/fchem.2020.584203

**Published:** 2020-10-23

**Authors:** Vincenzo Barone, Giorgia Ceselin, Marco Fusè, Nicola Tasinato

**Affiliations:** SMART Laboratory, Scuola Normale Superiore di Pisa, Pisa, Italy

**Keywords:** quantum chemistry, density functional theory, rotational spectroscopy, vibrational spectroscopy, benchmark, atmospheric molecules, astrochemical molecules

## Abstract

Accuracy and interpretability are often seen as the devil and holy grail in computational spectroscopy and their reconciliation remains a primary research goal. In the last few decades, density functional theory has revolutionized the situation, paving the way to reliable yet effective models for medium size molecules, which could also be profitably used by non-specialists. In this contribution we will compare the results of some widely used hybrid and double hybrid functionals with the aim of defining the most suitable recipe for all the spectroscopic parameters of interest in rotational and vibrational spectroscopy, going beyond the rigid rotor/harmonic oscillator model. We will show that last-generation hybrid and double hybrid functionals in conjunction with partially augmented double- and triple-zeta basis sets can offer, in the framework of second order vibrational perturbation theory, a general, robust, and user-friendly tool with unprecedented accuracy for medium-size semi-rigid molecules.

## 1. Introduction

Spectroscopic techniques are unique tools to non-invasively probe the properties of complex molecular systems in a variety of environments and conditions. In fact, the increasing sophistication of well-established techniques like nuclear magnetic and electron paramagnetic resonance (NMR and EPR), microwave (MW), infrared (IR), Raman, visible (Vis), ultra-violet (UV), or fluorescence and the parallel blooming of new ones, e.g., vibrational, electronic, and magnetic circular dichroism (VCD, ECD, and MCD), Raman optical activity (ROA), circularly polarized luminescence (CPL), multi-photon and time-resolved methods have a huge impact in several fields of science and technology (He et al., 2007; Barone, 2012; Berova et al., 2012; Tasinato et al., 2015; Sugiki et al., 2017; Lane, 2018). In addition to being widely used to infer information about molecular structure and dynamics in both gas and condensed phases (Sugiki et al., 2017; Puzzarini and Barone, 2018), spectroscopy allows for the unequivocal identification of chemical species in hostile environments, e.g., the interstellar space (Baiano et al., 2020), or in samples of unknown composition (He et al., 2007; Lindon et al., 2017; Lane, 2018) and plays a pivotal role in the study of photochemical mechanisms in biological systems and in the development of new technological devices, including photovoltaic cells, optoelectronic devices, eco-sustainable solutions, UV-resistant materials, dyes, and fluorescent probes (Berova et al., 2012; Drummen, 2012; Lindon et al., 2017; Lane, 2018). Unfortunately, interpretation of experimental data is often a difficult task: the observed spectroscopic behavior results from the subtle interplay of stereo-electronic, dynamic and environmental effects, whose specific roles are difficult to disentangle. Furthermore, although conveying additional information, spectral congestion makes quantitative interpretation of experimental data even more difficult. To disentangle these complex signatures and disclose the underlying molecular properties, detailed molecular simulations are crucial (Barone, 2012). The last decade has witnessed an increasing interaction between experiment, theory, and simulation in the field of molecular spectroscopy and in all related applications. These have revealed the need of computational tools and theoretical methods not only to interpret the spectra, but also to design new experiments that would be impossible or very expensive to perform in a blind way. The widespread use of computational techniques in many areas of science and by an ever increasing number of non-experienced users (e.g., experimental chemists) has prompted the development, in our group, of the Virtual Multifrequency Spectrometer (VMS, http://dreamslab.sns.it/vms/) (Barone, 2016). VMS features two interconnected tools: VMS-Draw (Licari et al., 2015) and VMS-Comp (Barone et al., 2012), the former providing a user-friendly, graphical user interface to pilot the latter, which is in charge of computationally intensive tasks. VMS-Comp includes a wide set of algorithms and calculation options and it allows the user to predict with remarkable accuracy many types of spectroscopic data for a vast range of molecular systems and environments (Zerbetto et al., 2013; Licari et al., 2017; Presti et al., 2017). Further developments are, however, needed to deal with new and more sophisticated experimental techniques (Quack and Merkt, 2011; Lane, 2018). For small semi-rigid molecules, the accuracy of state-of-the-art quantum mechanical (QM) methods often rivals that of experimental techniques, but extension to large flexible systems (not to mention condensed phases) faces a number of difficulties (even within the Born-Oppenheimer approximation) ranging from the very unfavorable scaling of those methods with the number of active electrons to the proper description of flat potential energy surfaces (PESs) with stationary points that are ill defined (Puzzarini et al., 2019a).

**Figure 1:**
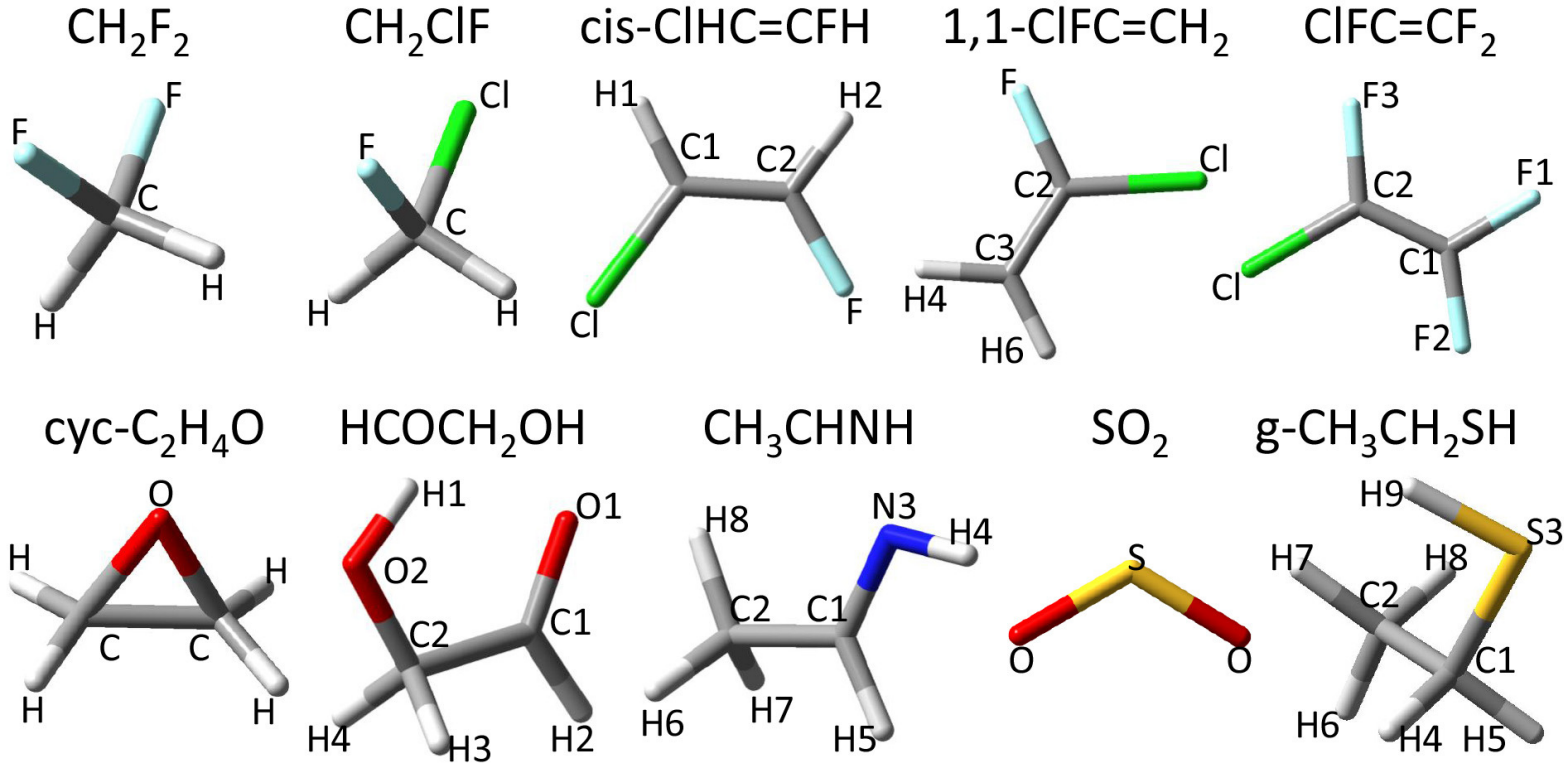
Molecules in the benchmark set.

A possible route to obtain accurate results, even for relatively large molecular systems (a few dozens of atoms), is provided by hybrid QM/QM′ models, which combine accurate quantum-mechanical (QM) calculations of “primary” properties (e.g., molecular structures or harmonic force fields) with cheaper yet reliable electronic structure approaches (QM′) for “secondary” properties (e.g., vibrational corrections or anharmonic effects). At the same time, computation of spectroscopic parameters often requires purposely tailored basis sets, whose selection must be based on extensive benchmarks. Finally, the customary rigid rotor (RR) / harmonic oscillator (HO) approximation is not sufficient for quantitative work and more advanced models must be employed. In the last few years, the second-order vibrational perturbation theory (VPT2) (Mills, 1972; Papoušek and Aliev, 1982; Aliev and Watson, 1985) has been exploited with considerable success for semi-rigid molecules of increasing dimensions. However, the identification of resonances in VPT2 treatments remains a daunting task due to the arbitrariness of their definition and their indirect influence on the energy and intensities. This leads to two distinct issues: finding the true resonances and then correcting them appropriately. The resonance conditions are strongly related to the quality of the electronic structure calculation, but they also depend on the coupling between the potentially resonant states. The most obvious solution is to combine perturbative and variational models, with the generalized (G)VPT2 model offering a very good accuracy-to-cost ratio not only for energies, but also for transition moments (Bloino et al., 2012). In recent years, we have implemented analytical second derivatives for double-hybrid functionals (Biczysko et al., 2010), general equations for Abelian and non-Abelian symmetry groups (Piccardo et al., 2015a), and full treatment of intensities for all conventional (IR, Raman) and chiral (VCD, ROA) vibrational spectroscopies up to three-quanta excitations (Bloino et al., 2015). These features compose, together with the particular care devoted to robustness, ease of use, and computational efficiency, the mandatory background for systematic evaluations of all the spectroscopic parameters beyond the rigid-rotor/harmonic-oscillator level for medium- to large-size semi-rigid molecules. Next, together with further developments for the treatment of flexible molecules (Puzzarini et al., 2019b), the selection of the most effective electronic structure models remains a central issue of computational spectroscopy. Here, the ongoing developments of methods rooted in the density functional theory (DFT) come into play. For microwave and vibrational spectroscopic applications, global hybrid density functionals (DFs), such as B3LYP (Lee et al., 1988; Becke, 1993) coupled to a polarized double-zeta basis set supplemented by a set of *sp* diffuse functions (hereafter B3) can deliver the accuracy required for the interpretation of the vibrational characteristics of medium and large molecules beyond the harmonic approximation for both transition frequencies and intensities. An increased accuracy, at the price of a more demanding computational loading, is brought by the double-hybrid B2PLYP functional (Grimme, 2006) in conjunction with partially augmented triple-ζ basis sets (hereafter B2). In fact, it has been shown that B2 calculations of vibrational frequencies and intensities can reach an average accuracy often within 10 cm^−1^ and a few km mol^−1^, respectively (Biczysko et al., 2010; Puzzarini et al., 2019a; Boussessi et al., 2020a), thus performing equally to, or even better than, the CCSD(T)/cc-pVTZ approach. Concerning the prediction of rotational spectroscopic parameters, the same B2/B3 approach has been validated in several studies concerning rotational constants (Spada et al., 2017a; Li et al., 2018) together with quartic centrifugal distortion parameters (Tasinato, 2014; Boussessi et al., 2020a) and, very recently, also for sextic centrifugal distortion parameters (Pietropolli Charmet et al., 2017; Boussessi et al., 2020b). In the present work, hybrid and double-hybrid density functionals of the “last-generation” are analyzed to check if they provide improved performances with respect to the B3 and B2 paradigms. This is particularly significant since the improved performances for thermochemistry, kinetics and non-covalent interactions could have been obtained at the price of worsening other parameters of particular relevance for rotational and vibrational spectroscopy of closed- and open-shell species (Puzzarini et al., 2010). In parallel, the accuracy of different partially augmented correlation consistent basis sets is analyzed with the aim of defining the best choice in terms of the cost-to-accuracy ratio. We will consider, in particular, equilibrium geometries, ground state rotational constants and quartic centrifugal distortion constants, harmonic and anharmonic vibrational wavenumbers and IR intensities. The benchmark study is carried out on a set of ten molecules of atmospheric and astrochemical relevance, reported in Figure 1, which includes: difluoromethane (CH_2_F_2_) (Carlotti et al., 1988; Tasinato et al., 2012b; Piccardo et al., 2015b), chlorofluoromethane (CH_2_ClF) (Blanco et al., 1995; Pietropolli Charmet et al., 2013), cis-1-chloro-2-fluoroethene (cis-ClHC=CHF) (Craig et al., 1970; Alonso et al., 1993; Gambi et al., 2002; Piccardo et al., 2015b), 1-chloro-1-fluoroethene (ClFC=CH_2_) (Leung et al., 2009; Pietropolli Charmet et al., 2016; Gambi et al., 2019), chlorotrifluoroethene (F_2_C=CFCl) (Hillig et al., 1988; Tasinato et al., 2012a), oxirane (cyc-C_2_H_4_O) (Russell and Wesendrup, 2003; Flaud et al., 2012; Medcraft et al., 2012; Lafferty et al., 2013; Puzzarini et al., 2014a; Piccardo et al., 2015b), glycolaldehyde (HOCH_2_CHO) (Carroll et al., 2010; Johnson et al., 2013; Piccardo et al., 2015b; Boussessi et al., 2020a), E-ethanimine (CH_3_CHNH) (Melli et al., 2018), sulfur dioxide (SO_2_) (Flaud et al., 1993; Mller and Brnken, 2005; Tasinato et al., 2010; Boussessi et al., 2020a), and the gauche conformer of ethyl mercaptan (CH_3_CH_2_SH) (Smith et al., 1968; Wolff and Szydlowski, 1985; Miller et al., 2009; Kolesnikov et al., 2014; Puzzarini et al., 2014b; Hochlaf et al., 2015).

## 2. Computational Methodology

Quantum chemical calculations at the DFT level were carried out using some hybrid and double-hybrid density functional approximations of the last generation, which are considered the best performing and transferable according to a very recent benchmark (Peveratti, 2020). Among hybrid functionals, the PW6B95 meta exchange-correlation functional proposed by Zhao and Truhlar (2005) and the ωB97 family of long-range corrected functionals introduced by Chai and Head-Gordon (Chai and Head-Gordon, 2008a,b), namely ωB97, ωB97X and ωB97X-D, were considered in conjunction with aug-cc-pVDZ (Dunning, 1989; Kendall et al., 1992; Woon and Dunning, 1993) and jul-cc-pVDZ (Papajak et al., 2011) double-ζ basis sets. For the PW6B95 functional, calculations were also carried out by using the jun-cc-pVDZ basis set (Papajak et al., 2011). The rev-DSD-PBEP86 double-hybrid density functional, recently proposed by Martin and coworkers (Santra et al., 2019), was employed together with the aug-cc-pVTZ and jun-cc-pVTZ basis sets. Indeed, triple-ζ basis sets in conjunction with the B2PLYP double-hybrid functional (Grimme, 2006) have been demonstrated to provide accurate predictions of geometries, rotational spectroscopic parameters and vibrational properties (Biczysko et al., 2010; Penocchio et al., 2015; Spada et al., 2017b; Tasinato et al., 2017; Boussessi et al., 2020a,b). Both PW6B95 and rev-DSD-PBEP86 were augmented for dispersion correlation through the Grimme's DFT-D3 scheme (Grimme et al., 2010) with Becke-Johnson damping (Grimme et al., 2011), even if the bare PW6B95 functional can already provide a satisfactory description of dispersion forces (Tasinato and Grimme, 2015). Since tight *d* functions are important for a quantitative representation of the electronic structure of second-row elements, partially augmented basis sets, namely aug-/jul-/jun-cc-pV(*n*+*d*)Z, including an additional set of *d* functions, were employed for sulfur and chlorine atoms. These basis sets were downloaded from the Basis Set Exchange library (Pritchard et al., 2019). At each level of theory, geometries were first optimized and then harmonic vibrational frequencies were computed by means of analytical Hessian matrices. While details for the calculation of analytical second-order derivatives of double-hybrid density functionals can be found in Biczysko et al. (2010), here it is only mentioned that their evaluation requires the full derivatives of the correlation contribution to the one-particle density matrix, γ^*x*^. Its occupied-occupied and virtual-virtual blocks depend on the products of second-order perturbation amplitudes and amplitude derivatives, whereas the occupied-virtual block can be found from the solution of the so-called derivative *Z*-vector equations, that involve the derivatives of the MP2 Lagrangian. The cubic and semi-diagonal quartic force constants, and the second and third derivatives of the dipole moment surface were calculated by numerical differentiation of analytic quadratic force constants and dipole moment first derivatives, respectively. These quantities were then employed for the computation of rotational and vibrational spectroscopic parameters beyond the RR/HO approximation. In particular, quartic centrifugal distortion constants, vibrational contributions to rotational constants and vibrational frequencies were derived in the framework of second-order vibrational perturbation theory (Mills, 1972; Papoušek and Aliev, 1982; Barone, 2005; Bloino et al., 2012). In order to tackle the problem of resonances plaguing the anharmonic vibrational frequencies, the generalized second-order vibrational perturbation theory (GVPT2) was adopted, in which the (near-) singular terms are removed from the perturbative summations of anharmonicity constants and transition dipole moments (leading to the so called deperturbed approach, DVPT2) and the energy levels coupled by the resonances are treated in a second step by a proper variational calculation of reduced dimensionality (Mills, 1972; Martin et al., 1995; Barone, 2005). All DFT calculations were carried out by using the Gaussian 16 suite of programs (Frisch et al., 2016), which was also employed for the perturbative treatment of the anharmonic force field in the framework of a general VPT2 engine. The latter, in addition to performing the calculation of anharmonic vibrational energies according to various flavors of VPT2 (namely, DVPT2, GVPT2 and the so-called hybrid-degeneracy corrected PT2, HDCPT2), allows the computation of transition integrals for a number of spectroscopic techniques (IR, Raman, VCD), from which the corresponding anharmonic transition intensities can be derived (Bloino et al., 2012, 2015). In addition, it should be noted that recently the VPT2 framework has been coupled to a 1-dimensional discrete-variable-representation (1D-DVR) approach for the treatment of molecular systems presenting one large-amplitude vibration (Baiardi et al., 2017), which has been applied to the simulation of the IR spectrum of the methyl-cyclopropenyl cation (Puzzarini et al., 2019b). The 1D-DVR method is currently implemented in the development version of the Gaussian code and it will be included in the next releases of the software, yet it is not required for simulating the spectroscopic properties of the molecules considered in this work, all being semi-rigid systems. Since rev-DSD-PBEP86 is not among the Gaussian built-in functionals, it has been defined by setting proper IOP flags on top of the DSD-PBEP86 functional.

## 3. Results and Discussion

The performance of last-generation density functionals (DFs) for applications in the field of rotational and vibrational spectroscopy is investigated in relation to (a) equilibrium geometry, (b) rotational spectroscopic parameters, i.e., ground state rotational constants and quartic centrifugal distortion parameters, (c) harmonic vibrational frequencies and IR intensities, and (d) fundamental anharmonic wavenumbers and IR integrated absorption cross sections. The computed data are benchmarked against values determined experimentally and, in addition, they are also compared to high-level CCSD(T)-based results taken from the literature. Statistical indicators, such as mean deviation (MD), mean absolute deviation (MAD) and mean absolute percentage deviation (MAD%) are used to assess the accuracy of the different model chemistries. For comparison purposes, B3LYP/SNSD results as well as those obtained by the B2PLYP functional in conjunction with cc-pVTZ, aug-cc-pVTZ and may′-cc-pVTZ [i.e., may-cc-pVTZ (Papajak et al., 2009) with *d* functions removed from hydrogen atoms] basis sets, obtained in a previous work (Boussessi et al., 2020a), are also reported.

### 3.1. Equilibrium Geometry

**Figure 2:**
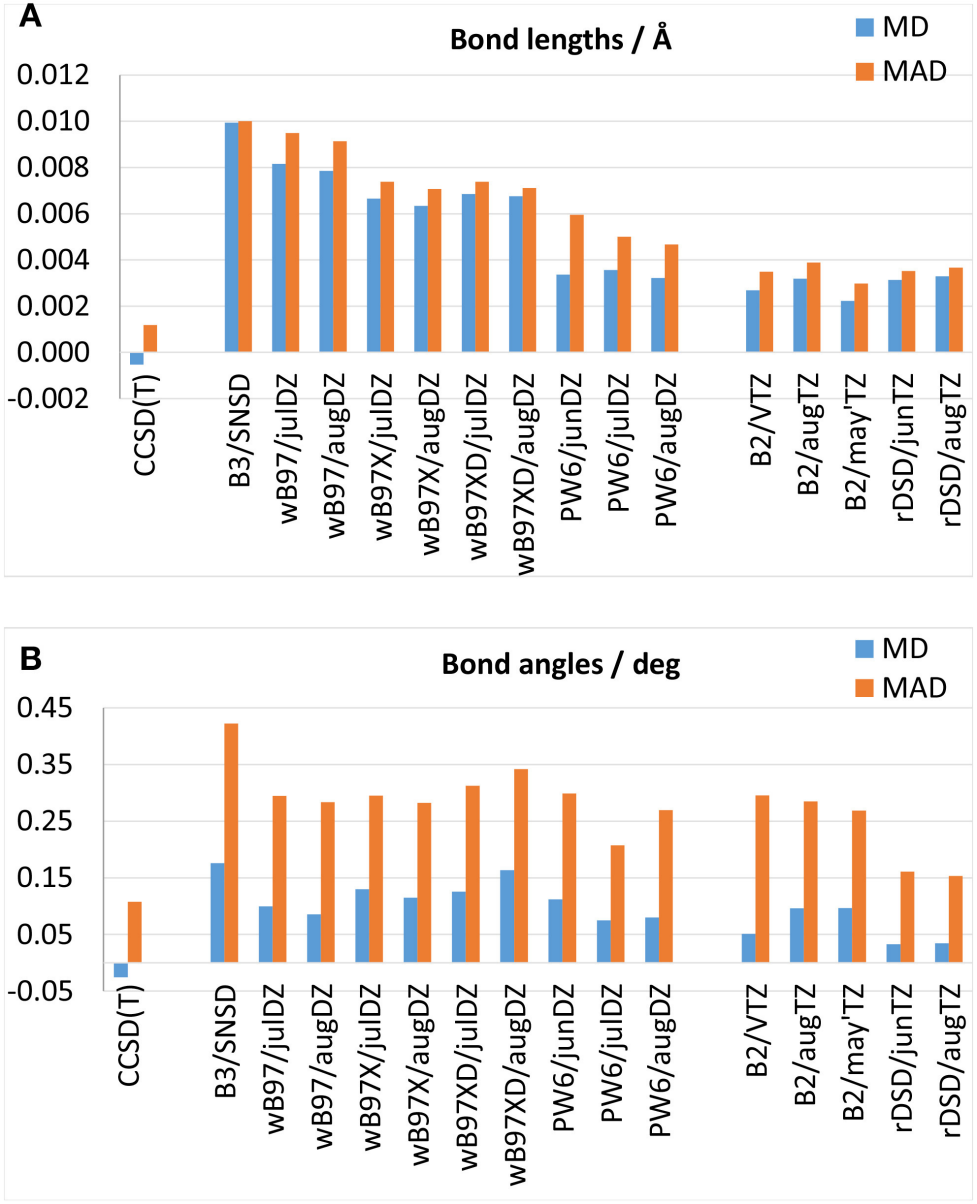
Mean deviation (MD) and mean absolute deviation (MAD) from semi-experimental equilibrium structural parameters for **(A)** bond lengths and **(B)** bond angles.

Theoretical equilibrium geometries have been benchmarked against semi-experimental equilibrium structures that, in view of their high accuracy, represent optimal reference values to test the predictive power of new computational approaches. The MDs and MADSs obtained for bond lengths and bond angles over the whole set of molecules are reported in Figures 2A,B, respectively, while the results obtained for each molecule can be found in Supplementary Tables 1–20. All the hybrid DFs well describe the equilibrium geometries, with MADs within 0.01 Å and 0.35° for bond lengths and bond angles, respectively. Interestingly, both the ωB97 family and the PW6B95 functional yield more accurate equilibrium geometries than the B3LYP/SNSD model, that during the last years, has been proposed as a good tradeoff between accuracy and computational cost to predict the structure and spectroscopic properties of medium- to large-size molecules. However, it should be noted that for the functionals belonging to the ωB97 family this improvement can be mainly attributed to the use of additional polarization functions on second-row atoms. In fact, for difluoromethane, oxirane, glycolaldehyde and ethanimine bond lengths obtained from ωB97, ωB97X and ωB97X-D functionals are in line with B3LYP/SNSD ones. Significantly more accurate bond lengths are obtained for molecules containing sulfur and chlorine atoms: in particular, it should be noted that the description of the C-S bond length of ethyl-mercaptan improves by one order of magnitude, and for SO_2_ the deviation from the semi-experimental equilibrium value for the S=O bond length decreases from 0.05 Å with the SNSD basis set to about 0.01 Å employing the ωB97(X-D) functional in conjunction with the aug-jul-cc-pV(D+d)Z basis sets. Similar conclusions can be drawn for the S-H and C-Cl bond lengths thus highlighting the importance of additional *d* functions for second-row elements. The improvement brought by PW6B95 over B3LYP is more systematic, indeed, in conjunction with aug- and jul-cc-pVDZ it attains lower deviations also for molecules containing only first-row elements. Moving to double-hybrid functionals, Figures 2A,B, demonstrate the good performance of B2PLYP and rev-DSD-PBEP86 approximations that, when coupled to triple-zeta basis sets, deliver an equivalent description of bond lengths (with MADs around 0.0035 Å), whereas for bond angles the rev-DSD-PBEP86 functional appears more accurate than B2PLYP, with excellent MDs and MADs of 0.03 and 0.15°, respectively.

### 3.2. Rotational Spectroscopic Parameters

Rotational constants are the leading terms for the prediction of rotational spectra as they rule the frequencies of the rotational transitions. While the rotational constants of the equilibrium configuration are straightforwardly derived from the equilibrium structure, for applications in the field of rotational spectroscopy, vibrational effects must be included in order to obtain the rotational constants of the molecule in a given (usually the ground) vibrational state. Even though vibrational corrections usually account only for ~1–3% of the total rotational constant value, their inclusion is mandatory for quantitative predictions of rotational spectra and, furthermore, it is necessary for the comparison with experimentally determined rotational constants. As well-known, ground state rotational constants, (*B*_0_), are obtained by adding vibrational corrections, Δ*B*_*vib*_ to equilibrium rotational constants (*B*_*e*_):

(1) $$\begin{array}{l} B_{0}^{i}=B_{e}^{i}+\Delta B_{vib}^{i}=B_{e}^{i}-\frac{1}{2}\underset{r}{\sum }\alpha_{r}^{i} \end{array}$$

where the summation runs over all the normal modes of vibration and the vibration-rotation interaction constants, $\alpha_{r}^{i}$, are evaluated in the framework of VPT2 (Mills, 1972). Their computation requires to go beyond the RR/HO approximation as they depend on the semi-diagonal cubic force field, i.e., the third derivatives of the potential energy. Besides vibrational effects, also centrifugal distortions need to be accounted for, especially for predicting high-energy rotational transitions which are of interest in e.g., astrophysical and atmospheric applications of rotational and high-resolution IR spectroscopy. Given the different orders of magnitude that rotational constants and centrifugal distortion parameters can have, the performance of the different levels of theory considered in this work can be more conveniently evaluated by referring to percentage deviations.

#### 3.2.1. Ground State Rotational Constants

**Figure 3:**
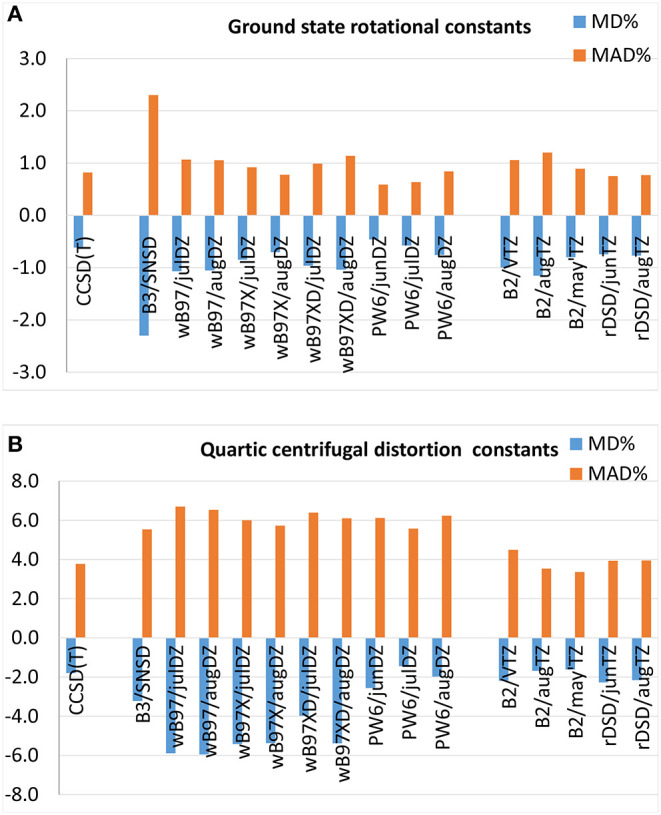
Mean percentage deviation (MD%) and mean absolute percentage deviation (MAD%) from experimental **(A)** ground state rotational constants and **(B)** quartic centrifugal distortion constants.

Mean percentage deviations and mean absolute percentage deviations from experimental ground state rotational constants are reported in Figure 3A and the full list of results can be found in Supplementary Tables 21–30. At first, it should be noted that CCSD(T)-based computations, B3LYP/SNSD and B2PLYP/triple-ζ levels of theory reproduce experimental values with MAD%s around 0.8, 2.3, and 1%, respectively. However, the average errors for CCSD(T) computations may be slightly overestimated due to the inaccuracy of the rotational constants of cis-ClHC=CHF computed by a scaled-CCSD(T) force field, in particular *A*_0_ which displays a deviation of −12% with respect to the experimental value. Indeed, by discarding this molecule, the global MD% and MAD% for CCSD(T)-based methods drop to −0.1 and 0.3%, respectively. The last-generation DFs examined in the present work provide excellent results in the prediction of ground state rotational constants: indeed, the ωB97 family shows MD%s and MAD%s around 1% and the PW6B95 functional performs even better, the MAD% being around 0.6% when coupled to the jun-cc-pVDZ and jul-cc-pVDZ basis sets and 0.8% in conjunction with the aug-cc-pVTZ basis set. A similar accuracy is delivered by the rev-DSD-PBEP86 double-hybrid functional, whose MAD% of 0.7% slightly improves the performance of the B2PLYP functional.

#### 3.2.2. Centrifugal Distortion Constants

As shown in Figure 3B, quartic centrifugal distortion constants are reproduced by hybrid DFs with MAD%s around 6–7% independently of the functional or basis set employed (the full list of results is reported in Supplementary Tables 31–40). Despite the similar performance, the last generation DFs considered in the present work perform slightly worse than the B3LYP/SNSD model, with the exception of the PW6B95/jul-cc-VDZ level of theory, which yields almost the same MAD% as B3LYP (5.5%) and a lower MD% (−2.0 vs. 3.2%), accompanied however by larger fluctuations (from −46 to 38% for PW6B95/jul-cc-pVDZ, in the −29 −19% range for B3LYP/SNSD). The rev-DSD-PBEP86 functional reproduces the quartic centrifugal distortion constants determined experimentally with a MD% and a MAD% around −2.2 and 3.9%, respectively, which are very similar to the scores of the B2PLYP functional in conjunction with augmented triple-ζ basis sets (MD% = −1.6% and MAD% = 3.6%). In passing, it should be noted that removal of diffuse functions worsens the accuracy of the results by about 1%. At this point a few remarks concerning the comparison between experimental and theoretical centrifugal distortion constants are deserved. First, it has to be noted that computed constants refer to the equilibrium configuration of the molecule whereas rotational spectroscopy measurements provide those of the ground vibrational state. Even if the vibrational dependence of centrifugal distortion parameters is expected to amount to a few percent, it is an experimentally measurable quantity. However, only a few studies have been devoted to the theoretical treatment of vibrational effects on the centrifugal distortion (Watson, 2005). Second, attention has to be paid in comparing theory and experiment because measured centrifugal distortion constants can be affected by both limited accuracy and shortcomings in the fitting procedure used for their determination, as already pointed out by Boussessi et al. (2020a,b) Indeed, during the fitting procedure, centrifugal distortion constants may absorb the effects of resonances not fully treated (in particular high-order Coriolis or anharmonic interactions) that can be difficult to describe properly within the ro-vibrational Hamiltonian employed for inverting the measured transitions.

### 3.3. Harmonic Vibrational Properties

**Figure 4:**
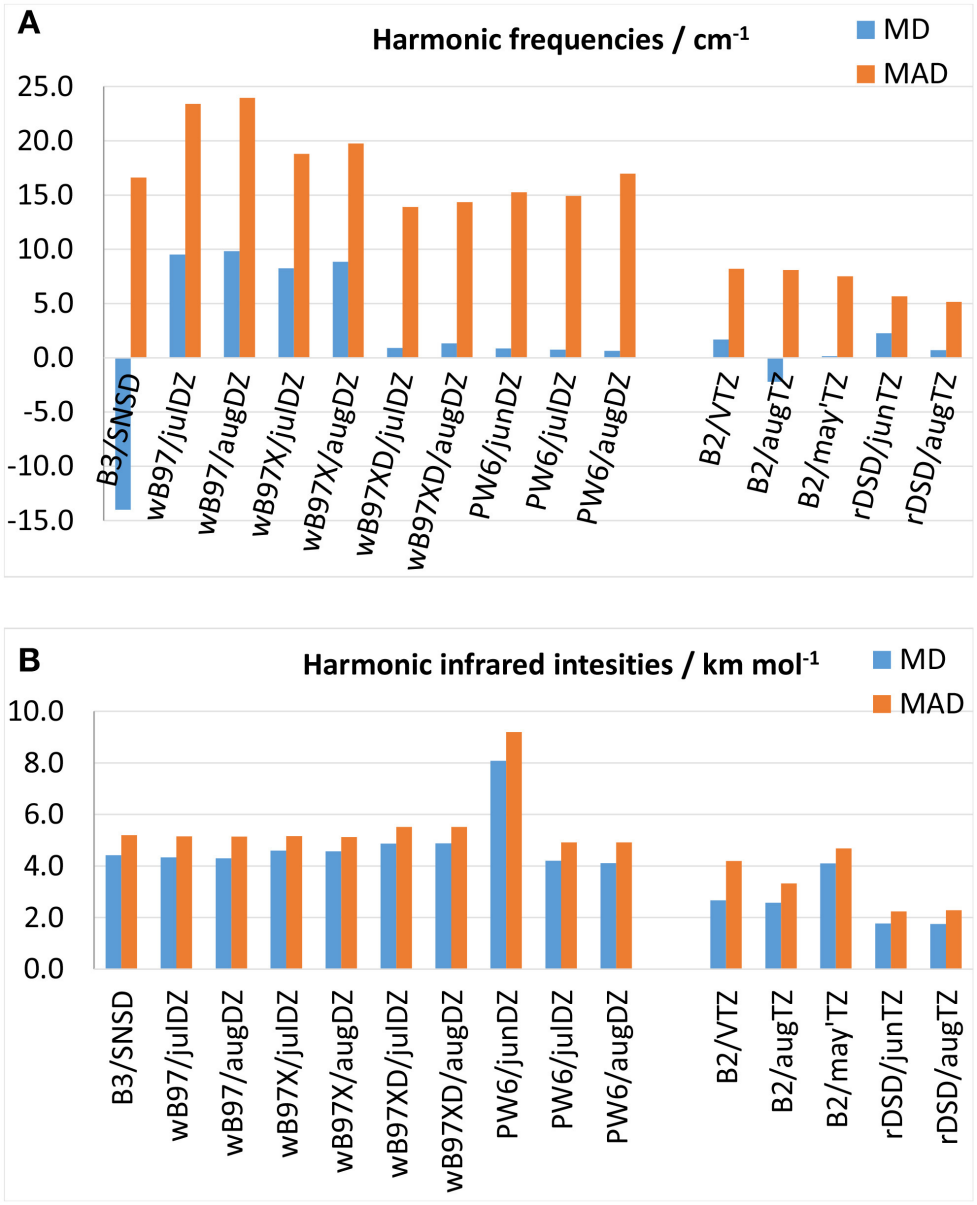
Mean deviation (MD) and mean absolute deviation (MAD) from reference CCSD(T)-based theoretical values for **(A)** harmonic vibrational frequencies, **(B)** harmonic infrared intensities.

Since experimental harmonic vibrational frequencies are not available except for a few very simple molecules, DFT predictions are here benchmarked against CCSD(T) computations performed either in conjunction with large basis sets or within composite schemes. This comparison can be justified *a posteriori* on the basis of the good agreement between high-level CCSD(T) computations and experimental anharmonic wavenumbers and integrated absorption cross sections, which imply an accurate underlying harmonic force field. In the following discussion, the gauche conformer of ethyl mercaptan has been excluded from the data set in view of the huge discrepancies, up to 113 cm^−1^, between CCSD(T)-F12 and experimental fundamental wavenumbers as discussed in (Boussessi et al., 2020a). MDs and MADs for harmonic vibrational wavenumbers and IR intensities are shown in Figures 4A,B, respectively, with the results for individual molecules being listed in the Supplementary Tables 41–59. As it can be seen from Figure 4A, concerning harmonic wavenumbers, among the functionals belonging to the ωB97 family there is a steady improvement of the performance on moving from ωB97, to ωB97X up to ωB97X-D which, with a MAD of around 14 cm^−1^ and a MD very close to zero, is the only one reaching an accuracy better than the B3 model (MAD = 16 cm^−1^). The performance of the PW6B95 functional with different basis sets is similar to that of B3LYP in conjunction with the SNSD basis set, with MADs between 15 and 17 cm^−1^ and MDs around 0.7 cm^−1^. On the other hand, the rev-DSD-PBEP86 functional represents a slight improvement over the already notable predictive power of the B2 model, being able to reproduce CCSD(T)-based reference data with a MAD of about 5 cm^−1^ to be compared with about 8 cm^−1^ at the B2PYLP level. A different picture is obtained for harmonic IR intensities: in fact, as shown in Figure 4B, all the hybrid functionals show comparable accuracy with MADs around 5 km mol^−1^, the only exception being PW6B95 in conjunction with the jun-cc-pVDZ basis set, which provides poorer results. This is probably related to the lack of diffuse *d*-functions in the basis set, whose role in the computation of IR intensities is well-known: the MAD of 9 km mol^−1^ is indeed very similar to that obtained in Boussessi et al. (2020a) for the B3LYP functional in conjunction with the pcs-1 basis set, also lacking diffuse functions. Interestingly, also for intensities the rev-DSD-PBEP86 functional, with a MAD of 2.2 km mol^−1^ performs better than B2PLYP, whose MAD in conjunction with the aug-cc-pVTZ basis set is around 3 km mol^−1^ from reference CCSD(T)-based results. From the above discussion, it can be concluded that, concerning the calculation of vibrational frequencies and IR intensities within the double-harmonic approximation, the ωB97X-D and PW6B95 hybrid functionals can represent good alternatives to the B3LYP/SNSD level of theory, and rev-DSD-PBEP86 in conjunction with triple-ζ basis sets including diffuse functions appears even more accurate than the already well-performing B2 model.

### 3.4. Beyond the Double-Harmonic Approximation: Wave-Numbers and Absorption Cross Sections

**Figure 5:**
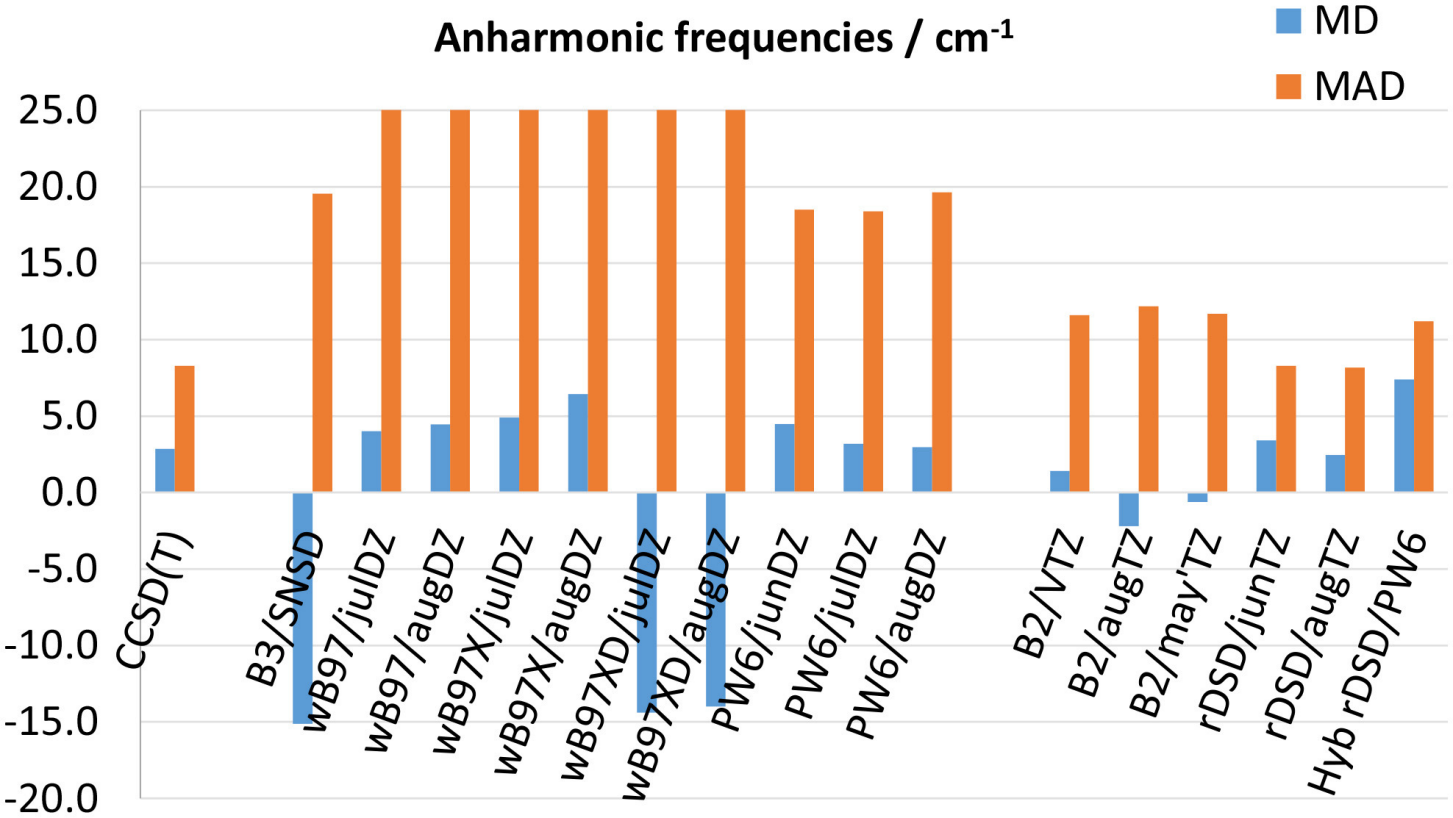
Mean deviation (MD) and mean absolute deviation (MAD) from experimental fundamental frequencies. MADs for the ωB97 family are out of range.

The performance of the DFs of the last generation considered in this work for anharmonic fundamental frequencies are compared in Figure 5 (Supplementary Tables 60–69 report the results for each molecule). It is noted that, although CCSD(T)-based computations reach a MAD of 8 cm^−1^, this result can be biased by the disagreement between experimental transition frequencies and CCSD(T)-F12/cc-pVTZ-F12 predictions previously reported for gauche-CH_3_CH_2_SH. As to the B3 and B2 models, they reproduce experimental observations with a MAD of 20 and 12 cm^−1^, respectively. In comparison, all the model chemistries based on the ωB97 approximation, showing MADs between 26 and 36 cm^−1^ cannot compete with the B3LYP functional. Given the similar results obtained for harmonic properties, it is evident that the ωB97 DFs have problems with the computation of higher order derivatives of the potential energy (i.e., cubic and semi-diagonal quartic force constants). Some of the anharmonic contributions computed for glycolaldehyde at the ωB97X-D level are particularly disappointing: the harmonic frequencies of ν_6_ and ν_8_ normal modes are predicted at about 1,445 and 1,305 cm^−1^, respectively, whereas at the ωB97X-D/jul-cc-pVDZ anharmonic level they are shifted at 904 and 719 cm^−1^ (1,012 and 832 cm^−1^ when using the aug-cc-pVTZ basis set). Even worse is the case of the ν_12_ vibration for which the anharmonic corrections evaluated by using jul-cc-pVDZ and aug-cc-pVDZ basis sets amount to −1,005 and −503 cm^−1^ thus resulting in a non-physical negative value of the ν_12_ fundamental transition frequency. Conversely, the PW6B95 DF turns out to be competitive with the B3 model, reaching a comparable MAD of 18 cm^−1^ when employed in conjunction with jul- and jun-cc-pVDZ basis sets and of 20 cm^−1^ together with the aug-cc-pVTZ basis set. Furthermore, recently Kreienborg and Merten (2019) pointed out that carbon-fluorine stretching vibrations are often strongly misplaced by common hybrid functionals with the possible exception of the M06-2X functional (restricting, however, the simulation to the double-harmonic approximation). Nevertheless, while this functional (and its predecessor M05-2X) predicts harmonic frequencies not far from experimental fundamentals, it becomes unreliable when anharmonic contributions are taken into the proper account (Puzzarini et al., 2010; Tasinato, 2014; Tasinato et al., 2018). Rather, it should be noted that the PW6B95 functional yields a consistent description of anharmonic C-F stretching frequencies, with deviations from experiment generally halved with respect to those of the B3 model. In fact, by focusing only on the C-F stretchings, the B3LYP/SNSD level of theory presents a MAD (computed over seven values) of 34 cm^−1^ and a maximum deviation of −50 cm^−1^, while the PW6B95 DF (in conjunction with all the tested double-ζ basis sets) reproduces the experimental values with a MAD around 15 cm^−1^ and a maximum error of about −25 cm^−1^. The rev-DSD-PBEP86 functional slightly improves over B2PLYP also for anharmonic fundamental wavenumbers: indeed it reproduces experimental data with a MD and a MAD around 2 and 8 cm^−1^, respectively, in comparison to −1 and 12 cm^−1^ for the B2PLYP/aug-cc-pVTZ level of theory.

**Figure 6:**
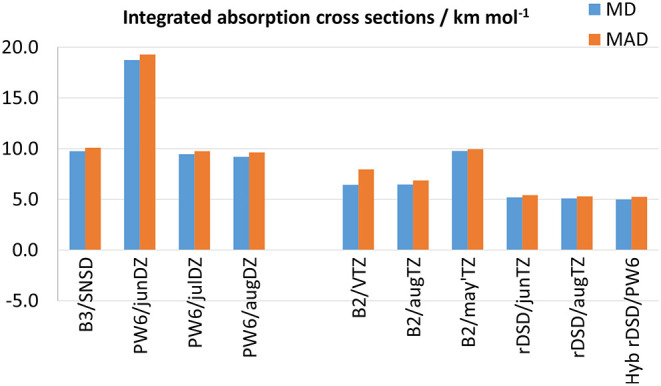
Mean deviation (MD) and mean absolute deviation (MAD) from experimental integrated absorption cross sections for selected model chemistries.

Some remarks are in order about the accuracy in the computation of IR integrated absorption cross sections (i.e., IR band intensities). It should be noted that their experimental determination is a daunting task prone to both systematic and random errors, hence a careful control of the experimental conditions and errors source needs to be performed during the measurements. Therefore, to assess the accuracy of DFT calculations, one must rely on precise experimental values, which are available only for a reduced number of molecules among those of the benchmark set, namely CH_2_F_2_, CH_2_ClF, ClFC=CH_2_, ClFC=CF_2_, and SO_2_. Moreover, in view of the poor results delivered by the ωB97-based functionals for anharmonic wave-numbers, the attention for IR anharmonic intensities is focused on PW6B95 and rev-DSD-PBEP86 DFs, whose performances are shown in Figure 6 together with those of B3 and B2 models (the full list of results is given in Supplementary Tables 70–74). In passing it should be stressed that the accuracy reached for integrated absorption cross sections depends on the reliability of both the anharmonic potential energy and dipole moment surfaces. In fact, when overlapping IR bands cannot be resolved at the experimental level, the integration required for determining band intensities is performed by considering all the absorptions within a given spectral interval. Here, the same approach has been mimicked at the theoretical level, i.e., the intensities of all the bands predicted in a given (experimental) integration range have been summed. As it can be seen, as for anharmonic wavenumbers, PW6B95 in conjunction with the aug-cc-pVDZ or jul-cc-pVDZ basis sets performs on par with B3LYP/SNSD, the MDs and MADs being around 9 and 10 km mol^−1^, respectively. Furthermore, as already pointed out for harmonic IR intensities, the lack of diffuse *d* functions in the jun-cc-pVDZ basis set results in a worsening of the predictions, thus highlighting the necessity of diffuse polarization functions for a reliable description of the dipole moment surface. Moving to the double-hybrid DFs, Figure 6 shows that, also for integrated absorption cross sections, the rev-DSD-PBEP86 functional, with both MD and MAD around 5 km mol^−1^, improves over B2PLYP that, in turn, reproduces experimental measurements with a mean absolute deviation ranging between 8 and 10 km mol^−1^ for the different triple-ζ basis sets.

Finally, anharmonic vibrational properties have been computed by a hybrid QM/QM′ approach featuring harmonic frequencies and intensities calculated at the rev-DSD-PBEP86/jul-cc-pVTZ level and anharmonic effects obtained from PW6B95/jun-cc-pVDZ computations. The hybrid force field obtained in this way simulates fundamental wavenumbers with a MAD of 11 cm^−1^, thus showing a worsening of only 3 cm^−1^ in comparison to full rev-DSD-PBEP86 computations, but still performing similarly to full B2 anharmonic predictions. This result shows that the PW6B95/jun-cc-pVDZ level of theory represents a reliable and cost-effective approach to compute anharmonic corrections when employed in conjunction with harmonic force fields obtained from the rev-DSD-PBEP86 functional. Conversely, PW6B95 should be used together with the larger jul-cc-pVDZ basis set when performing full anharmonic computations in order to obtain reliable predictions of band intensities.

### 3.5. New Model Chemistries at Work

**Figure 7:**
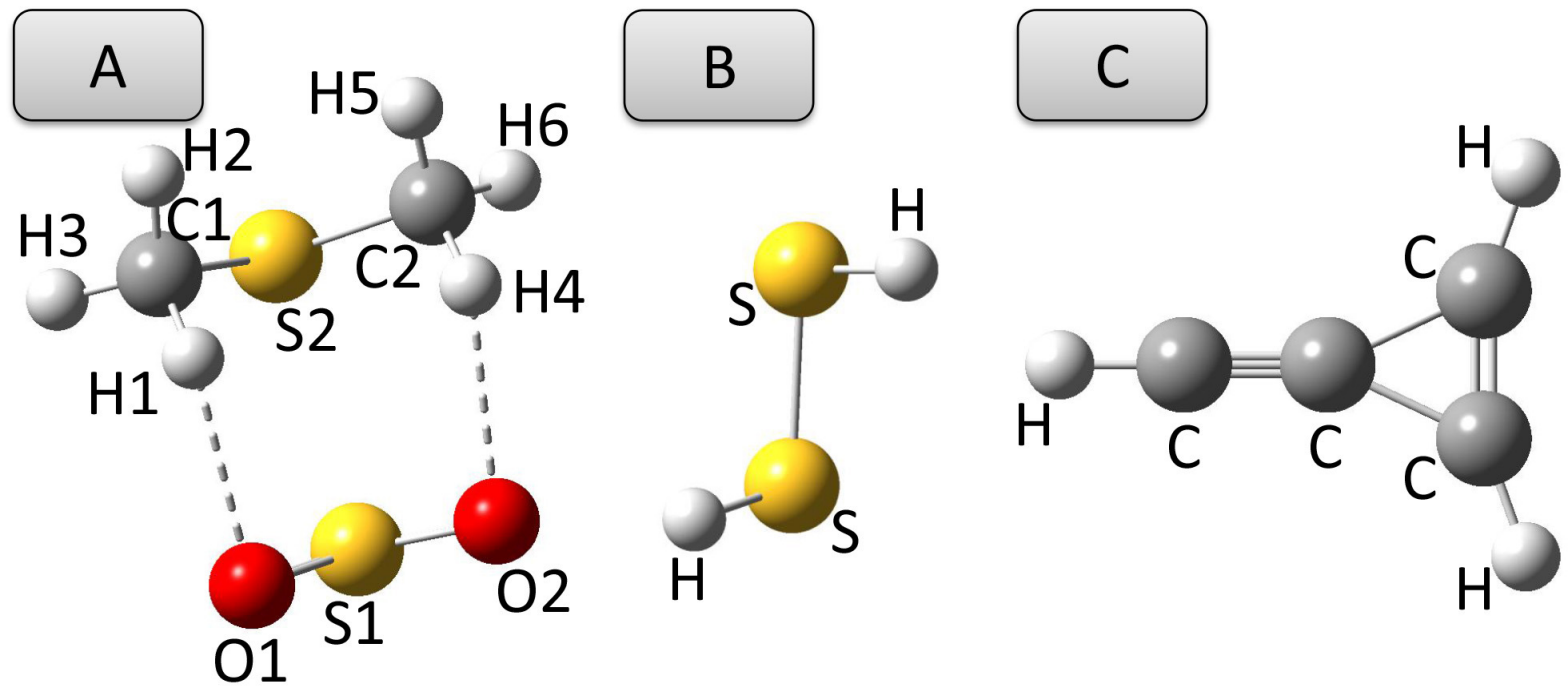
Structures of **(A)** SO_2_⋯ (CH_3_)_2_S complex, **(B)** dimethyl sulfide, and **(C)** cyc-(CH)C_3_H_2_ cation.

The previous sections have shown that, among the hybrid DFs of the last generation considered in this work, ωB97X-D and PW6B95 in conjunction with either aug- or jul-cc-pVDZ basis sets provide reliable predictions of equilibrium structures, rotational parameters and harmonic vibrational properties, sometimes even better than the well-tested B3 model. However, when anharmonic effects come into play, all the functionals of the ωB97 family yield unstable, sometimes disappointing, results. Conversely, the PW6B95 functional performs similarly to the B3 model for both fundamental transition frequencie and integrated absorption cross sections, provided that an additional set of *d* functions is employed on heavy elements, and in the case of intensities, at least a basis set of the jul-cc-pVDZ quality is used. Coming to double-hybrid functionals, the predictions of the rev-DSD-PBEP86 model are better than, or at least similar to, their B2 counterparts for both structural and rotational-vibrational spectroscopic properties.Given these premises, in this section the model chemistries able of rivaling with the B2 and B3 paradigms are applied to selected case studies in the field of structural determination and IR spectroscopy. A convenient case-study for the first subject is the complex formed by SO_2_ and dimethyl sulfide, (CH_3_)_2_S (see Figure 7A), whose semi-experimental equilibrium structure has been recently determined (Obenchain et al., 2018). Then, the anharmonic IR spectrum of the cyclic-(CH)C_3_${\text{H}}_{2}^{+}$ cation (Figure 7C) has been simulated because of the astrophysical interest of this molecule pointed out very recently by Westbook et al. (2020).

The structural parameters of the equilibrium configuration of the SO_2_⋯ (CH_3_)_2_S 1:1 complex evaluated by different DFs and basis sets are compared in Table 1 with the semi-experimental structure obtained by Obenchain et al. (2018). Inspection of this table reveals that the B3 model reproduces bond lengths, valence and dihedral angles with average absolute errors of 0.03 Å, 0.6 and 4°, respectively. The PW6B95-D3/jul-cc-pV(D+*d*)Z model chemistry delivers significantly improved results, almost halving the deviations for bond lengths and valence angles and showing an absolute average error for dihedral angles around 0.6°. Concerning the B2PLYP-D3 (employed in conjunction with the may′-cc-pVTZ basis set) and rev-DSD-PBEP86-D3 (in conjunction with the jun-cc-pV(T+*d*) basis set) functionals, it can be seen that, on average, they perform equally well for both bond lengths (the mean absolute deviation over all the complex bond lengths are 0.014 and 0.013 Å, respectively) valence (0.2 vs. 0.1°) and dihedral (0.8 and 0.7°) angles. It is also noteworthy that the hybrid PW6B95-D3 functional reproduces the semi-experimental structure of the complex with an accuracy that, in spite of the considerably reduced computational cost, rivals that of the B2 and rev-DSD-PBEP86 double-hybrids. In order to understand whether the quite large deviation observed for the inter-molecular S-S distance is due to an intrinsic inaccuracy for S-S bonds or to an unbalanced treatment of inter-molecular interactions, the equilibrium geometry of hydrogen disulfide, HSSH (Figure 7B), has been computed at the PW6B95-D3/jul-cc-pV(D+*d*)Z level and compared with the semi-experimental structure recently reported by Ye et al. (2020). The obtained structural parameters, detailed in Table 2, show deviations of 8 and 9 mÅ for the S-S and S-H bond lengths, respectively, and of 0.3° for the HSS angle, while the HSSH dihedral is within the uncertainty of the semi-experimental value. Since the SS bond length of HSSH computed at PW6B95-D3 and rev-DSD-PBEP86-D3 levels is very close, the worse performance of the former functional for the SS distance in the complex is probably related to the description of non-covalent interactions.

**Table 1:** Equilibrium structure of the SO_2_⋯ S(CH_3_) 1:1 complex^a^.

	**$r_{e}^{SE}$** ^**b**^	**B3LYP-D3**^**c**^	**PW6B95-D3**^**d**^	**B2PLYP-D3**^**e**^	**rev-DSD-PBEP86-D3**^**f**^
*r*(S1-S2)	2.947	2.8672	2.8676	2.9257	2.9288
*r*(O1-S1)	1.446	1.4966	1.4579	1.4532	1.4499
*r*(C1-S2)	1.790	1.8244	1.7954	1.8040	1.8029
*r*(H1-C1)	1.089	1.0941	1.0949	1.0883	1.0911
*r*(H2-C1)	1.086	1.0915	1.0936	1.0864	1.0895
*r*(H3-C1)	1.087	1.0922	1.0926	1.0870	1.0901
∠(O1S1S2)	95.0	96.10	95.62	95.20	94.92
∠(C1S2S1)	91.7	91.20	91.88	91.27	91.52
∠(H1C1S2)	110.6	110.08	110.72	110.67	110.60
∠(H2C1S2)	107.3	106.91	107.01	107.14	107.25
∠(H3C1S2)	109.8	109.48	109.98	109.97	109.93
δ(O2S1S2O1)	−118.2	−115.92	−117.51	−117.84	−117.86
δ(C1S2S1O1)	9.8	7.70	8.46	8.84	9.10
δ(H1C1C2S1)	27.2	21.71	26.60	26.06	27.91
δ(H2C1S2S1)	−91.6	−96.87	−91.94	−92.56	−90.77
δ(H3C1S2S1)	149.5	144.27	149.41	148.63	150.37

a Bond lengths in Å, angles in deg. For atom labeling see Figure 7.

b Semi-experimental equilibrium structure from Obenchain et al. (2018).

c Employed in conjunction with the SNSD basis set.

d Employed in conjunction with the jul-cc-pV(D+d)Z basis set.

e Employed in conjunction with the may′-cc-pVTZ basis set. From Obenchain et al. (2018).

f *Employed in conjunction with the jun-cc-pV(T+d)Z basis set*.

**Table 2:** Equilibrium structure of hydrogen disulfide^a^.

	**PW6B95-D3/**	**rev-DSDPBEP86-D3/**	**SE**^**b**^
	**jul-cc-pV(D+d)Z**	**jun-cc-pV(T+d)Z**	
*r*(S-S)	2.0596	2.0609	2.0513 (3;7)
*r*(S-H)	1.3493	1.3422	1.3401 (14;32)
∠(HSS)	98.35	98.24	98.07 (2;3)
δ(HSSH)	90.73	90.63	90.72 (2;5)

a Bond lengths in Å, angles in deg.

b Semi-experimental structure from Ye et al. (2020). Values in parentheses are the standard deviation (first value) and the confidence interval at a 95% confidence level (second value).

Moving to the vibrational properties of the cyc-(CH)C_3_${\text{H}}_{2}^{+}$ cation, harmonic frequencies are listed in Table 3 while anharmonic fundamental wavenumbers and IR intensities are reported in Table 4. In both Tables the results obtained by Westbrook et al. at the CCSD(T)-F12/aug-cc-pVTZ level (Westbook et al., 2020) are also reported for comparison purposes. In that work, the authors focused on the difficulties of post-Hartree-Fock methods in describing out-of-plane bending vibrations of molecules with C=C multiple bonds (especially, but not only, aromatic systems), an issue that should be of minor concern for DFT. For this reason, it is of some interest to compare their CCSD(T)-F12 predictions with the present DFT simulations. For hybrid DFs, the major differences with respect to CCSD(T)-F12/aug-cc-pVTZ harmonic vibrational frequencies, can be observed, for both B3LYP and PW6B95 functionals, for the ω_7_, ω_9_, ω_14_ and ω_15_ normal modes which correspond to the H-C=C-H out-of-plane bending, the ≡C-H out-of-plane vibration, the in-plane ring deformation and the in-plane bending vibration of the C-H groups, respectively. Concerning double-hybrids, the largest difference (+36 cm^−1^) is observed for ω_7_ at B2PLYP/jun-cc-pVTZ level, while the worst rev-DSDPBEP86/jun-cc-pVTZ result concerns the ω_3_ C≡C stretching vibration (+27 cm^−1^). Moving to the anharmonic vibrational wavenumbers, a huge anharmonic correction (−202 cm^−1^) for the ν_10_ fundamental and an unusual positive contribution (20 cm^−1^) for the ν_15_ vibration were obtained by Westbook et al. (2020) Conversely, according to the present calculations, the anharmonic correction for the ν_15_ vibration amounts to a few wave-numbers and that of ν_10_ ranges from about −45 cm^−1^ at the B3LYP level to −2 cm^−1^ at the B2PLYP/jun-cc-pVTZ level. While a number of Fermi resonances both of type 1 and 2 have been found, none of them strongly alters the spectral structure, just shifting the transitions by about 5 cm^−1^ from their unperturbed values. The only exception is the ν_6_ normal mode, whose excited *v*_6_ = 1 level is involved in a resonant triad together with *v*_10_ = 2 and *v*_14_ = *v*_15_ = 1.

**Table 3:** Harmonic frequencies (in cm^−1^) and intensities (in km mol^−1^) for *cyc*-CHC_3_${\text{H}}_{2}^{+}$ computed at different levels of theory.

**ω**	***Sym*.**	**B3/SNSD**		**PW6/julDZ**		**B2/junTZ**		**rDSD/junTZ**		**TZ/augVTZ**^**a**^	**MP2**^**a**^
		**ω**	***I***	**ω**	***I***	**ω**	***I***	**ω**	***I***	**ω**	***I***
ω_1_	a_1_	3,366	175.49	3,396	182.12	3,387	159.25	3,379	148.18	3370.2	121
ω_2_	a_1_	3,310	68.88	3,350	67.03	3,335	70.79	3,333	66.88	3325.9	76
ω_3_	a_1_	1,973	52.29	2,000	56.93	1,993	75.71	2,004	98.48	1977.3	186
ω_4_	a_1_	1,673	51.24	1,692	48.78	1,695	54.20	1,697	65.58	1681.3	67
ω_5_	a_1_	917	2.85	913	3.63	920	4.40	912	6.20	908.2	7
ω_6_	a_1_	798	22.40	825	19.42	772	33.55	766	42.04	768.6	63
ω_7_	a_2_	758	0.00	747	0.00	749	0.00	735	0.00	713.1	0
ω_8_	b_1_	731	65.29	723	63.95	735	65.00	731	64.92	711.5	77
ω_9_	b_1_	450	103.33	436	94.32	503	92.13	517	89.33	509.3	12
ω_10_	b_1_	361	3.08	323	13.42	382	3.55	376	4.96	355.9	0
ω_11_	b_2_	3,245	196.22	3,281	202.93	3,268	210.18	3,265	208.15	3260.5	234
ω_12_	b_2_	995	6.38	989	5.47	998	7.00	994	6.38	985.5	8
ω_13_	b_2_	944	0.69	933	0.45	937	0.04	935	0.11	920.6	3
ω_14_	b_2_	627	28.13	680	32.53	571	25.14	578	26.50	580.4	22
ω_15_	b_2_	200	24.47	190	23.01	170	26.96	160	27.56	155.7	46

a *Taken from Westbook et al. (2020)*.

**Table 4:** Anharmonic frequencies (in cm^−1^) and intensities (in km mol^−1^) for *cyc*-CHC_3_${\text{H}}_{2}^{+}$ obtained at different levels of theory.

**ν**	***Sym*.**	**B3/SNSD**		**PW6/julDZ**		**B2/junTZ**		**rDSD/junTZ**		**B2/B3**		**rDSD/PW6**		**rDSD/B3**		**TZ/aVTZ**^**a**^
		**ν**	***I***	**ν**	***I***	**ν**	***I***	**ν**	***I***	**ν**	***I***	**ν**	***I***	**ν**	***I***	**ν**
ν_1_	a_1_	3,242	89.15	3,256	213.92	3,261	188.89	3,252	159.06	3,262	188.96	3,243	217.46	3,254	202.75	3237.1
ν_2_	a_1_	3,173	47.80	3,204	25.91	3,195	32.81	3,195	43.11	3,194	27.39	3,181	17.69	3,193	27.27	3182.2
ν_3_	a_1_	1,948	32.75	1,977	43.62	1,975	58.99	1,984	77.84	1,973	66.32	1,984	43.62	1,987	33.98	1947.8
ν_4_	a_1_	1,647	45.54	1,664	41.55	1,666	33.30	1,665	57.64	1,672	41.27	1,671	45.65	1,673	47.57	1641.9
ν_5_	_*a*_1	903	3.50	897	5.08	902	0.81	902	3.55	901	1.92	873	0.31	895	1.50	884.4
ν_6_	a_1_	761	22.08	804	25.51	744	6.46	766	14.54	751	22.70	719	5.33	746	14.77	760.2
ν_7_	a_2_	743	0.00	738	0.00	736	0.00	723	0.00	735	0.00	723	0.00	720	0	713
ν_8_	b_1_	717	67.04	713	66.81	722	68.65	719	68.28	723	67.48	720	67.62	719	67.62	729
ν_9_	b_1_	410	65.38	481	96.72	496	90.31	511	87.86	473	100.36	515	92.55	487	95.23	480.8
ν_10_	b_1_	315	35.31	303	8.67	380	4.26	372	5.64	343	0.08	388	12.01	336	13.79	154.3
ν_11_	b_2_	3,117	192.57	3,146	199.69	3,141	207.44	3,140	205.27	3,141	206.76	3,129	200.04	3,138	193.33	3130.1
ν_12_	b_2_	964	5.79	962	7.78	974	8.12	968	7.34	970	8.83	966	8.11	965	9.21	970.1
ν_13_	b_2_	909	0.02	903	0.03	901	0.91	921	0.48	896	1.23	907	0.03	908	0.02	894
ν_14_	b_2_	594	25.65	653	30.44	531	21.92	539	22.88	525	22.11	530	29.72	532	25.18	547.4
ν_15_	b_2_	199	23.79	189	22.13	172	26.35	163	26.95	172	26.33	160	22.46	162	23.91	175.9

a Taken from Westbook et al. (2020).

**Figure 8:**
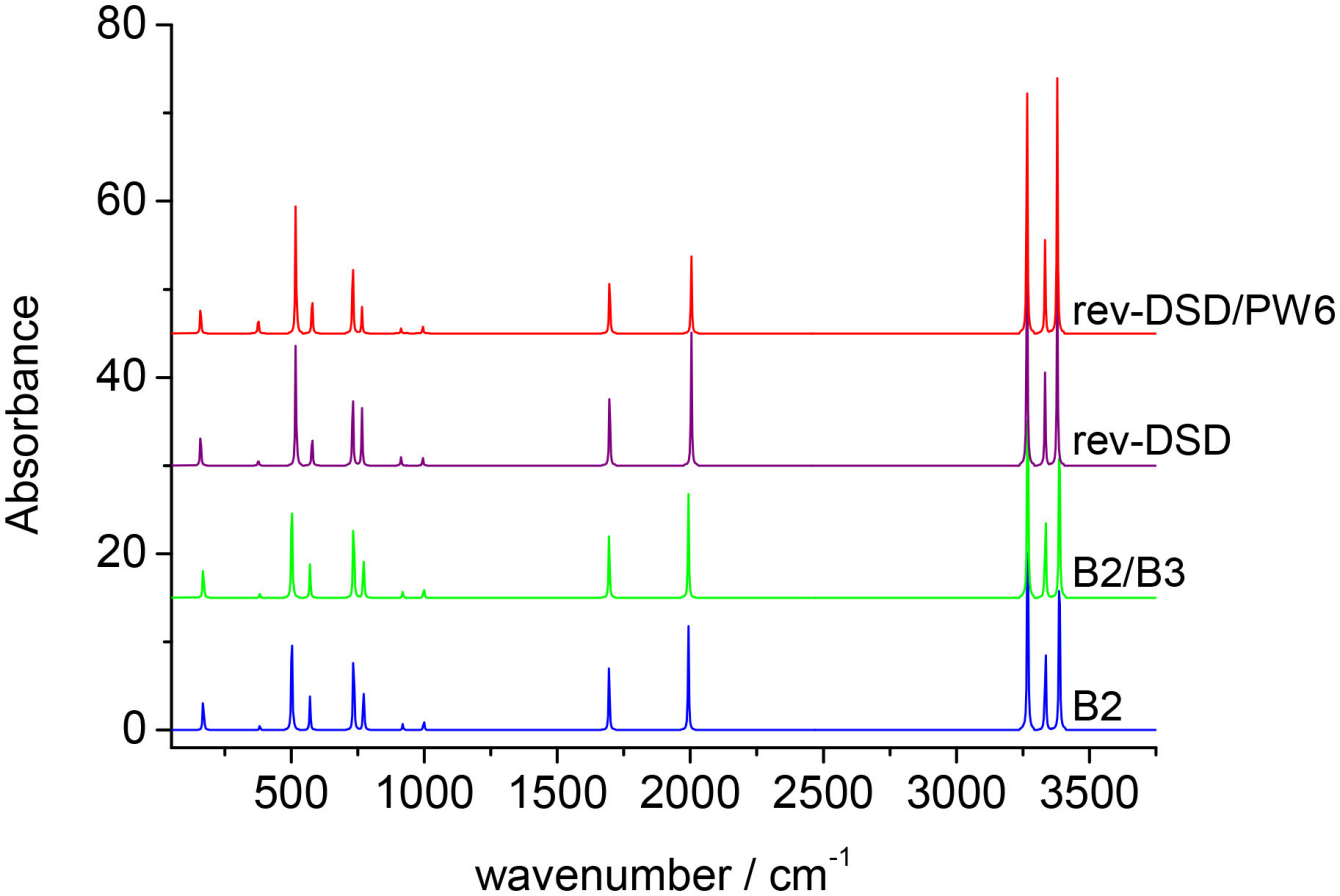
Anharmonic infrared spectrum of cyc-CHC_3_${\text{H}}_{2}^{+}$ simulated by using different levels of theory (computed spectral transitions have been convoluted with a Lorentzian function with an half-width at half-maximum of 2 cm^−1^). B2: full B2PLYP/jun-cc-pVTZ anharmonic force field; B2/B3: B2PLYP/jun-cc-pVTZ harmonic force field and B3LYP/SNSD anharmonic effects; rev-DSD: full rev-DSDPBEP86/jun-cc-pVTZ anharmonic force field; rev-DSD/PW6: rev-DSDPBEP86/jun-cc-pVTZ harmonic force field and PW6B95/jul-cc-pVDZ anharmonic effects. Some traces have been displaced fpr clarity.

Three different hybrid force fields have been also computed: in the B2/B3 hybrid force field, B2PLYP/jun-cc-pVTZ harmonic frequencies have been corrected by B3LYP/SNSD anharmonic contributions, while in the revDSD/B3 and revDSD/PW6 hybrid QM/QM′ approaches, the harmonic force field at the rev-DSD-PBEP86/jun-cc-pVTZ level has been coupled to cubic- and semi-diagonal quartic force constants evaluated at B3LYP/SNSD and PW6B95/jul-cc-pVDZ levels, respectively. The results collected in Table 4 show that the hybrid force fields deliver, with a few exceptions, the same results as the corresponding force field obtained by full anharmonic calculations at B2PLYP or rev-DSD-PBEP86 levels. The spectra of cyc-(CH)C_3_${\text{H}}_{2}^{+}$ cation, simulated beyond the double-harmonic approximation at different levels of theory, which can be useful to guide experimental measurements on this species or even astronomical campaigns, are reported in Figure 8. It is quite apparent that the only major differences among the different theoretical models are found for the integrated absorption cross sections, whereas transition frequencies are only marginally affected.

## 4. Conclusions

In the last decade we have witnessed the increasing accuracy of methods rooted in the density functional theory due to ongoing improvements in both methodological and numerical aspects. However, attention has mainly been focused on thermochemistry and kinetics, whereas theoretical support to rotational and vibrational spectroscopy requires accurate predictions of molecular geometries, harmonic force fields and leading anharmonic contributions, not to mention dipole moments and their derivatives. On these grounds, the present work has analyzed the performance of last-generation hybrid and double-hybrid functionals in conjunction with partially augmented correlation consistent basis sets for a benchmark set of 10 molecules of both atmospheric and astrochemical relevance. Equilibrium molecular geometries issued from DFT computations have been benchmarked against accurate semi-experimental equilibrium structures, while rotational constants, centrifugal distortion parameters, and vibrational frequencies have been compared to the experimental data available in the literature and with high level CCSD(T)-based *ab* *initio* calculations. The following conclusions can be drawn:(1) The jun-cc-pVDZ basis set performs remarkably well for hybrid functionals with the possible exception of IR intensities, which require diffuse *d*-functions, namely the jul-cc-pVDZ (or SNSD) basis set. In the case of double-hybrid functionals, the jun-cc-pVTZ basis set represents a nearly optimum cost/performance balance, but also the may−-cc-pVTZ basis set can be safely employed for larger systems. An additional set of *d*-functions is always mandatory for second-row atoms.(2) Among hybrid functionals, B3LYP-D3 (B3) is still very competitive, although the PW6B95 (PW6) model significantly improves equilibrium geometries.(3) Concerning double-hybrid functionals, the rev-DSD-PBEP86-D3 functional (rDSD) systematically improves the already reliable results delivered by the B2PLYP (B2) model, the enhancement being especially significant for non-covalent complexes. (4) Composite methods employing geometries and harmonic contributions evaluated by double-hybrid functionals coupled to anharmonic corrections, obtained with hybrid functionals, always lead to accurate results. In this connection the previously employed B2/B3 model remains very useful, but the new rDSD/PW6 variant seems capable of delivering even better results with the same cost.

In summary, even with further pending developments and validation, thanks to effective implementations in general electronic structure codes, last-generation hybrid and double-hybrid functionals provide unprecedented accuracy for all the parameters ruling rotational and vibrational spectroscopy with computer requirements well within current standards and, coupled to generalized second-order vibrational perturbation theory (GVPT2), can also be used by non-specialists to complement experimental studies of medium- and, even, large-size molecules of current fundamental and technological interest.

## Data Availability Statement

The original contributions generated for the study are included in the article/Supplementary Material, further inquiries can be directed to the corresponding author/s.

## Author Contributions

GC and MF performed the computations and analyzed the results. NT supervised the work, analyzed the results, and wrote part of the paper. VB defined the general strategy and wrote part of the paper. All authors contributed to the article and approved the submitted version.

## Conflict of Interest

The authors declare that the research was conducted in the absence of any commercial or financial relationships that could be construed as a potential conflict of interest.
