# Supplementary material for: Accuracy Meets Interpretability for Computational Spectroscopy by Means of Hybrid and Double-Hybrid Functionals
Source: Front Chem. 2020 Oct 23;8:584203. doi: 10.3389/fchem.2020.584203 (PMC7645164; doi:10.3389/fchem.2020.584203)
Supplement: Supplementary file 1 [file Data_Sheet_1.PDF]

Supplementary Material

1 SUPPLEMENTARY DATA

1.1 Abbreviations:

- **MD**(%): Mean percentage deviation (percent).
- **MAD**(%): Mean absolute percentage deviation (percent).
- **Max Pos**: Maxim positive deviation.
- **Max Neg**: Maxim negative deviation.

1.2 Bond Lengths

Table S1. CH_2F_2 equilibrium bond lengths in Å obtained at the different levels of theory and comparison to the semi-experimental equilibrium geometry.

CH_2F_2	SE[a]	CCSD(T)[b] aug-cc-pCVQZ	B3LYP-D3 SNSD	ωB97 jul-cc-pVDZ	ωB97 aug-cc-pVDZ	ωB97X jul-cc-pVDZ	ωB97X aug-cc-pVDZ	ωB97XD jul-cc-pVDZ	ωB97XD aug-cc-pVDZ
CH	1.0868	1.0874	1.0936	1.1003	1.0995	1.0985	1.0978	1.0985	1.0978
CF	1.3532	1.3538	1.3663	1.3722	1.3721	1.3668	1.3667	1.3640	1.3638
MD		0.0006	0.0100	0.0162	0.0158	0.0127	0.0122	0.0112	0.0108
MAD		0.0006	0.0100	0.0162	0.0158	0.0127	0.0122	0.0112	0.0108
Max Pos		0.0006	0.0131	0.0190	0.0189	0.0136	0.0135	0.0117	0.0110
Max Neg		0.0006	0.0068	0.0135	0.0127	0.0117	0.0110	0.0108	0.0106

CH_2F_2	PW6B95-D3 jul-cc-pVDZ	PW6B95-D3 aug-cc-pVDZ	PW6B95-D3 jun-cc-pVTZ	B2PLYP-D3 VTZ	B2PLYP-D3 augVTZ	B2PLYP-D3 may'VTZ	rev-DSD-PBEP86-D3 jun-cc-pVTZ	rev-DSD-PBEP86-D3 aug-cc-pVTZ
CH	1.0944	1.0935	1.0963	1.0877	1.0874	1.0872	1.0899	1.0902
CF	1.3620	1.3618	1.3590	1.3576	1.3612	1.3604	1.3584	1.3590
MD	0.0082	0.0076	0.0076	0.0027	0.0043	0.0038	0.0042	0.0046
MAD	0.0082	0.0076	0.0076	0.0027	0.0043	0.0038	0.0042	0.0046
Max Pos	0.0088	0.0086	0.0095	0.0044	0.0080	0.0072	0.0052	0.0058
Max Neg	0.0076	0.0067	0.0058	0.0010	0.0006	0.0004	0.0031	0.0034

[a] Semi-experimental equilibrium geometry, from Ref. (Piccardo et al., 2015) [b] a.e.-CCSD(T)/aug-cc-pCVQZ, from Ref. (Tasinato et al., 2012b)

Table S2. CH_2FCl equilibrium bond lengths in Å obtained at the different levels of theory and comparison to the semi-experimental equilibrium geometry.

CH_2FCl	SE[a]	CCSD(T)[b] CBS + CV + aug	B3LYP-D3 SNSD	ωB97 jul-cc-pVDZ	ωB97 aug-cc-pVDZ	ωB97X jul-cc-pVDZ	ωB97X aug-cc-pVDZ	ωB97XD jul-cc-pVDZ	ωB97XD aug-cc-pVDZ
CH	1.0840	1.0847	1.0898	1.0976	1.0969	1.0957	1.0950	1.0954	1.0948
CF	1.3594	1.3620	1.3676	1.3808	1.3806	1.3737	1.3734	1.3684	1.3679
CCl	1.7641	1.7622	1.7989	1.7545	1.7549	1.7624	1.7629	1.7745	1.7751
MD		0.0005	0.0163	0.0085	0.0083	0.0081	0.0079	0.0103	0.0101
MAD		0.0017	0.0163	0.0149	0.0144	0.0092	0.0087	0.0103	0.0101
Max Pos		0.0026	0.0347	0.0214	0.0212	0.0143	0.0140	0.0114	0.0110
Max Neg		-0.0019	0.0058	-0.0096	-0.0092	-0.0017	-0.0012	0.0090	0.0086

CH_2FCl	PW6B95-D3 jul-cc-pVDZ	PW6B95-D3 aug-cc-pVDZ	PW6B95-D3 jun-cc-pVTZ	B2PLYP-D3 VTZ	B2PLYP-D3 augVTZ	B2PLYP-D3 may'VTZ	rev-DSD-PBEP86-D3 jun-cc-pVTZ	rev-DSD-PBEP86-D3 aug-cc-pVTZ
CH	1.0912	1.0905	1.0935	1.0839	1.0839	1.0840	1.0869	1.0870
CF	1.3678	1.3673	1.3645	1.3603	1.3653	1.3652	1.3638	1.3644
CCl	1.7666	1.7672	1.7658	1.7830	1.7812	1.7764	1.7723	1.7721
MD	0.0060	0.0058	0.0054	0.0066	0.0076	0.0060	0.0052	0.0053
MAD	0.0060	0.0058	0.0054	0.0066	0.0077	0.0060	0.0052	0.0053
Max Pos	0.0084	0.0079	0.0095	0.0189	0.0171	0.0123	0.0082	0.0080
Max Neg	0.0024	0.0030	0.0016	-0.0001	-0.0001	-0.0000	0.0029	0.0031

[a] Semi-experimental equilibrium geometry, from Ref. (Pietropolli Charmet et al., 2013)
[b] CCSD(T)/CBS + Δ(CV) + Δ(aug), from Ref. (Pietropolli Charmet et al., 2013)

Table S3. *cis*-ClHC=CHF equilibrium bond lengths in Å obtained at the different levels of theory and comparison to the semi-experimental equilibrium geometry.

cis-ClHC=CHF	SE[a]	CCSD(T)[b] CBS + CV	B3LYP-D3 SNSD	ωB97 jul-cc-pVDZ	ωB97 aug-cc-pVDZ	ωB97X jul-cc-pVDZ	ωB97X aug-cc-pVDZ	ωB97XD jul-cc-pVDZ	ωB97XD aug-cc-pVDZ
C1Cl	1.7128	1.7107	1.7394	1.7113	1.7114	1.7152	1.7153	1.7211	1.7212
C1H1	1.0776	1.0764	1.0821	1.0899	1.0895	1.0880	1.0876	1.0874	1.0870
C2F	1.3317	1.3310	1.3410	1.3486	1.3484	1.3424	1.3422	1.3380	1.3377
C2H2	1.0802	1.0787	1.0851	1.0917	1.0912	1.0898	1.0893	1.0893	1.0888
C1C2	1.3240	1.3249	1.3284	1.3284	1.3282	1.3267	1.3265	1.3278	1.3276
MD		-0.0009	0.0099	0.0087	0.0085	0.0072	0.0069	0.0075	0.0072
MAD		0.0013	0.0099	0.0093	0.0090	0.0072	0.0069	0.0075	0.0072
Max Pos		0.0009	0.0266	0.0169	0.0167	0.0107	0.0105	0.0098	0.0094
Max Neg		-0.0021	0.0044	-0.0015	-0.0014	0.0024	0.0025	0.0038	0.0036

cis-ClHC=CHF	PW6B95-D3 jul-cc-pVDZ	PW6B95-D3 aug-cc-pVDZ	PW6B95-D3 jun-cc-pVTZ	B2PLYP-D3 VTZ	B2PLYP-D3 augVTZ	B2PLYP-D3 may'VTZ	rev-DSD-PBEP86-D3 jun-cc-pVTZ	rev-DSD-PBEP86-D3 aug-cc-pVTZ
C1Cl	1.7130	1.7131	1.7118	1.7246	1.7243	1.7193	1.7175	1.7177
C1H1	1.0836	1.0831	1.0855	1.0761	1.0763	1.0765	1.0791	1.0792
C2F	1.3376	1.3374	1.3355	1.3338	1.3366	1.3360	1.3345	1.3350
C2H2	1.0852	1.0846	1.0872	1.0791	1.0789	1.0790	1.0814	1.0816
C1C2	1.3255	1.3253	1.3264	1.3239	1.3241	1.3246	1.3275	1.3273
MD	0.0037	0.0034	0.0040	0.0022	0.0028	0.0018	0.0028	0.0029
MAD	0.0037	0.0034	0.0044	0.0033	0.0038	0.0027	0.0028	0.0029
Max Pos	0.0060	0.0057	0.0079	0.0118	0.0115	0.0065	0.0047	0.0049
Max Neg	0.0002	0.0003	-0.0010	-0.0015	-0.0013	-0.0012	0.0012	0.0013

[a] Semi-experimental equilibrium geometry, from Ref. (Piccardo et al., 2015)
[b] CCSD(T)/CBS + Δ(CV), from Ref. (Piccardo et al., 2015)

Table S4. ClFC=CH_2 equilibrium bond lengths in Å obtained at the different levels of theory and comparison to the semi-experimental equilibrium geometry.

ClFC=CH_2	SE[a]	CCSD(T)[b]	B3LYP-D3	ωB97		ωB97X		ωB97XD	
		CBS + CV	SNSD	jul-cc-pVDZ	aug-cc-pVDZ	jul-cc-pVDZ	aug-cc-pVDZ	jul-cc-pVDZ	aug-cc-pVDZ
C2F1	1.3287	1.3283	1.3374	1.3473	1.3470	1.3407	1.3405	1.3360	1.3358
C2C3	1.3233	1.3216	1.3250	1.3249	1.3247	1.3232	1.3230	1.3244	1.3242
C3H4	1.0780	1.0777	1.0840	1.0901	1.0894	1.0886	1.0878	1.0881	1.0874
C2Cl5	1.7081	1.7081	1.7377	1.7082	1.7084	1.7124	1.7127	1.7195	1.7198
C3H6	1.0750	1.0754	1.0813	1.0883	1.0876	1.0865	1.0858	1.0859	1.0852
MD		-0.0004	0.0105	0.0091	0.0088	0.0077	0.0073	0.0082	0.0079
MAD		0.0006	0.0105	0.0091	0.0088	0.0077	0.0075	0.0082	0.0079
Max Pos		0.0004	0.0296	0.0186	0.0183	0.0120	0.0118	0.0114	0.0117
Max Neg		-0.0017	0.0017	0.0001	0.0003	-0.0001	-0.0003	0.0011	0.0009

ClFC=CH_2	PW6B95-D3			B2PLYP-D3			rev-DSD-PBEP86-D3	
	jul-cc-pVDZ	aug-cc-pVDZ	jun-cc-pVTZ	VTZ	augVTZ	may'VTZ	jun-cc-pVTZ	aug-cc-pVTZ
C2F1	1.3362	1.3360	1.3346	1.3307	1.3339	1.3338	1.3322	1.3326
C2C3	1.3221	1.3219	1.3229	1.3208	1.3208	1.3213	1.3243	1.3241
C3H4	1.0841	1.0833	1.0856	1.0777	1.0778	1.0778	1.0803	1.0805
C2Cl5	1.7108	1.7111	1.7106	1.7239	1.7225	1.7176	1.7156	1.7157
C3H6	1.0817	1.0809	1.0833	1.0749	1.0752	1.0751	1.0779	1.0780
MD	0.0044	0.0040	0.0048	0.0030	0.0034	0.0025	0.0035	0.0036
MAD	0.0048	0.0046	0.0049	0.0042	0.0045	0.0034	0.0035	0.0036
Max Pos	0.0075	0.0073	0.0083	0.0158	0.0144	0.0095	0.0075	0.0076
Max Neg	-0.0011	-0.0013	-0.0004	-0.0025	-0.0025	-0.0020	0.0010	0.0008

[a] Semi-experimental equilibrium geometry, from Ref. (Gambi et al., 2019)
[b] CCSD(T)/CBS + Δ(CV), from Ref. (Gambi et al., 2019)

Table S5. $ClFC{=}CF_2$ equilibrium bond lengths in Å obtained at the different levels of theory and comparison to the semi-experimental equilibrium geometry.

$ClFC{=}CF_2$	SE	CCSD(T)[a] ANO2	B3LYP-D3 SNSD	ωB97 jul-cc-pVDZ	ωB97 aug-cc-pVDZ	ωB97X jul-cc-pVDZ	ωB97X aug-cc-pVDZ	ωB97XD jul-cc-pVDZ	ωB97XD aug-cc-pVDZ
C1C2	n.a.	1.3320	1.3305	1.3286	1.3286	1.3268	1.3268	1.3284	1.3284
F1C1	n.a.	1.3150	1.3208	1.3247	1.3247	1.3197	1.3197	1.3168	1.3168
F3C2	n.a.	1.3310	1.3354	1.3436	1.3436	1.3378	1.3378	1.3337	1.3337
F2C1	n.a.	1.3130	1.3192	1.3245	1.3245	1.3191	1.3191	1.3157	1.3157
ClC2	n.a.	1.7030	1.7201	1.6929	1.6929	1.6967	1.6967	1.7030	1.7030
MD			0.0064	0.0041	0.0041	0.0012	0.0012	0.0007	0.0007
MAD			0.0070	0.0095	0.0095	0.0058	0.0058	0.0021	0.0021
Max Pos			0.0171	0.0126	0.0126	0.0068	0.0068	0.0027	0.0027
Max Neg			-0.0015	-0.0101	-0.0101	-0.0063	-0.0063	-0.0036	-0.0036

$ClFC{=}CF_2$	PW6B95-D3 jul-cc-pVDZ	PW6B95-D3 aug-cc-pVDZ	PW6B95-D3 jun-cc-pVTZ	B2PLYP-D3 VTZ	B2PLYP-D3 augVTZ	B2PLYP-D3 may'VTZ	rev-DSD-PBEP86-D3 jun-cc-pVTZ	rev-DSD-PBEP86-D3 aug-cc-pVTZ
C1C2	1.3258	1.3258	1.3271	1.3260	1.3260	1.3266	1.3289	1.3285
F1C1	1.3158	1.3158	1.3141	1.3151	1.3158	1.3153	1.3137	1.3141
F3C2	1.3332	1.3332	1.3312	1.3312	1.3326	1.3325	1.3309	1.3314
F2C1	1.3149	1.3149	1.3134	1.3134	1.3144	1.3139	1.3125	1.3129
ClC2	1.6942	1.6942	1.6933	1.7072	1.7054	1.7005	1.6995	1.6993
MD	-0.0020	-0.0020	-0.0030	-0.0002	0.0000	-0.0010	-0.0017	-0.0016
MAD	0.0040	0.0040	0.0032	0.0022	0.0024	0.0021	0.0017	0.0017
Max Pos	0.0022	0.0022	0.0004	0.0042	0.0024	0.0015	-0.0001	0.0004
Max Neg	-0.0089	-0.0089	-0.0097	-0.0060	-0.0060	-0.0054	-0.0035	-0.0037

[a] CCSD(T)/ANOtz, from Ref. (Tasinato et al., 2012a)

Table S6. Oxirane equilibrium bond lengths in Å obtained at the different levels of theory and comparison to the semi-experimental equilibrium geometry.

Oxirane	SE[a]	CCSD(T)[b] CBS+CV	B3LYP-D3 SNSD (PES1)	ωB97 jul-cc-pVDZ	ωB97 aug-cc-pVDZ	ωB97X jul-cc-pVDZ	ωB97X aug-cc-pVDZ	ωB97XD jul-cc-pVDZ	ωB97XD aug-cc-pVDZ
CC	1.4609	1.4606	1.4680	1.4646	1.4647	1.4642	1.4643	1.4666	1.4667
CO	1.4274	1.4263	1.4314	1.4269	1.4269	1.4250	1.4249	1.4251	1.4250
CH	1.0816	1.0817	1.0893	1.0953	1.0944	1.0936	1.0926	1.0933	1.0924
MD		-0.0004	0.0063	0.0056	0.0053	0.0043	0.0040	0.0050	0.0047
MAD		0.0005	0.0063	0.0060	0.0057	0.0059	0.0056	0.0065	0.0063
Max Pos		0.0001	0.0077	0.0137	0.0128	0.0120	0.0110	0.0117	0.0108
Max Neg		-0.0011	0.0040	-0.0005	-0.0005	-0.0024	-0.0025	-0.0023	-0.0024

Oxirane	PW6B95-D3 jul-cc-pVDZ	PW6B95-D3 aug-cc-pVDZ	PW6B95-D3 jun-cc-pVTZ	B2PYLP VTZ (PES5)	B2PLYP-D3 augVTZ (PES4)	B2PLYP-D3 may'VTZ (PES2)	rev-DSD-PBEP86-D3 jun-cc-pVTZ	rev-DSD-PBEP86-D3 aug-cc-pVTZ
CC	1.4626	1.4628	1.4622	1.4620	1.4619	1.4621	1.4636	1.4637
CO	1.4242	1.4241	1.4207	1.4301	1.4330	1.4326	1.4316	1.4319
CH	1.0892	1.0881	1.0914	1.0826	1.0824	1.0824	1.0848	1.0850
MD	0.0020	0.0017	0.0014	0.0016	0.0025	0.0024	0.0034	0.0036
MAD	0.0042	0.0039	0.0059	0.0016	0.0025	0.0024	0.0034	0.0036
Max Pos	0.0076	0.0065	0.0098	0.0027	0.0056	0.0052	0.0042	0.0045
Max Neg	-0.0032	-0.0033	-0.0067	0.0010	0.0008	0.0008	0.0027	0.0028

[a] Semi-experimental equilibrium geometry, from Ref. (Puzzarini et al., 2014a)
[b] CCSD(T)/CBS + Δ(CV), from Ref. (Puzzarini et al., 2014a)

Table S7. CH_3CH_2SH equilibrium bond lengths in Å obtained at the different levels of theory and comparison to the semi-experimental equilibrium geometry.

CH_3CH_2SH	SE	CCSD(T)[a] CBS+CV	B3LYP-D3 SNSD	ωB97 jul-cc-pVDZ	ωB97 aug-cc-pVDZ	ωB97X jul-cc-pVDZ	ωB97X aug-cc-pVDZ	ωB97XD jul-cc-pVDZ	ωB97XD aug-cc-pVDZ
C2C1	n.a.	1.5173	1.5252	1.5232	1.5234	1.5217	1.5218	1.5232	1.5229
S3C1	n.a.	1.8125	1.8500	1.8084	1.8088	1.8137	1.8141	1.8084	1.8225
H4C1	n.a.	1.0888	1.0933	1.1002	1.0991	1.0985	1.0974	1.1002	1.0971
H5C1	n.a.	1.0894	1.0945	1.1013	1.1002	1.0997	1.0987	1.1013	1.0983
H6C2	n.a.	1.0905	1.0977	1.1016	1.1007	1.1003	1.0995	1.1016	1.0996
H7C2	n.a.	1.0901	1.0955	1.1011	1.1002	1.0996	1.0988	1.1011	1.0986
H8C2	n.a.	1.0889	1.0943	1.0998	1.0989	1.0984	1.0975	1.0998	1.0973
H9S3	n.a.	1.3371	1.3547	1.3438	1.3435	1.3457	1.3455	1.3438	1.3477
MD			0.0113	0.0081	0.0075	0.0079	0.0073	0.0081	0.0087
MAD			0.0113	0.0091	0.0085	0.0079	0.0073	0.0091	0.0087
Max Pos			0.0375	0.0119	0.0108	0.0103	0.0093	0.0119	0.0106
Max Neg			0.0045	-0.0041	-0.0037	0.0012	0.0016	-0.0041	0.0056

CH_3CH_2SH	PW6B95-D3 jul-cc-pVDZ	PW6B95-D3 aug-cc-pVDZ	PW6B95-D3 jun-cc-pVTZ	B2PLYP-D3 VTZ	B2PLYP-D3 augVTZ	B2PLYP-D3 may'VTZ	rev-DSD-PBEP86-D3 jun-cc-pVTZ	rev-DSD-PBEP86-D3 aug-cc-pVTZ
C2C1	1.5161	1.5163	1.5159	1.5201	1.5200	1.5206	1.5217	1.5216
S3C1	1.8179	1.8184	1.8165	1.8305	1.8310	1.8258	1.8220	1.8218
H4C1	1.0941	1.0929	1.0965	1.0874	1.0875	1.0876	1.0905	1.0907
H5C1	1.0952	1.0941	1.0974	1.0887	1.0888	1.0889	1.0919	1.0921
H6C2	1.0964	1.0955	1.0986	1.0909	1.0911	1.0909	1.0935	1.0938
H7C2	1.0951	1.0941	1.0978	1.0892	1.0894	1.0892	1.0921	1.0924
H8C2	1.0938	1.0928	1.0692	1.0879	1.0881	1.0879	1.0908	1.0911
H9S3	1.3453	1.3451	1.3462	1.3414	1.3417	1.3391	1.3397	1.3401
MD	0.0049	0.0043	0.0030	0.0027	0.0029	0.0019	0.0035	0.0036
MAD	0.0052	0.0046	0.0082	0.0037	0.0037	0.0028	0.0035	0.0036
Max Pos	0.0082	0.0080	0.0091	0.0180	0.0185	0.0133	0.0095	0.0093
Max Neg	-0.0012	-0.0010	-0.0197	-0.0014	-0.0013	-0.0012	0.0017	0.0019

[a] CCSD(T)/CBS + Δ(CV), from Ref. (Hochlaf et al., 2015)

Table S8. SO_2 equilibrium bond lengths in Å obtained at the different levels of theory and comparison to the semi-experimental equilibrium geometry.

SO_2	SE[a]	CCSD(T)[b] CBS+CV	B3LYP-D3 SNSD	ωB97 jul-cc-pVDZ	ωB97 aug-cc-pVDZ	ωB97X jul-cc-pVDZ	ωB97X aug-cc-pVDZ	ωB97XD jul-cc-pVDZ	ωB97XD aug-cc-pVDZ
SO	1.4308	1.4311	1.4823	1.4439	1.4439	1.4429	1.4429	1.4439	1.4439
MD		0.0003	0.0514	0.0130	0.0130	0.0121	0.0121	0.0131	0.0131
MAD		0.0003	0.0514	0.0130	0.0130	0.0121	0.0121	0.0131	0.0131
Max Pos		0.0003	0.0514	0.0130	0.0130	0.0121	0.0121	0.0131	0.0131
Max Neg		0.0003	0.0514	0.0130	0.0130	0.0121	0.0121	0.0131	0.0131

SO_2	PW6B95-D3 jul-cc-pVDZ	PW6B95-D3 aug-cc-pVDZ	PW6B95-D3 jun-cc-pVTZ	B2PLYP-D3 VTZ	B2PLYP-D3 augVTZ	B2PLYP-D3 may'VTZ	rev-DSD-PBEP86-D3 jun-cc-pVTZ	rev-DSD-PBEP86-D3 aug-cc-pVTZ
SO	1.4454	1.4454	1.4421	1.4562	1.4582	1.4438	1.4421	1.4427
MD	0.0146	0.0146	0.0113	0.0254	0.0273	0.0129	0.0113	0.0118
MAD	0.0146	0.0146	0.0113	0.0254	0.0273	0.0129	0.0113	0.0118
Max Pos	0.0146	0.0146	0.0113	0.0254	0.0273	0.0129	0.0113	0.0118
Max Neg	0.0146	0.0146	0.0113	0.0254	0.0273	0.0129	0.0113	0.0118

[a] Semi-experimental equilibrium geometry, from Ref. (Boussessi et al., 2020)
[b] CCSD(T)/CBS + Δ(CV), from Ref. (Boussessi et al., 2020)

Table S9. $HCOCH_2OH$ equilibrium bond lengths in Å obtained at the different levels of theory and comparison to the semi-experimental equilibrium geometry.

$HCOCH_2OH$	SE[a]	Cheap[b]	B3LYP-D3 SNSD	ωB97 jul-cc-pVDZ	ωB97 aug-cc-pVDZ	ωB97X jul-cc-pVDZ	ωB97X aug-cc-pVDZ	ωB97XD jul-cc-pVDZ	ωB97XD aug-cc-pVDZ
C1O	1.2088	1.2064	1.2120	1.2129	1.2127	1.2102	1.2100	1.2094	1.2092
C1H	1.1010	1.0994	1.1093	1.1139	1.1131	1.1125	1.1118	1.1127	1.1119
C1C2	1.5003	1.4996	1.5061	1.5060	1.5063	1.5034	1.5037	1.5042	1.5044
C2H	1.0963	1.0941	1.1035	1.1071	1.1064	1.1061	1.1054	1.1065	1.1059
C2O	1.3964	1.3935	1.4005	1.4005	1.4005	1.3970	1.3970	1.3947	1.3946
OH	0.9611	0.9635	0.9718	0.9699	0.9699	0.9685	0.9685	0.9680	0.9680
MD		-0.0013	0.0065	0.0077	0.0075	0.0056	0.0054	0.0052	0.0050
MAD		0.0020	0.0065	0.0077	0.0075	0.0056	0.0054	0.0058	0.0056
Max Pos		0.0023	0.0107	0.0129	0.0121	0.0115	0.0108	0.0117	0.0109
Max Neg		-0.0030	0.0033	0.0040	0.0040	0.0005	0.0005	-0.0018	-0.0018

$HCOCH_2OH$	PW6B95-D3 jul-cc-pVDZ	PW6B95-D3 aug-cc-pVDZ	PW6B95-D3 jun-cc-pVTZ	B2PLYP-D3 VTZ	B2PLYP-D3 augVTZ	B2PLYP-D3 may'VTZ	rev-DSD-PBEP86-D3 jun-cc-pVTZ	rev-DSD-PBEP86-D3 aug-cc-pVTZ
C1O	1.2083	1.2080	1.2082	1.2101	1.2108	1.2108	1.2117	1.2117
C1H	1.1087	1.1078	1.1110	1.1027	1.1024	1.1025	1.1043	1.1046
C1C2	1.4974	1.4977	1.4981	1.5025	1.5022	1.5024	1.5051	1.5049
C2H	1.1028	1.1020	1.1050	1.0969	1.0967	1.0966	1.0988	1.0991
C2O	1.3928	1.3928	1.3906	1.3981	1.4002	1.4001	1.4004	1.4006
OH	0.9679	0.9678	0.9710	0.9678	0.9684	0.9685	0.9685	0.9686
MD	0.0023	0.0020	0.0033	0.0024	0.0028	0.0028	0.0041	0.0042
MAD	0.0047	0.0044	0.0062	0.0024	0.0028	0.0028	0.0041	0.0042
Max Pos	0.0077	0.0068	0.0100	0.0067	0.0073	0.0074	0.0074	0.0074
Max Neg	-0.0036	-0.0037	-0.0058	0.0006	0.0004	0.0002	0.0025	0.0028

[a] Semi-experimental equilibrium geometry, from Ref. (Piccardo et al., 2015)
[b] Cheap composite scheme, from Ref. (Boussessi et al., 2020)

Table S10. *E*-Ethanimine equilibrium bond lengths in Å obtained at the different levels of theory and comparison to the semi-experimental equilibrium geometry.

	SE	CCSD(T)[a]	B3LYP-D3	ωB97		ωB97X		ωB97XD	
E-Ethanimine		CBS+CV	SNSD	jul-cc-pVDZ	aug-cc-pVDZ	jul-cc-pVDZ	aug-cc-pVDZ	jul-cc-pVDZ	aug-cc-pVDZ
C1C2	n.a.	1.4915	1.4975	1.4985	1.4986	1.4961	1.4962	1.4962	1.4963
N3C2	n.a.	1.2723	1.2743	1.2741	1.2739	1.2721	1.2719	1.2728	1.2726
H4N3	n.a.	1.0175	1.0228	1.0235	1.0230	1.0224	1.0219	1.0229	1.0224
H5C2	n.a.	1.0936	1.1014	1.1068	1.1061	1.1052	1.1045	1.1051	1.1044
H6C1	n.a.	1.0905	1.0976	1.1015	1.1009	1.1004	1.0998	1.1006	1.1001
H8C1	n.a.	1.0864	1.0926	1.0979	1.0972	1.0965	1.0959	1.0963	1.0958
MD			0.0057	0.0084	0.0080	0.0068	0.0064	0.0070	0.0066
MAD			0.0057	0.0084	0.0080	0.0069	0.0065	0.0070	0.0066
Max Pos			0.0078	0.0132	0.0125	0.0116	0.0109	0.0115	0.0108
Max Neg			0.0020	0.0018	0.0016	-0.0002	-0.0004	0.0005	0.0003

	PW6B95-D3			B2PLYP-D3			rev-DSD-PBEP86-D3	
E-Ethanimine	jul-cc-pVDZ	aug-cc-pVDZ	jun-cc-pVTZ	VTZ	augVTZ	may'VTZ	jun-cc-pVTZ	aug-cc-pVTZ
C1C2	1.4898	1.4899	1.4898	1.4936	1.4929	1.4929	1.4954	1.4954
N3C2	1.2700	1.2697	1.2703	1.2705	1.2715	1.2717	1.2744	1.2744
H4N3	1.0200	1.0195	1.0234	1.0185	1.0183	1.0187	1.0203	1.0204
H5C2	1.1014	1.1006	1.1038	1.0946	1.0945	1.0946	1.0965	1.0968
H6C1	1.0966	1.0960	1.0988	1.0910	1.0912	1.0910	1.0936	1.0938
H8C1	1.0921	1.0914	1.0942	1.0863	1.0865	1.0863	1.0892	1.0894
MD	0.0030	0.0025	0.0048	0.0005	0.0005	0.0006	0.0029	0.0031
MAD	0.0044	0.0039	0.0060	0.0011	0.0008	0.0008	0.0029	0.0031
Max Pos	0.0078	0.0070	0.0102	0.0021	0.0014	0.0014	0.0039	0.0039
Max Neg	-0.0023	-0.0026	-0.0020	-0.0018	-0.0008	-0.0006	0.0021	0.0021

[a] CCSD(T)/CBS + Δ(CV), from Ref. (Melli et al., 2018)

1.3 Bond Angles

Table S11. CH_2F_2 bond angles in degree obtained at the different levels of theory and comparison to the semi-experimental equilibrium geometry.

CH_2F_2	SE[a]	CCSD(T)[b] aug-cc-pCVQZ	B3LYP-D3 SNSD	ωB97 jul-cc-pVDZ	ωB97 aug-cc-pVDZ	ωB97X jul-cc-pVDZ	ωB97X aug-cc-pVDZ	ωB97XD jul-cc-pVDZ	ωB97XD aug-cc-pVDZ
HCH	113.48	113.35	113.71	114.27	114.40	113.99	114.11	113.73	113.85
FCF	108.29	108.33	108.48	108.09	108.04	108.18	108.13	108.31	108.27
HCF	108.74	108.76	108.63	108.58	108.56	108.63	108.61	108.67	108.65
MD		-0.02	0.10	0.14	0.16	0.10	0.11	0.07	0.08
MAD		0.06	0.17	0.38	0.45	0.25	0.31	0.11	0.16
Max Pos		0.04	0.23	0.79	0.92	0.51	0.63	0.25	0.37
Max Neg		-0.13	-0.11	-0.20	-0.25	-0.11	-0.16	-0.07	-0.09

CH_2F_2	PW6B95-D3 jul-cc-pVDZ	PW6B95-D3 aug-cc-pVDZ	PW6B95-D3 jun-cc-pVDZ	B2PLYP-D3 VTZ	B2PLYP-D3 augVTZ	B2PLYP-D3 may'VTZ	rev-DSD-PBEP86-D3 jun-cc-pVTZ	rev-DSD-PBEP86-D3 aug-cc-pVTZ
HCH	113.83	113.93	113.66	113.16	113.66	113.57	113.51	113.54
FCF	108.21	108.16	108.25	108.63	108.37	108.44	108.35	108.32
HCF	108.67	108.65	108.70	108.74	108.67	108.68	108.72	108.72
MD	0.07	0.08	0.03	0.01	0.06	0.06	0.02	0.02
MAD	0.17	0.22	0.08	0.22	0.11	0.10	0.04	0.04
Max Pos	0.35	0.45	0.18	0.34	0.18	0.15	0.06	0.06
Max Neg	-0.08	-0.13	-0.04	-0.32	-0.07	-0.06	-0.02	-0.02

[a] Semi-experimental equilibrium geometry, from Ref. (Piccardo et al., 2015)
[b] a.e.-CCSD(T)/aug-cc-pCVQZ, from Ref. (Tasinato et al., 2012b)

Table S12. CH_2FCl bond angles in degree obtained at the different levels of theory and comparison to the semi-experimental equilibrium geometry.

	SE[a]	CCSD(T)[b]	B3LYP-D3	ωB97		ωB97X		ωB97XD	
CH_2FCl		CBS + CV + aug	SNSD	jul-cc-pVDZ	aug-cc-pVDZ	jul-cc-pVDZ	aug-cc-pVDZ	jul-cc-pVDZ	aug-cc-pVDZ
HCCl	107.94	108.18	107.60	108.69	108.69	108.42	108.41	108.05	108.03
HCH	112.56	112.72	113.18	112.68	112.78	112.71	112.81	112.81	112.93
FCCl	110.05	109.89	110.17	109.86	109.82	109.91	109.88	110.02	110.00
MD		0.08	0.13	0.22	0.24	0.16	0.18	0.11	0.13
MAD		0.19	0.36	0.35	0.40	0.25	0.29	0.13	0.17
Max Pos		0.23	0.62	0.74	0.74	0.48	0.47	0.25	0.37
Max Neg		-0.16	-0.34	-0.19	-0.23	-0.14	-0.17	-0.02	-0.05

	PW6B95-D3			B2PLYP-D3			rev-DSD-PBEP86-D3	
CH_2FCl	jul-cc-pVDZ	aug-cc-pVDZ	jun-cc-pVDZ	VTZ	augVTZ	may'VTZ	jun-cc-pVTZ	aug-cc-pVTZ
HCCl	108.15	108.15	108.16	107.54	107.78	107.92	107.99	107.94
HCH	112.86	112.95	112.55	112.67	112.93	112.73	112.64	112.70
FCCl	109.97	109.94	110.15	110.36	110.17	110.26	110.13	110.11
MD	0.14	0.16	0.10	0.00	0.11	0.12	0.07	0.07
MAD	0.19	0.23	0.11	0.28	0.22	0.13	0.07	0.07
Max Pos	0.29	0.38	0.21	0.32	0.37	0.21	0.09	0.14
Max Neg	-0.08	-0.11	-0.01	-0.41	-0.17	-0.02	0.04	-0.00

[a] Semi-experimental equilibrium geometry, from Ref. (Pietropolli Charmet et al., 2013)
[b] CCSD(T)/CBS + Δ(CV) + Δ(aug), from Ref. (Pietropolli Charmet et al., 2013)

Table S13. *cis*-ClHC=CHF bond angles in degree obtained at the different levels of theory and comparison to the semi-experimental equilibrium geometry.

	SE[a]	CCSD(T)[b]	B3LYP-D3	ωB97		ωB97X		ωB97XD	
cis-ClHC=CHF		CBS + CV	SNSD	jul-cc-pVDZ	aug-cc-pVDZ	jul-cc-pVDZ	aug-cc-pVDZ	jul-cc-pVDZ	aug-cc-pVDZ
H1C1C2	120.74	120.43	120.90	120.29	120.28	120.41	120.40	120.37	120.37
ClC1C2	123.07	123.10	123.75	123.34	123.40	123.41	123.48	123.71	123.77
H2C2C1	123.50	123.43	123.44	123.98	123.99	123.71	123.74	123.23	123.28
FC2C1	122.61	122.53	123.08	122.56	122.52	122.74	122.68	123.14	123.06
MD		-0.11	0.31	0.06	0.07	0.09	0.09	0.13	0.14
MAD		0.12	0.34	0.31	0.35	0.25	0.26	0.45	0.44
Max Pos		0.03	0.68	0.48	0.49	0.34	0.41	0.64	0.70
Max Neg		-0.31	-0.06	-0.45	-0.46	-0.33	-0.34	-0.37	-0.37

	PW6B95-D3			B2PLYP-D3			rev-DSD-PBEP86-D3	
cis-ClHC=CHF	jul-cc-pVDZ	aug-cc-pVDZ	jun-cc-pVDZ	VTZ	augVTZ	may'VTZ	jun-cc-pVTZ	aug-cc-pVTZ
H1C1C2	120.48	120.47	120.31	120.55	120.48	120.29	120.25	120.31
ClC1C2	123.45	123.52	123.53	123.50	123.56	123.66	123.44	123.41
H2C2C1	123.57	123.61	123.48	123.00	123.31	123.30	123.26	123.28
FC2C1	122.75	122.69	122.82	123.20	122.99	123.02	122.87	122.87
MD	0.08	0.09	0.06	0.08	0.11	0.09	-0.02	-0.02
MAD	0.21	0.23	0.28	0.43	0.33	0.41	0.34	0.31
Max Pos	0.38	0.45	0.46	0.59	0.49	0.59	0.37	0.34
Max Neg	-0.26	-0.27	-0.43	-0.50	-0.26	-0.45	-0.49	-0.43

[a] Semi-experimental equilibrium geometry, from Ref. (Piccardo et al., 2015)
[b] CCSD(T)/CBS + Δ(CV), from Ref. (Piccardo et al., 2015)

Table S14. ClFC=CH_2 bond angles in degree obtained at the different levels of theory and comparison to the semi-experimental equilibrium geometry.

ClFC=CH_2	SE[a]	CCSD(T)[b] CBS + CV	B3LYP-D3 SNSD	ωB97 jul-cc-pVDZ	ωB97 aug-cc-pVDZ	ωB97X jul-cc-pVDZ	ωB97X aug-cc-pVDZ	ωB97XD jul-cc-pVDZ	ωB97XD aug-cc-pVDZ
C3C2F1	122.44	122.48	122.75	122.31	122.33	122.45	122.47	122.67	122.68
C3C2Cl5	125.52	125.59	125.77	126.28	126.24	126.08	126.05	125.82	125.80
C2C3H6	120.02	119.96	120.59	120.00	119.99	120.14	120.13	120.27	120.26
C2C3H4	119.30	119.32	119.49	119.35	119.45	119.39	119.50	119.45	119.55
MD		0.02	0.33	0.16	0.18	0.20	0.22	0.23	0.25
MAD		0.05	0.33	0.24	0.25	0.20	0.22	0.23	0.25
Max Pos		0.07	0.57	0.76	0.72	0.56	0.53	0.30	0.28
Max Neg		-0.06	0.19	-0.13	-0.11	0.01	0.03	0.15	0.24

ClFC=CH_2	PW6B95-D3 jul-cc-pVDZ	PW6B95-D3 aug-cc-pVDZ	PW6B95-D3 jun-cc-pVDZ	B2PLYP-D3 VTZ	B2PLYP-D3 augVTZ	B2PLYP-D3 may'VTZ	rev-DSD-PBEP86-D3 jun-cc-pVTZ	rev-DSD-PBEP86-D3 aug-cc-pVTZ
C3C2F1	122.49	122.49	122.49	122.81	122.63	122.49	122.52	122.57
C3C2Cl5	126.04	126.02	126.05	125.43	125.73	125.77	125.61	125.60
C2C3H6	120.27	120.26	120.29	120.41	120.30	120.37	120.13	120.06
C2C3H4	119.41	119.52	119.32	119.31	119.40	119.43	119.37	119.41
MD	0.23	0.25	0.22	0.17	0.20	0.19	0.09	0.09
MAD	0.23	0.25	0.22	0.21	0.20	0.19	0.09	0.09
Max Pos	0.52	0.50	0.53	0.39	0.28	0.35	0.11	0.13
Max Neg	0.05	0.05	0.02	-0.09	0.10	0.05	0.07	0.04

[a] Semi-experimental equilibrium geometry, from Ref. (Gambi et al., 2002)
[b] CCSD(T)/CBS + Δ(CV), from Ref. (Gambi et al., 2002)

Table S15. ClFC=CF_2 bond angles in degree obtained at the different levels of theory and comparison to the semi-experimental equilibrium geometry.

ClFC=CF_2	SE	CCSD(T)[a] ANO2	B3LYP-D3 SNSD	ωB97 jul-cc-pVDZ	ωB97 aug-cc-pVDZ	ωB97X jul-cc-pVDZ	ωB97X aug-cc-pVDZ	ωB97XD jul-cc-pVDZ	ωB97XD aug-cc-pVDZ
F1C1C2	n.a.	123.50	123.33	123.51	123.51	123.44	123.44	123.38	123.38
F2C1C2	n.a.	123.90	124.16	124.12	124.12	124.15	124.15	124.19	124.19
C1C2F3	n.a.	120.30	120.34	119.90	119.90	120.05	120.05	120.24	120.24
C1C2Cl	n.a.	123.90	124.31	124.63	124.63	124.51	124.51	124.37	124.37
MD			0.13	0.14	0.14	0.13	0.13	0.15	0.15
MAD			0.22	0.34	0.34	0.29	0.29	0.24	0.24
Max Pos			0.41	0.73	0.73	0.61	0.61	0.47	0.47
Max Neg			-0.17	-0.40	-0.40	-0.25	-0.25	-0.12	-0.12

ClFC=CF_2	PW6B95-D3 jul-cc-pVDZ	PW6B95-D3 aug-cc-pVDZ	PW6B95-D3 jun-cc-pVDZ	B2PLYP-D3 VTZ	B2PLYP-D3 augVTZ	B2PLYP-D3 may'VTZ	rev-DSD-PBEP86-D3 jun-cc-pVTZ	rev-DSD-PBEP86-D3 aug-cc-pVTZ
F1C1C2	123.42	123.42	123.51	123.37	123.41	123.39	123.43	123.44
F2C1C2	124.06	124.06	124.01	124.05	124.11	124.10	124.01	124.01
C1C2F3	120.01	120.01	120.07	120.36	120.21	120.10	120.16	120.19
C1C2Cl	124.36	124.36	124.29	123.96	124.16	124.20	124.03	124.03
MD	0.06	0.06	0.07	0.04	0.07	0.05	0.01	0.02
MAD	0.25	0.25	0.18	0.10	0.16	0.20	0.11	0.10
Max Pos	0.46	0.46	0.39	0.15	0.26	0.30	0.13	0.13
Max Neg	-0.29	-0.29	-0.23	-0.13	-0.09	-0.20	-0.14	-0.11

[a] CCSD(T)/ANOtz, from Ref. (Tasinato et al., 2012a)

Table S16. Oxirane bond angles in degree obtained at the different levels of theory and comparison to the semi-experimental equilibrium geometry.

Oxirane	SE[a]	CCSD(T)[b] CBS+CV	B3LYP-D3 SNSD (PES1)	ωB97 jul-cc-pVDZ	ωB97 aug-cc-pVDZ	ωB97X jul-cc-pVDZ	ωB97X aug-cc-pVDZ	ωB97XD jul-cc-pVDZ	ωB97XD aug-cc-pVDZ
COC	61.56	61.60	61.70	61.76	61.76	61.83	61.84	61.93	61.94
HCH	116.63	116.21	115.73	115.98	115.98	115.92	115.93	115.87	115.89
HCO	114.75	114.87	115.07	114.95	114.94	114.99	114.99	115.06	115.05
MD		-0.09	-0.15	-0.09	-0.08	-0.07	-0.06	-0.03	-0.02
MAD		0.19	0.45	0.35	0.35	0.41	0.40	0.48	0.47
Max Pos		0.12	0.32	0.20	0.20	0.27	0.28	0.37	0.38
Max Neg		-0.42	-0.90	-0.65	-0.65	-0.71	-0.70	-0.76	-0.74

Oxirane	PW6B95-D3 jul-cc-pVDZ	PW6B95-D3 aug-cc-pVDZ	PW6B95-D3 jun-cc-pVDZ	B2PYLP VTZ (PES5)	B2PLYP-D3 augVTZ (PES4)	B2PLYP-D3 may'VTZ (PES2)	rev-DSD-PBEP86-D3 jun-cc-pVTZ	rev-DSD-PBEP86-D3 aug-cc-pVTZ
COC	61.79	61.81	61.94	61.48	61.34	61.37	61.48	61.48
HCH	115.90	115.92	115.75	115.79	115.94	115.83	116.05	116.06
HCO	115.01	114.99	115.17	115.14	114.90	114.95	114.89	114.88
MD	-0.08	-0.07	-0.03	-0.18	-0.25	-0.27	-0.17	-0.18
MAD	0.41	0.40	0.56	0.44	0.35	0.40	0.26	0.26
Max Pos	0.26	0.25	0.42	0.39	0.15	0.20	0.14	0.13
Max Neg	-0.73	-0.71	-0.88	-0.84	-0.69	-0.80	-0.58	-0.57

[a] Semi-experimental equilibrium geometry, from Ref. (Puzzarini et al., 2014a)
[b] CCSD(T)/CBS + Δ(CV), from Ref. (Puzzarini et al., 2014a)

Table S17. CH_3CH_2SH bond angles in degree obtained at the different levels of theory and comparison to the semi-experimental equilibrium geometry.

CH_3CH_2SH	SE	CCSD(T)[a] CBS+CV	B3LYP-D3 SNSD	ωB97 jul-cc-pVDZ	ωB97 aug-cc-pVDZ	ωB97X jul-cc-pVDZ	ωB97X aug-cc-pVDZ	ωB97XD jul-cc-pVDZ	ωB97XD aug-cc-pVDZ
S3C1C2	n.a.	113.60	114.26	113.96	113.86	114.10	114.00	113.96	114.16
H4C1C2	n.a.	111.10	111.63	111.26	111.16	111.30	111.21	111.26	111.53
H5C1C2	n.a.	110.90	111.03	110.55	110.49	110.61	110.55	110.55	110.91
H6C2C1	n.a.	110.50	110.24	110.44	110.45	110.39	110.39	110.44	110.22
H7C2C1	n.a.	110.70	111.20	110.92	110.86	110.99	110.93	110.92	111.11
H8C2C1	n.a.	110.70	111.15	110.81	110.77	110.89	110.85	110.81	111.02
H9S3C1	n.a.	96.60	96.96	97.04	97.02	97.07	97.06	97.04	96.69
H4C1C2S3	n.a.	123.40	123.44	124.15	124.08	124.10	124.04	124.15	123.48
H5C1C2S3	n.a.	-117.20	-116.88	-117.39	-117.40	-117.31	-117.31	-117.39	-117.07
H6C2C1S3	n.a.	178	177.59	177.60	177.67	177.50	177.54	177.60	177.54
H7C2C1H6	n.a.	119.70	119.51	119.67	119.66	119.64	119.62	119.67	119.54
H8C1C2H6	n.a.	-120.50	-120.22	-120.42	-120.46	-120.37	-120.41	-120.42	-120.30
H9S3C1C2	n.a.	61.10	63.66	62.60	62.20	63.06	62.65	62.60	64.01
MD		Ref.	0.38	0.20	0.14	0.26	0.19	0.20	0.33
MAD		Ref.	0.52	0.36	0.29	0.42	0.36	0.36	0.47
Max Pos		Ref.	2.56	1.50	1.10	1.96	1.55	1.50	2.91
Max Neg		Ref.	-0.41	-0.40	-0.41	-0.50	-0.46	-0.40	-0.46

CH_3CH_2SH	PW6B95-D3 jul-cc-pVDZ	PW6B95-D3 aug-cc-pVDZ	PW6B95-D3 jun-cc-pVDZ	B2PLYP-D3 VTZ	B2PLYP-D3 augVTZ	B2PLYP-D3 may'VTZ	rev-DSD-PBEP86-D3 jun-cc-pVTZ	rev-DSD-PBEP86-D3 aug-cc-pVTZ
S3C1C2	113.61	113.98	114.36	113.98	113.95	114.06	113.61	113.56
H4C1C2	111.44	111.36	111.48	111.45	111.49	111.38	111.44	111.46
H5C1C2	110.83	110.72	110.88	110.87	110.92	110.83	110.83	110.87
H6C2C1	110.43	110.36	110.43	110.40	110.33	110.38	110.43	110.44
H7C2C1	110.83	110.93	111.15	110.98	110.97	111.00	110.83	110.79
H8C2C1	110.79	110.88	111.07	110.92	110.93	110.96	110.79	110.75
H9S3C1	96.61	96.69	96.70	96.69	96.80	96.81	96.61	96.60
H4C1C2S3	123.58	123.94	124.21	123.63	123.49	123.75	123.58	123.49
H5C1C2S3	-117.01	-117.01	-116.97	-116.97	-116.94	-117.00	-117.01	-116.97
H6C2C1S3	177.79	177.36	177.23	177.51	177.73	177.48	177.79	177.86
H7C2C1H6	119.67	119.59	119.52	119.62	119.62	119.60	119.67	119.70
H8C1C2H6	-120.45	-120.41	-120.39	-120.39	-120.35	-120.36	-120.45	-120.47
H9S3C1C2	61.78	62.29	62.12	62.62	62.58	61.92	61.78	61.78
MD	0.10	0.16	0.24	0.21	0.22	0.17	0.10	0.10
MAD	0.16	0.32	0.41	0.32	0.30	0.29	0.16	0.14
Max Pos	0.68	1.19	1.02	1.52	1.48	0.82	0.68	0.68
Max Neg	-0.21	-0.64	-0.77	-0.49	-0.27	-0.52	-0.21	-0.14

[a] CCSD(T)/CBS + Δ(CV), from Ref. (Hochlaf et al., 2015)

Table S18. 8. SO_2 bond angles in degree obtained at the different levels of theory and comparison to the semi-experimental equilibrium geometry.

SO_2	SE[a]	CCSD(T)[b] CBS+CV	B3LYP-D3 SNSD	ωB97 jul-cc-pVDZ	ωB97 aug-cc-pVDZ	ωB97X jul-cc-pVDZ	ωB97X aug-cc-pVDZ	ωB97XD jul-cc-pVDZ	ωB97XD aug-cc-pVDZ
OSO	119.31	119.13	117.32	118.84	118.84	118.90	118.90	119.02	119.02

SO_2	PW6B95-D3 jul-cc-pVDZ	PW6B95-D3 aug-cc-pVDZ	PW6B95-D3 jun-cc-pVDZ	B2PLYP-D3 VTZ	B2PLYP-D3 augVTZ	B2PLYP-D3 may'VTZ	rev-DSD-PBEP86-D3 jun-cc-pVTZ	rev-DSD-PBEP86-D3 aug-cc-pVTZ
OSO	119.08	118.35	119.20	118.52	118.35	119.35	119.28	119.24

[a] Semi-experimental equilibrium geometry, from Ref. (Boussessi et al., 2020)
[b] CCSD(T)/CBS + Δ(CV), from Ref. (Boussessi et al., 2020)

Table S19. $HCOCH_2OH$ bond angles in degree obtained at the different levels of theory and comparison to the semi-experimental equilibrium geometry.

$HCOCH_2OH$	SE[a]	Cheap[b]	B3LYP-D3 SNSD	ωB97 jul-cc-pVDZ	ωB97 aug-cc-pVDZ	ωB97X jul-cc-pVDZ	ωB97X aug-cc-pVDZ	ωB97XD jul-cc-pVDZ	ωB97XD aug-cc-pVDZ
C2C1O1	121.64	121.71	121.96	121.78	121.77	121.79	121.78	121.89	121.87
C2C1H	116.90	116.79	116.52	116.89	116.86	116.85	116.81	116.64	116.61
C1C2H4	107.91	107.84	107.75	107.71	107.78	107.71	107.78	107.61	107.66
C1C2O2	111.78	111.78	112.56	112.04	111.98	112.15	112.08	112.39	112.33
C2OH	106.18	106.22	106.53	106.40	106.43	106.49	106.52	106.50	106.52
H4C2C1O1	122.34	122.45	122.83	122.58	122.50	122.67	122.59	122.81	122.73
MD		0.01	0.23	0.11	0.09	0.15	0.13	0.18	0.16
MAD		0.07	0.41	0.18	0.15	0.24	0.21	0.37	0.34
Max Pos		0.11	0.78	0.26	0.25	0.37	0.34	0.61	0.55
Max Neg		-0.11	-0.38	-0.20	-0.13	-0.20	-0.13	-0.30	-0.29

$HCOCH_2OH$	PW6B95-D3 jul-cc-pVDZ	PW6B95-D3 aug-cc-pVDZ	PW6B95-D3 jun-cc-pVDZ	B2PLYP-D3 VTZ	B2PLYP-D3 augVTZ	B2PLYP-D3 may'VTZ	rev-DSD-PBEP86-D3 jun-cc-pVTZ	rev-DSD-PBEP86-D3 aug-cc-pVTZ
C2C1O1	121.58	121.56	121.59	121.88	122.02	122.02	121.99	122.01
C2C1H	116.94	116.92	116.88	116.54	116.46	116.46	116.54	116.51
C1C2H4	107.77	107.84	107.62	107.65	107.83	107.82	107.85	107.84
C1C2O2	112.03	111.98	112.28	112.10	112.28	112.32	112.08	112.12
C2OH	106.16	106.19	105.87	105.76	106.38	106.35	106.05	106.13
H4C2C1O1	122.80	122.72	122.98	122.79	122.61	122.63	122.52	122.50
MD	0.09	0.08	0.08	-0.00	0.14	0.14	0.05	0.06
MAD	0.16	0.13	0.30	0.34	0.31	0.32	0.23	0.23
Max Pos	0.46	0.38	0.64	0.45	0.50	0.54	0.35	0.37
Max Neg	-0.14	-0.08	-0.31	-0.42	-0.44	-0.44	-0.36	-0.39

[a] Semi-experimental equilibrium geometry, from Ref. (Piccardo et al., 2015)
[b] Cheap composite scheme, from Ref. (Boussessi et al., 2020)

Table S20. *E*-Ethanimine bond angles in degree obtained at the different levels of theory and comparison to the semi-experimental equilibrium geometry.

E-Ethanimine	SE	CCSD(T)[a] CBS+CV	B3LYP-D3 SNSD	ωB97 jul-cc-pVDZ	ωB97 aug-cc-pVDZ	ωB97X jul-cc-pVDZ	ωB97X aug-cc-pVDZ	ωB97XD jul-cc-pVDZ	ωB97XD aug-cc-pVDZ
N3C2C1	n.a.	121.37	121.84	121.61	121.59	121.66	121.65	121.73	121.72
H4N3C2	n.a.	110.33	110.73	110.08	110.17	110.34	110.42	110.48	110.55
H5C1C2	n.a.	116.40	115.79	116.25	116.25	116.17	116.17	115.94	115.94
H6C1C2	n.a.	110.28	110.29	110.12	110.16	110.16	110.20	110.10	110.12
H8C1C2	n.a.	110.02	110.47	110.02	109.91	110.13	110.02	110.39	110.25
H6C1C2N3	n.a.	-120.97	-121.19	-121.04	-121.01	-121.07	-121.04	-121.17	-121.14
MD		Ref.	0.08	-0.06	-0.06	-0.01	-0.00	0.01	0.00
MAD			0.36	0.14	0.13	0.14	0.13	0.29	0.26
Max Pos			0.47	0.24	0.22	0.29	0.28	0.37	0.35
Max Neg			-0.61	-0.25	-0.16	-0.23	-0.23	-0.46	-0.46

E-Ethanimine	PW6B95-D3 jul-cc-pVDZ	PW6B95-D3 aug-cc-pVDZ	PW6B95-D3 jun-cc-pVDZ	B2PLYP-D3 VTZ	B2PLYP-D3 augVTZ	B2PLYP-D3 may'VTZ	rev-DSD-PBEP86-D3 jun-cc-pVTZ	rev-DSD-PBEP86-D3 aug-cc-pVTZ
N3C2C1	121.64	121.63	121.80	121.57	121.70	121.70	121.56	121.52
H4N3C2	110.55	110.62	110.36	110.46	110.87	110.76	110.34	110.43
H5C1C2	115.99	116.00	115.74	115.77	115.94	115.90	116.10	116.11
H6C1C2	110.21	110.26	110.26	110.37	110.23	110.26	110.22	110.20
H8C1C2	110.25	110.14	110.50	110.07	110.31	110.32	110.19	110.17
H6C1C2N3	-121.18	-121.15	-121.21	-121.01	-121.12	-121.10	-121.05	-121.04
MD	0.01	0.01	0.00	-0.03	0.08	0.07	-0.01	-0.01
MAD	0.23	0.21	0.31	0.19	0.30	0.29	0.14	0.14
Max Pos	0.27	0.29	0.48	0.20	0.54	0.43	0.19	0.15
Max Neg	-0.41	-0.40	-0.66	-0.63	-0.46	-0.50	-0.30	-0.29

[a] CCSD(T)/CBS + Δ(CV), from Ref. (Boussessi et al., 2020)

1.4 Rotational Constants

Table S21. CH_2F_2 rotational constants in cm^{-1} obtained at the different levels of theory and comparison to the experimental results.

	Exp[a]	CCSD(T)[b]	B3LYP-D3	ωB97		ωB97X		ωB97XD	
CH_2F_2	aug-cc-pCVQZ	SNSD	jul-cc-pVDZ	aug-cc-pVDZ	jul-cc-pVDZ	aug-cc-pVDZ	jul-cc-pVDZ	aug-cc-pVDZ	
A	1.63923	1.65558	1.61687	1.59384	1.59375	1.60543	1.63514	1.61241	1.61256
B	0.35374	0.35541	0.34628	0.34492	0.34513	0.34722	0.34986	0.34801	0.34825
C	0.30854	0.31053	0.30233	0.30081	0.30096	0.30288	0.30750	0.30369	0.30388
MD%		0.71	-1.83	-2.59	-2.56	-1.91	-0.56	-1.61	-1.56
MAD%		0.71	1.83	2.59	2.56	1.91	0.56	1.61	1.56
Max Pos		1.00	-1.36	-2.49	-2.43	-1.84	-0.25	-1.57	-1.51
Max Neg		0.47	-2.11	-2.77	-2.77	-2.06	-1.10	-1.64	-1.63

	PW6B95-D3			B2PLYP			rev-DSD-PBEP86-D3	
CH_2F_2	jul-cc-pVDZ	aug-cc-pVDZ	jun-cc-pVDZ	VTZ	augVTZ	may'VTZ	jun-cc-pVTZ	aug-cc-pVTZ
A	1.61719	1.61757	1.62068	1.63965	1.62797	1.63127	1.62958	1.62764
B	0.34956	0.34983	0.35085	0.35009	0.34935	0.34943	0.35076	0.35059
C	0.30489	0.30510	0.30603	0.30572	0.30483	0.30500	0.30602	0.30585
MD%	-1.24	-1.18	-0.92	-0.64	-1.04	-0.95	-0.75	-0.82
MAD%	1.24	1.18	0.92	0.66	1.04	0.95	0.75	0.82
Max Pos	-1.18	-1.11	-0.81	0.03	-0.69	-0.49	-0.59	-0.71
Max Neg	-1.34	-1.32	-1.13	-1.03	-1.24	-1.22	-0.84	-0.89

[a] From Ref. (Carlotti et al., 1988)
[b] Geometry and quadratic force constants obtained at the CCSD(T)/aug-cc-pCVQZ level; cubic and semi-diagonal force constants evaluated at the CCSD(T) level employing the aug-cc-pCVQZ basis set for F atoms and the aug-cc-pCVTZ basis for H and C atoms. From Ref. (Tasinato et al., 2012b).

Table S22. CH_2ClF rotational constants in cm^{-1} obtained at the different levels of theory and comparison to the experimental results.

CH_2ClF	Exp[a]	CCSD(T)[b] CBS+CV+aug	B3LYP-D3 SNSD	ωB97 jul-cc-pVDZ	ωB97 aug-cc-pVDZ	ωB97X jul-cc-pVDZ	ωB97X aug-cc-pVDZ	ωB97XD jul-cc-pVDZ	ωB97XD aug-cc-pVDZ
A	1.39467	1.38971	1.37475	1.36552	1.36567	1.37195	1.37224	1.37528	1.37574
B	0.19066	0.19095	0.18472	0.19042	0.19043	0.18984	0.18985	0.18840	0.18840
C	0.17328	0.17346	0.16815	0.17277	0.17278	0.17237	0.17238	0.17125	0.17125
MD%		-0.03	-2.50	-0.84	-0.83	-0.86	-0.85	-1.25	-1.24
MAD%		0.20	2.50	0.84	0.83	0.86	0.85	1.25	1.24
Max Pos		0.15	-1.43	-0.13	-0.12	-0.43	-0.43	-1.17	-1.17
Max Neg		-0.36	-3.12	-2.09	-2.08	-1.63	-1.61	-1.39	-1.36

CH_2ClF	PW6B95-D3 jul-cc-pVDZ	PW6B95-D3 aug-cc-pVDZ	PW6B95-D3 jun-cc-pVDZ	B2PLYP VTZ	B2PLYP augVTZ	B2PLYP may'VTZ	rev-DSD-PBEP86-D3 jun-cc-pVTZ	rev-DSD-PBEP86-D3 aug-cc-pVTZ
A	1.38063	1.38112	1.38606	1.39525	1.38676	1.39042	1.38847	1.38728
B	0.18967	0.18967	0.18970	0.18723	0.18743	0.18793	0.18886	0.18889
C	0.17233	0.17233	0.17244	0.17045	0.17050	0.17095	0.17172	0.17174
MD%	-0.69	-0.68	-0.54	-1.13	-1.29	-1.03	-0.76	-0.78
MAD%	0.69	0.68	0.54	1.16	1.29	1.03	0.76	0.78
Max Pos	-0.52	-0.52	-0.48	0.04	-0.57	-0.30	-0.44	-0.53
Max Neg	-1.01	-0.97	-0.62	-1.80	-1.70	-1.44	-0.94	-0.93

[a] From Ref. (Blanco et al., 1995)
[b] CCSD(T)/CBS + Δ (CV) + Δ(aug) augmented through vibrational corrections at the a.e.-CCSD(T)/cc-pCVTZ level. From Ref. (Pietropolli Charmet et al., 2013).

Table S23. *cis*-ClHC=CHF rotational constants in cm^{-1} obtained at the different levels of theory and comparison to the experimental results.

	Exp[a]	CCSD(T)[b]	B3LYP-D3	ωB97		ωB97X		ωB97XD	
cis-ClHC=CHF		scaled + ΔB_{vib}	SNSD	jul-cc-pVDZ	aug-cc-pVDZ	jul-cc-pVDZ	aug-cc-pVDZ	jul-cc-pVDZ	aug-cc-pVDZ
A	0.54723	0.48187	0.54334	0.54139	0.54158	0.54416	0.54439	0.54757	0.54773
B	0.12530	0.12257	0.12135	0.12425	0.12427	0.12394	0.12396	0.12260	0.12264
C	0.10183	0.10007	0.09907	0.10094	0.10096	0.10083	0.10085	0.10005	0.10009
MD%		-5.28	-2.19	-0.93	-0.91	-0.88	-0.85	-1.28	-1.25
MAD%		5.28	2.19	0.93	0.91	0.88	0.85	1.32	1.31
Max Pos		-1.73	-0.71	-0.84	-0.82	-0.56	-0.52	0.06	0.09
Max Neg		-11.95	-3.16	-1.07	-1.03	-1.08	-1.07	-2.16	-2.12

	PW6B95-D3			B2PLYP			rev-DSD-PBEP86-D3	
cis-ClHC=CHF	jul-cc-pVDZ	aug-cc-pVDZ	jun-cc-pVDZ	VTZ	augVTZ	may'VTZ	jun-cc-pVTZ	aug-cc-pVTZ
A	0.54743	0.54762	0.54898	0.54860	0.54708	0.54927	0.54776	0.54717
B	0.12414	0.12417	0.12397	0.12307	0.12319	0.12323	0.12372	0.12382
C	0.10107	0.10109	0.10100	0.10040	0.10043	0.10053	0.10080	0.10085
MD%	-0.55	-0.52	-0.52	-0.98	-1.03	-0.85	-0.73	-0.72
MAD%	0.57	0.57	0.73	1.15	1.03	1.10	0.79	0.72
Max Pos	0.04	0.07	0.32	0.25	-0.03	0.37	0.10	-0.01
Max Neg	-0.93	-0.90	-1.07	-1.78	-1.68	-1.65	-1.26	-1.19

[a] From Ref. (Alonso et al., 1993)
[b] Scaled CCSD(T). From Ref. (Gambi et al., 2002)

Table S24. ClFC=CH_2 rotational constants in cm^{-1} obtained at the different levels of theory and comparison to the experimental results.

ClFC=CH_2	Exp[a]	CCSD(T)[b] CVTZ+CVTZ(F)	B3LYP-D3 SNSD	ωB97 jul-cc-pVDZ	ωB97 aug-cc-pVDZ	ωB97X jul-cc-pVDZ	ωB97X aug-cc-pVDZ	ωB97XD jul-cc-pVDZ	ωB97XD aug-cc-pVDZ
A	0.35630	0.35475	0.35225	0.35140	0.35146	0.35301	0.35308	0.35321	0.35329
B	0.17019	0.16893	0.16606	0.16959	0.16958	0.16936	0.16935	0.16865	0.16864
C	0.11502	0.11428	0.11271	0.11423	0.11424	0.11430	0.11430	0.11400	0.11400
MD%		-0.60	-1.86	-0.81	-0.80	-0.68	-0.68	-0.89	-0.88
MAD%		0.60	1.86	0.81	0.80	0.68	0.68	0.89	0.88
Max Pos		-0.43	-1.14	-0.35	-0.36	-0.49	-0.49	-0.87	-0.84
Max Neg		-0.74	-2.43	-1.38	-1.36	-0.92	-0.90	-0.90	-0.91

ClFC=CH_2	PW6B95-D3 jul-cc-pVDZ	PW6B95-D3 aug-cc-pVDZ	PW6B95-D3 jun-cc-pVDZ	B2PLYP VTZ	B2PLYP augVTZ	B2PLYP may'VTZ	rev-DSD-PBEP86-D3 jun-cc-pVTZ	rev-DSD-PBEP86-D3 aug-cc-pVTZ
A	0.35437	0.35446	0.35457	0.35509	0.35478	0.35507	0.35468	0.35450
B	0.16981	0.16979	0.16983	0.16839	0.16833	0.16885	0.16911	0.16915
C	0.11464	0.11464	0.11468	0.11407	0.11401	0.11428	0.11436	0.11436
MD%	-0.37	-0.36	-0.33	-0.74	-0.80	-0.59	-0.56	-0.57
MAD%	0.37	0.36	0.33	0.74	0.80	0.59	0.56	0.57
Max Pos	-0.22	-0.23	-0.21	-0.34	-0.43	-0.35	-0.45	-0.51
Max Neg	-0.54	-0.52	-0.49	-1.06	-1.09	-0.79	-0.64	-0.61

[a] From Ref. (Leung et al., 2009)
[b] a.e.-CCSD(T)/cc-p CVTZ/aug-cc-pCVTZ(F). From Ref. (Pietropolli Charmet et al., 2016)

Table S25. $ClFC{=}CF_2$ rotational constants in cm^{-1} obtained at the different levels of theory and comparison to the experimental results.

$ClFC{=}CF_2$	Exp[a]	CCSD(T)[b] ANO2	B3LYP-D3 SNSD	ωB97 jul-cc-pVDZ	ωB97 aug-cc-pVDZ	ωB97X jul-cc-pVDZ	ωB97X aug-cc-pVDZ	ωB97XD jul-cc-pVDZ	ωB97XD aug-cc-pVDZ
A	0.15031	0.14973	0.14840	0.14826	0.14826	0.14906	0.14906	0.14938	0.14938
B	0.07568	0.07528	0.07399	0.07479	0.07479	0.07490	0.07490	0.07474	0.07474
C	0.05030	0.05010	0.04934	0.04968	0.04968	0.04982	0.04982	0.04979	0.04979
MD%		-0.44	-1.80	-1.26	-1.26	-0.94	-0.94	-0.96	-0.96
MAD%		0.44	1.80	1.26	1.26	0.94	0.94	0.96	0.96
Max Pos		-0.38	-1.27	-1.18	-1.18	-0.83	-0.83	-0.62	-0.62
Max Neg		-0.52	-2.23	-1.36	-1.36	-1.02	-1.02	-1.24	-1.24

$ClFC{=}CF_2$	PW6B95-D3 jul-cc-pVDZ	PW6B95-D3 aug-cc-pVDZ	PW6B95-D3 jun-cc-pVDZ	B2PLYP VTZ	B2PLYP augVTZ	B2PLYP may'VTZ	rev-DSD-PBEP86-D3 jun-cc-pVTZ	rev-DSD-PBEP86-D3 aug-cc-pVTZ
A	0.14968	0.14968	0.14994	0.14928	0.14932	0.14948	0.14954	0.14947
B	0.07524	0.07524	0.07526	0.07499	0.07490	0.07505	0.07515	0.07516
C	0.05004	0.05004	0.05008	0.04988	0.04985	0.04994	0.04998	0.04998
MD%	-0.50	-0.50	-0.41	-0.81	-0.86	-0.70	-0.62	-0.63
MAD%	0.50	0.50	0.41	0.81	0.86	0.70	0.62	0.63
Max Pos	-0.42	-0.42	-0.25	-0.68	-0.66	-0.55	-0.51	-0.56
Max Neg	-0.57	-0.57	-0.54	-0.91	-1.03	-0.82	-0.70	-0.68

[a] From Ref. (Hillig et al., 1988).

[b] b Geometry and harmonic force field at CCSD(T)/ANOtz level; cubic and quartic force constants at CCSD(T)/cc-pVTZ level. From Ref. (Tasinato et al., 2012a).

Table S26. Oxirane rotational constants in cm^{-1} obtained at the different levels of theory and comparison to the experimental results.

Oxirane	Exp[a]	CCSD(T)[b] CBS+CV+aug+ΔB_{vib}	B3LYP-D3 SNSD (PES1)	ωB97 jul-cc-pVDZ	ωB97 aug-cc-pVDZ	ωB97X jul-cc-pVDZ	ωB97X aug-cc-pVDZ	ωB97XD jul-cc-pVDZ	ωB97XD aug-cc-pVDZ
A	0.85005	0.85153	0.84605	0.84931	0.84969	0.85210	0.85250	0.85244	0.85290
B	0.73787	0.73788	0.72947	0.73050	0.73083	0.73102	0.73136	0.72896	0.72929
C	0.47025	0.47023	0.46630	0.46893	0.46903	0.46970	0.46982	0.46895	0.46909
MD%		0.06	-0.82	-0.46	-0.42	-0.27	-0.23	-0.40	-0.36
MAD%		0.06	0.82	0.46	0.42	0.43	0.42	0.59	0.58
Max Pos		0.17	-0.47	-0.09	-0.04	0.24	0.29	0.28	0.34
Max Neg		-0.01	-1.14	-1.00	-0.95	-0.93	-0.88	-1.21	-1.16

Oxirane	PW6B95-D3 jul-cc-pVDZ	PW6B95-D3 aug-cc-pVDZ	PW6B95-D3 jun-cc-pVDZ	B2PYLP VTZ (PES5)	B2PLYP augVTZ (PES4)	B2PLYP may'VTZ (PES2)	rev-DSD-PBEP86-D3 jun-cc-pVTZ	rev-DSD-PBEP86-D3 aug-cc-pVTZ
A	0.85368	0.85424	0.85709	0.84663	0.84371	0.84435	0.84511	0.84479
B	0.73362	0.73393	0.73321	0.73672	0.73639	0.73622	0.73446	0.73425
C	0.47049	0.47066	0.47157	0.46838	0.46745	0.46750	0.46762	0.46749
MD%	-0.03	0.01	0.16	-0.32	-0.51	-0.49	-0.53	-0.57
MAD%	0.35	0.37	0.58	0.32	0.51	0.49	0.53	0.57
Max Pos	0.43	0.49	0.83	-0.16	-0.20	-0.22	-0.46	-0.49
Max Neg	-0.58	-0.53	-0.63	-0.40	-0.75	-0.67	-0.58	-0.62

[a] From Ref. (Medcraft et al., 2012).

[b] CCSD(T)/CBS + Δ(CV) geometry, vibrational corrections at CCSD(T)/cc-pVQZ and electronic corrections at CCSD(T)/aug-cc-pVQZ.. From Ref. (Puzzarini et al., 2014a)

Table S27. CH_3CH_2SH rotational constants in cm^{-1} obtained at the different levels of theory and comparison to the experimental results.

CH_3CH_2SH	Exp[a]	CCSD(T)[b] CBS+CV+ΔB_{vib}	B3LYP-D3 SNSD	ωB97 jul-cc-pVDZ	ωB97 aug-cc-pVDZ	ωB97X jul-cc-pVDZ	ωB97X aug-cc-pVDZ	ωB97XD jul-cc-pVDZ	ωB97XD aug-cc-pVDZ
A	0.95891	0.95984	0.95298	0.95600	0.95635	0.95505	0.95638	0.95718	0.95236
B	0.17663	0.17675	0.17009	0.17549	0.17546	0.17551	0.17505	0.17448	0.17264
C	0.16164	0.16164	0.15641	0.16116	0.16118	0.16053	0.16057	0.16103	0.15770
MD%		0.05	-2.52	-0.42	-0.40	-0.58	-0.61	-0.59	-1.79
MAD%		0.06	2.52	0.42	0.40	0.58	0.61	0.59	1.79
Max Pos		0.10	-0.62	-0.30	-0.27	-0.40	-0.26	-0.18	-0.68
Max Neg		-0.00	-3.70	-0.64	-0.66	-0.69	-0.89	-1.22	-2.44

CH_3CH_2SH	PW6B95-D3 jul-cc-pVDZ	PW6B95-D3 aug-cc-pVDZ	PW6B95-D3 jun-cc-pVDZ	B2PLYP VTZ	B2PLYP augVTZ	B2PLYP may'VTZ	rev-DSD-PBEP86-D3 jun-cc-pVTZ	rev-DSD-PBEP86-D3 aug-cc-pVTZ
A	0.95820	0.95774	0.95991	0.96047	0.95976	0.96219	0.95505	0.95429
B	0.17578	0.17583	0.17538	0.17357	0.17357	0.17399	0.17517	0.17528
C	0.16075	0.16081	0.16061	0.15921	0.15919	0.15954	0.16036	0.16045
MD%	-0.37	-0.36	-0.41	-1.03	-1.05	-0.82	-0.67	-0.66
MAD%	0.37	0.36	0.48	1.13	1.11	1.05	0.67	0.66
Max Pos	-0.07	-0.12	0.10	0.16	0.09	0.34	-0.40	-0.48
Max Neg	-0.56	-0.52	-0.70	-1.73	-1.73	-1.49	-0.83	-0.76

[a] From Ref. (Kolesniková et al., 2014)
[b] CCSD(T)/CBS + Δ(CV) geometry, vibrational corrections at CCSD(T)/cc-pVTZ level. From Ref. (Puzzarini et al., 2014b).

Table S28. SO_2 rotational constants in cm^{-1} obtained at the different levels of theory and comparison to the experimental results.

SO_2	Exp[a]	CCSD(T)[b] CBS+CV+ΔB_{vib}	B3LYP-D3 SNSD	ωB97 jul-cc-pVDZ	ωB97 aug-cc-pVDZ	ωB97X jul-cc-pVDZ	ωB97X aug-cc-pVDZ	ωB97XD jul-cc-pVDZ	ωB97XD aug-cc-pVDZ
A	2.02735	2.01366	1.78136	1.96389	1.96389	1.97050	1.97050	1.97591	1.97591
B	0.34417	0.34467	0.32759	0.33999	0.33999	0.34028	0.34028	0.33940	0.33940
C	0.29353	0.29364	0.27604	0.28913	0.28913	0.28948	0.28948	0.28896	0.28896
MD%		-0.16	-7.64	-1.95	-1.95	-1.77	-1.77	-1.83	-1.83
MAD%		0.29	7.64	1.95	1.95	1.77	1.77	1.83	1.83
Max Pos		0.15	-4.82	-1.22	-1.22	-1.13	-1.13	-1.39	-1.39
Max Neg		-0.68	-12.13	-3.13	-3.13	-2.80	-2.80	-2.54	-2.54

SO_2	PW6B95-D3 jul-cc-pVDZ	PW6B95-D3 aug-cc-pVDZ	PW6B95-D3 jun-cc-pVDZ	B2PLYP VTZ	B2PLYP augVTZ	B2PLYP may'VTZ	rev-DSD-PBEP86-D3 jun-cc-pVTZ	rev-DSD-PBEP86-D3 aug-cc-pVTZ
A	1.97319	1.89596	1.98861	1.91078	1.89634	1.99301	1.99339	1.98980
B	0.33824	0.33487	0.33937	0.33495	0.33463	0.33790	0.33896	0.33881
C	0.28805	0.28393	0.28921	0.28431	0.28375	0.28822	0.28901	0.28882
MD%	-2.09	-4.15	-1.59	-3.86	-4.19	-1.78	-1.58	-1.67
MAD%	2.09	4.15	1.59	3.86	4.19	1.78	1.58	1.67
Max Pos	-1.73	-2.70	-1.40	-2.68	-2.77	-1.69	-1.51	-1.56
Max Neg	-2.67	-6.48	-1.91	-5.75	-6.46	-1.82	-1.68	-1.85

[a] From Ref. (Müller and Brünken, 2005)
[b] CCSD(T)/CBS + Δ(CV) equilibrium geometry, vibrational corrections at B2PLYP/cc-pVTZ level. From Ref. (Boussessi et al., 2020).

Table S29. $HCOCH_2OH$ rotational constants in cm^{-1} obtained at the different levels of theory and comparison to the experimental results.

	Ex[a]	Cheap[b]	B3LYP-D3	ωB97		ωB97X		ωB97XD	
$HCOCH_2OH$		$+\Delta B_{vib}$	SNSD	jul-cc-pVDZ	aug-cc-pVDZ	jul-cc-pVDZ	aug-cc-pVDZ	jul-cc-pVDZ	aug-cc-pVDZ
A	0.61530	0.61056	0.61459	0.60906	0.60921	0.61009	0.61031	0.61263	0.61101
B	0.21768	0.21705	0.21352	0.21696	0.21693	0.21894	0.21890	0.21784	0.21921
C	0.16576	0.16500	0.16329	0.16497	0.16496	0.16617	0.16616	0.16572	0.16639
MD%		-0.51	-1.17	-0.61	-0.61	-0.01	-0.00	-0.13	0.13
MAD%		0.51	1.17	0.61	0.61	0.56	0.54	0.18	0.59
Max Pos		-0.29	-0.12	-0.33	-0.34	0.58	0.56	0.07	0.70
Max Neg		-0.77	-1.91	-1.01	-0.99	-0.85	-0.81	-0.43	-0.70

	PW6B95-D3			B2PLYP			rev-DSD-PBEP86-D3	
$HCOCH_2OH$	jul-cc-pVDZ	aug-cc-pVDZ	jun-cc-pVDZ	VTZ	augVTZ	may'VTZ	jun-cc-pVTZ	aug-cc-pVTZ
A	0.61640	0.61644	0.61733	0.61547	0.61559	0.61594	0.61437	0.61431
B	0.21768	0.21781	0.21738	0.21591	0.21491	0.21470	0.21476	0.21472
C	0.16584	0.16592	0.16575	0.16470	0.16413	0.16404	0.16400	0.16397
MD%	0.08	0.11	0.06	-0.47	-0.74	-0.77	-0.85	-0.87
MAD%	0.08	0.11	0.16	0.49	0.77	0.84	0.85	0.87
Max Pos	0.18	0.18	0.33	0.03	0.05	0.10	-0.15	-0.16
Max Neg	0.00	0.06	-0.14	-0.82	-1.27	-1.37	-1.34	-1.36

[a] From Ref. (Carroll et al., 2010)
[b] Cheap equilibrium geometry geometry, vibrational corrections at B2PLYP/cc-pVTZ level. From Ref. (Boussessi et al., 2020).

Table S30. *E*-Ethanimine rotational constants in cm^{-1} obtained at the different levels of theory and comparison to the experimental results.

	Exp[a]	CCSD(T)[b]	B3LYP-D3	ωB97		ωB97X		ωB97XD	
E-Ethanimine		CBS+CV+ΔB_{vib}	SNSD	jul-cc-pVDZ	aug-cc-pVDZ	jul-cc-pVDZ	aug-cc-pVDZ	jul-cc-pVDZ	aug-cc-pVDZ
A	1.77191	1.77384	1.77108	1.75803	1.75885	1.76382	1.76474	1.76335	1.76419
B	0.32632	0.32623	0.32266	0.32323	0.32333	0.32389	0.32400	0.32345	0.32358
C	0.29010	0.29029	0.28752	0.28781	0.28790	0.28846	0.28855	0.28810	0.28821
MD%		0.05	-0.69	-0.84	-0.80	-0.59	-0.55	-0.68	-0.64
MAD%		0.07	0.69	0.84	0.80	0.59	0.55	0.68	0.64
Max Pos		0.11	-0.05	-0.78	-0.74	-0.46	-0.40	-0.48	-0.44
Max Neg		-0.03	-1.12	-0.95	-0.92	-0.74	-0.71	-0.88	-0.84

	PW6B95-D3			B2PLYP			rev-DSD-PBEP86-D3	
E-Ethanimine	jul-cc-pVDZ	aug-cc-pVDZ	jun-cc-pVDZ	VTZ	augVTZ	may'VTZ	jun-cc-pVTZ	aug-cc-pVTZ
A	1.77464	1.77581	1.77307	1.77907	1.78205	1.78189	1.76997	1.76894
B	0.32588	0.32598	0.32540	0.32543	0.32495	0.32491	0.32423	0.32430
C	0.29014	0.29023	0.28977	0.28977	0.28947	0.28944	0.28867	0.28871
MD%	0.01	0.05	-0.11	0.01	-0.02	-0.03	-0.41	-0.42
MAD%	0.10	0.12	0.15	0.26	0.40	0.41	0.41	0.42
Max Pos	0.15	0.22	0.07	0.40	0.57	0.56	-0.11	-0.17
Max Neg	-0.14	-0.10	-0.28	-0.27	-0.42	-0.43	-0.64	-0.62

[a] From Ref. (Melli et al., 2018)
[b] CCSD(T)/CBS + Δ(CV) geometry, vibrational corrections at B2PLYP/maug-cc-pVTZ-dH level. From Ref. (Melli et al., 2018).

1.5 Quartic Centrifugal Distortion Constants

Table S31. CH_2F_2 Quartic Centrifugal Distortion Constants (in cm^{-1}) obtained at the different levels of theory and comparison to the experimental results.

	Exp[a]	CCSD(T)[b]	B3LYP-D3	ωB97		ωB97X		ωB97XD	
CH_2F_2		aug-cc-pCVQZ	SNSD	jul-cc-pVDZ	aug-cc-pVDZ	jul-cc-pVDZ	aug-cc-pVDZ	jul-cc-pVDZ	aug-cc-pVDZ
Δ_J	3.725E-07	3.692E-07	3.701E-07	3.694E-07	3.699E-07	3.701E-07	3.706E-07	3.721E-07	3.726E-07
Δ_{JK}	-2.092E-06	-2.109E-06	-2.129E-06	-2.206E-06	-2.204E-06	-2.184E-06	-2.182E-06	-2.166E-06	-2.163E-06
Δ_K	2.091E-05	2.102E-05	2.143E-05	2.092E-05	2.090E-05	2.095E-05	2.093E-05	2.106E-05	2.104E-05
δ_J	7.177E-08	7.080E-08	7.069E-08	7.175E-08	7.189E-08	7.163E-08	7.177E-08	7.164E-08	7.176E-08
δ_K	9.393E-07	8.961E-07	9.166E-07	8.913E-07	8.926E-07	8.953E-07	8.965E-07	9.049E-07	9.058E-07
MD%		-1.1029	-0.0629	-0.1004	-0.0412	-0.1944	-0.1392	0.0527	0.0864
MAD%		1.6322	1.7663	2.2913	2.2412	2.0161	1.8916	1.6312	1.5200
Max Pos		0.7992	2.4880	5.4458	5.3356	4.3772	4.2798	3.4999	3.3790
Max Neg		-4.6002	-2.4175	-5.1104	-4.9705	-4.6864	-4.5615	-3.6666	-3.5628

	PW6B95-D3			B2PLYP-D3			rev-DSD-PBEP86-D3	
CH_2F_2	jul-cc-pVDZ	aug-cc-pVDZ	jun-cc-pVDZ	VTZ	augVTZ	may'VTZ	jun-cc-pVTZ	aug-cc-pVTZ
Δ_J	3.768E-07	3.772E-07	3.693E-07	3.651E-07	3.702E-07	3.676E-07	3.666E-07	3.679E-07
Δ_{JK}	-2.234E-06	-2.232E-06	-2.114E-06	-2.113E-06	-2.104E-06	-2.093E-06	-2.090E-06	-2.099E-06
Δ_K	2.141E-05	2.139E-05	2.095E-05	2.133E-05	2.125E-05	2.125E-05	2.094E-05	2.095E-05
δ_J	7.300E-08	7.313E-08	7.109E-08	6.960E-08	7.078E-08	7.017E-08	7.019E-08	7.049E-08
δ_K	9.128E-07	9.138E-07	8.958E-07	9.000E-07	9.141E-07	9.096E-07	8.961E-07	8.977E-07
MD%	1.8399	1.8796	-1.0475	-1.2544	-0.5078	-1.0187	-1.6728	-1.3887
MAD%	2.9692	2.9655	1.5259	2.4265	1.3636	1.6619	1.7238	1.5930
Max Pos	6.7569	6.6739	1.0208	1.9663	1.5922	1.5865	0.1276	0.3328
Max Neg	-2.8233	-2.7148	-4.6332	-4.1870	-2.6873	-3.1587	-4.5976	-4.4350

[a] From Ref. (Carlotti et al., 1988)
[b] a.e.-CCSD(T)/aug-cc-pCVQZ level. From Ref. (Tasinato et al., 2012b).

Table S32. CH_2FCl Quartic Centrifugal Distortion Constants (in cm^{-1}) obtained at the different levels of theory and comparison to the experimental results.

CH_2FCl	Exp[a]	CCSD(T)[b] aug-cc-pCVQZ	B3LYP-D3 SNSD	ωB97 jul-cc-pVDZ	ωB97 aug-cc-pVDZ	ωB97X jul-cc-pVDZ	ωB97X aug-cc-pVDZ	ωB97XD jul-cc-pVDZ	ωB97XD aug-cc-pVDZ
Δ_J	1.233E-07	1.224E-07	1.207E-07	1.165E-07	1.168E-07	1.178E-07	1.181E-07	1.196E-07	1.199E-07
Δ_{JK}	-1.174E-06	-1.185E-06	-1.138E-06	-1.170E-06	-1.172E-06	-1.156E-06	-1.159E-06	-1.150E-06	-1.152E-06
Δ_K	1.879E-05	1.879E-05	1.884E-05	1.794E-05	1.794E-05	1.811E-05	1.812E-05	1.837E-05	1.838E-05
δ_J	1.747E-08	1.734E-08	1.658E-08	1.691E-08	1.695E-08	1.686E-08	1.690E-08	1.683E-08	1.687E-08
δ_K	4.634E-07	4.403E-07	4.437E-07	4.156E-07	4.164E-07	4.235E-07	4.243E-07	4.330E-07	4.339E-07
MD%		-1.0841	-2.8418	-4.7626	-4.6087	-4.3184	-4.1418	-3.4913	-3.3054
MAD%		1.4758	2.9478	4.7626	4.6087	4.3184	4.1418	3.4913	3.3054
Max Pos		0.9793	0.2648	-0.3162	-0.1429	-1.4979	-1.2811	-2.0777	-1.8375
Max Neg		-4.9757	-5.0736	-10.2972	-10.1313	-8.6095	-8.4219	-6.5486	-6.3515

CH_2FCl	PW6B95-D3 jul-cc-pVDZ	PW6B95-D3 aug-cc-pVDZ	PW6B95-D3 jun-cc-pVDZ	B2PLYP-D3 VTZ	B2PLYP-D3 augVTZ	B2PLYP-D3 may'VTZ	rev-DSD-PBEP86-D3 jun-cc-pVTZ	rev-DSD-PBEP86-D3 aug-cc-pVTZ
Δ_J	1.227E-07	1.230E-07	1.209E-07	1.206E-07	1.213E-07	1.210E-07	1.200E-07	1.202E-07
Δ_{JK}	-1.200E-06	-1.203E-06	-1.164E-06	-1.162E-06	-1.159E-06	-1.158E-06	-1.159E-06	-1.160E-06
Δ_K	1.886E-05	1.887E-05	1.876E-05	1.908E-05	1.897E-05	1.901E-05	1.871E-05	1.870E-05
δ_J	1.742E-08	1.746E-08	1.701E-08	1.662E-08	1.683E-08	1.680E-08	1.679E-08	1.683E-08
δ_K	4.441E-07	4.452E-07	4.386E-07	4.411E-07	4.434E-07	4.425E-07	4.352E-07	4.353E-07
MD%	-0.4650	-0.2587	-2.1848	-2.2440	-1.9749	-2.0653	-2.8461	-2.7829
MAD%	1.4907	1.4130	2.1848	2.8650	2.3445	2.5385	2.8461	2.7829
Max Pos	2.1820	2.4469	-0.1545	1.5526	0.9240	1.1829	-0.4303	-0.4862
Max Neg	-4.1547	-3.9254	-5.3482	-4.8312	-4.2971	-4.5002	-6.0759	-6.0494

[a] From Ref. (Blanco et al., 1995)
[b] CCSD(T)/CBS + Δ (CV) + Δ(aug). From Ref. (Pietropolli Charmet et al., 2013).

Table S33. *cis*-ClHC=CHF Quartic Centrifugal Distortion Constants (in cm^{-1}) obtained at the different levels of theory and comparison to the experimental results.

	Exp[a]	Scaled CCSD(T)[b]	B3LYP-D3	ωB97		ωB97X		ωB97XD	
cis-ClHC=CHF		VTZ	SNSD	jul-cc-pVDZ	aug-cc-pVDZ	jul-cc-pVDZ	aug-cc-pVDZ	jul-cc-pVDZ	aug-cc-pVDZ
Δ_J	9.867E-08	8.923E-08	9.158E-08	9.541E-08	9.500E-08	9.466E-08	9.428E-08	9.361E-08	9.329E-08
Δ_{JK}	-6.663E-07	-6.345E-07	-6.500E-07	-6.621E-07	-6.589E-07	-6.616E-07	-6.586E-07	-6.745E-07	-6.710E-07
Δ_K	2.667E-06	2.621E-06	2.749E-06	2.612E-06	2.607E-06	2.656E-06	2.651E-06	2.786E-06	2.778E-06
δ_J	2.522E-08	2.252E-08	2.299E-08	2.441E-08	2.429E-08	2.409E-08	2.398E-08	2.359E-08	2.350E-08
δ_K	2.385E-07	2.086E-07	2.221E-07	2.230E-07	2.225E-07	2.232E-07	2.227E-07	2.236E-07	2.233E-07
MD%		-7.8637	-4.4492	-3.1375	-3.4938	-3.2211	-3.5473	-2.4230	-2.7622
MAD%		7.8637	5.6711	3.1375	3.4938	3.2211	3.5473	4.6980	4.6988
Max Pos		-1.7582	3.0547	-0.6180	-1.1014	-0.4466	-0.6097	4.4509	4.1356
Max Neg		-12.5175	-8.8170	-6.5135	-6.7163	-6.4318	-6.6064	-6.4458	-6.8267

	PW6B95-D3			B2PLYP-D3			rev-DSD-PBEP86-D3	
cis-ClHC=CHF	jul-cc-pVDZ	aug-cc-pVDZ	jun-cc-pVDZ	VTZ	augVTZ	may'VTZ	jun-cc-pVTZ	aug-cc-pVTZ
Δ_J	1.033E-07	1.029E-07	1.035E-07	9.458E-08	9.554E-08	9.459E-08	9.588E-08	9.681E-08
Δ_{JK}	-7.406E-07	-7.383E-07	-7.490E-07	-6.754E-07	-6.775E-07	-6.749E-07	-6.790E-07	-6.860E-07
Δ_K	2.913E-06	2.912E-06	2.953E-06	2.800E-06	2.798E-06	2.805E-06	2.770E-06	2.786E-06
δ_J	2.631E-08	2.620E-08	2.630E-08	2.384E-08	2.412E-08	2.385E-08	2.426E-08	2.452E-08
δ_K	2.417E-07	2.414E-07	2.425E-07	2.261E-07	2.281E-07	2.261E-07	2.274E-07	2.290E-07
MD%	6.1489	5.8803	6.7894	-1.6854	-1.0600	-1.6659	-1.1103	-0.2376
MAD%	6.1489	5.8803	6.7894	4.2262	3.6951	4.2408	3.4088	3.2017
Max Pos	11.1650	10.8100	12.4141	4.9809	4.9070	5.1414	3.8289	4.4488
Max Neg	1.3435	1.1991	1.6806	-5.4443	-4.3609	-5.4409	-4.6575	-3.9629

[a] From Ref. (Alonso et al., 1993)
[b] Scaled CCSD(T). From Ref. (Gambi et al., 2002)

Table S34. ClFC=CH_2 Quartic Centrifugal Distortion Constants (in cm^{-1}) obtained at the different levels of theory and comparison to the experimental results.

ClFC=CH_2	Exp[a]	CCSD(T)[b] CVTZ+CVTZ(F)	B3LYP-D3 SNSD	ωB97 jul-cc-pVDZ	ωB97 aug-cc-pVDZ	ωB97X jul-cc-pVDZ	ωB97X aug-cc-pVDZ	ωB97XD jul-cc-pVDZ	ωB97XD aug-cc-pVDZ
Δ_J	4.747E-08	4.640E-08	4.585E-08	4.461E-08	4.458E-08	4.508E-08	4.505E-08	4.574E-08	4.572E-08
Δ_{JK}	1.663E-07	1.682E-07	1.614E-07	1.537E-07	1.532E-07	1.561E-07	1.557E-07	1.580E-07	1.577E-07
Δ_K	1.720E-07	1.667E-07	1.622E-07	1.718E-07	1.714E-07	1.683E-07	1.679E-07	1.634E-07	1.629E-07
δ_J	1.612E-08	1.589E-08	1.559E-08	1.551E-08	1.550E-08	1.558E-08	1.556E-08	1.571E-08	1.569E-08
δ_K	1.813E-07	1.791E-07	1.746E-07	1.693E-07	1.689E-07	1.711E-07	1.707E-07	1.726E-07	1.724E-07
MD%		-1.3672	-3.8192	-4.8282	-5.0011	-4.4645	-4.6376	-4.2065	-4.3464
MAD%		1.8157	3.8192	4.8282	5.0011	4.4645	4.6376	4.2065	4.3464
Max Pos		1.1211	-2.9638	-0.1170	-0.3350	-2.1906	-2.4039	-2.5565	-2.6491
Max Neg		-3.1171	-5.7149	-7.6174	-7.9243	-6.1450	-6.4171	-5.0385	-5.3004

ClFC=CH_2	PW6B95-D3 jul-cc-pVDZ	PW6B95-D3 aug-cc-pVDZ	PW6B95-D3 jun-cc-pVDZ	B2PLYP-D3 VTZ	B2PLYP-D3 augVTZ	B2PLYP-D3 may'VTZ	rev-DSD-PBEP86-D3 jun-cc-pVTZ	rev-DSD-PBEP86-D3 aug-cc-pVTZ
Δ_J	4.658E-08	4.655E-08	4.613E-08	4.638E-08	4.643E-08	4.645E-08	4.611E-08	4.622E-08
Δ_{JK}	1.626E-07	1.623E-07	1.606E-07	1.640E-07	1.654E-07	1.635E-07	1.643E-07	1.652E-07
Δ_K	1.702E-07	1.700E-07	1.701E-07	1.593E-07	1.619E-07	1.640E-07	1.638E-07	1.626E-07
δ_J	1.610E-08	1.608E-08	1.591E-08	1.581E-08	1.583E-08	1.586E-08	1.581E-08	1.585E-08
δ_K	1.778E-07	1.776E-07	1.754E-07	1.765E-07	1.771E-07	1.763E-07	1.765E-07	1.770E-07
MD%	-1.4296	-1.5524	-2.3775	-3.1244	-2.5503	-2.5625	-2.6915	-2.5642
MAD%	1.4296	1.5524	2.3775	3.1244	2.5503	2.5625	2.6915	2.5642
Max Pos	-0.1026	-0.2081	-1.0978	-1.3916	-0.5747	-1.5694	-1.2485	-0.7077
Max Neg	-2.2570	-2.4208	-3.4330	-7.3913	-5.9089	-4.6862	-4.7845	-5.4706

[a] From Ref. (Leung et al., 2009).
[b] a.e.-CCSD(T)/cc-p CVTZ/aug-cc-pCVTZ(F), from Ref. (Pietropolli Charmet et al., 2016).

Table S35. ClFC=CF_2 Quartic Centrifugal Distortion Constants (in cm^{-1}) obtained at the different levels of theory and comparison to the experimental results.

ClFC=CF_2	Exp[a]	CCSD(T)[b] ANO2	B3LYP-D3 SNSD	ωB97 jul-cc-pVDZ	ωB97 aug-cc-pVDZ	ωB97X jul-cc-pVDZ	ωB97X aug-cc-pVDZ	ωB97XD jul-cc-pVDZ	ωB97XD aug-cc-pVDZ
Δ_J	7.094E-09	6.946E-09	6.734E-09	6.676E-09	6.676E-09	6.740E-09	6.740E-09	6.797E-09	6.797E-09
Δ_{JK}	2.088E-09	1.861E-09	2.468E-09	1.868E-09	1.868E-09	1.825E-09	1.825E-09	1.740E-09	1.740E-09
Δ_K	3.720E-08	3.615E-08	3.580E-08	3.578E-08	3.578E-08	3.614E-08	3.614E-08	3.660E-08	3.660E-08
d_1	-3.110E-09	-3.009E-09	-2.912E-09	-2.926E-09	-2.926E-09	-2.942E-09	-2.942E-09	-2.950E-09	-2.950E-09
d_2	-5.804E-10	-5.784E-10	-5.671E-10	-5.704E-10	-5.704E-10	-5.705E-10	-5.705E-10	-5.674E-10	-5.674E-10
MD%		-3.8710	0.1384	-5.5794	-5.5794	-5.5032	-5.5032	-5.9775	-5.9773
MAD%		3.8710	7.1342	5.5794	5.5794	5.5032	5.5032	5.9775	5.9773
Max Pos		-0.3448	18.1816	-1.7232	-1.7232	-1.7056	-1.7056	-1.6232	-1.6231
Max Neg		-10.8626	-6.3668	-10.5422	-10.5422	-12.5771	-12.5771	-16.6876	-16.6865

ClFC=CF_2	PW6B95-D3 jul-cc-pVDZ	PW6B95-D3 aug-cc-pVDZ	PW6B95-D3 jun-cc-pVDZ	B2PLYP-D3 VTZ	B2PLYP-D3 augVTZ	B2PLYP-D3 may'VTZ	rev-DSD-PBEP86-D3 jun-cc-pVTZ	rev-DSD-PBEP86-D3 aug-cc-pVTZ
Δ_J	7.127E-09	7.127E-09	7.117E-09	6.843E-09	6.912E-09	6.876E-09	6.910E-09	6.943E-09
Δ_{JK}	1.119E-09	1.119E-09	8.286E-10	2.400E-09	2.081E-09	2.178E-09	1.877E-09	1.719E-09
Δ_K	3.802E-08	3.802E-08	3.808E-08	3.540E-08	3.627E-08	3.592E-08	3.620E-08	3.644E-08
d_1	-3.117E-09	-3.117E-09	-3.104E-09	-2.968E-09	-2.996E-09	-2.981E-09	-3.000E-09	-3.015E-09
d_2	-5.957E-10	-5.957E-10	-5.879E-10	-5.779E-10	-5.791E-10	-5.775E-10	-5.781E-10	-5.790E-10
MD%	-8.1849	-8.1855	-11.3070	0.3136	-1.8566	-1.3718	-3.8677	-5.0262
MAD%	10.3845	10.3852	12.9080	5.6681	1.8566	3.0976	3.8677	5.0262
Max Pos	2.6323	2.6321	2.3757	14.9543	-0.2234	4.3147	-0.3975	-0.2349
Max Neg	-46.4234	-46.4268	-60.3164	-4.8242	-3.6683	-4.1593	-10.0971	-17.6562

[a] From Ref. (Hillig et al., 1988).
[b] CCSD(T)/ANOtz, from Ref. (Tasinato et al., 2012a).

Table S36. Oxirane Quartic Centrifugal Distortion Constants (in cm^{-1}) obtained at the different levels of theory and comparison to the experimental results.

Oxirane	Exp[a]	CCSD(T)[b] CBS + CV + aug	B3LYP-D3 SNSD	ωB97 jul-cc-pVDZ	ωB97 aug-cc-pVDZ	ωB97X jul-cc-pVDZ	ωB97X aug-cc-pVDZ	ωB97XD jul-cc-pVDZ	ωB97XD aug-cc-pVDZ
Δ_J	6.900E-07	6.850E-07	6.704E-07	6.160E-07	6.166E-07	6.298E-07	6.303E-07	6.469E-07	6.475E-07
Δ_{JK}	6.975E-07	7.215E-07	6.361E-07	5.638E-07	5.673E-07	5.593E-07	5.629E-07	5.531E-07	5.567E-07
Δ_K	9.204E-07	8.915E-07	9.478E-07	8.504E-07	8.488E-07	9.032E-07	9.013E-07	9.712E-07	9.692E-07
δ_J	2.072E-07	2.036E-07	2.026E-07	1.822E-07	1.824E-07	1.876E-07	1.878E-07	1.945E-07	1.947E-07
δ_K	6.040E-07	5.889E-07	5.647E-07	4.663E-07	4.683E-07	4.827E-07	4.848E-07	5.039E-07	5.059E-07
MD%		-0.9281	-3.4785	-14.4673	-14.2987	-11.9889	-11.8209	-8.8253	-8.6598
MAD%		2.3055	4.6737	14.4673	14.2987	11.9889	11.8209	11.0373	10.7832
Max Pos		3.4433	2.9878	-7.5983	-7.7765	-1.8655	-2.0721	5.5301	5.3086
Max Neg		-3.1402	-8.8074	-22.8000	-22.4731	-20.0851	-19.7431	-20.6990	-20.1901

Oxirane	PW6B95-D3 jul-cc-pVDZ	PW6B95-D3 aug-cc-pVDZ	PW6B95-D3 jun-cc-pVDZ	B2PYLP VTZ	B2PLYP-D3 augVTZ	B2PLYP-D3 may'VTZ	rev-DSD-PBEP86-D3 jun-cc-pVTZ	rev-DSD-PBEP86-D3 aug-cc-pVTZ
Δ_J	6.514E-07	6.514E-07	6.468E-07	6.805E-07	6.814E-07	6.808E-07	6.679E-07	6.687E-07
Δ_{JK}	6.107E-07	6.107E-07	5.941E-07	6.922E-07	7.232E-07	7.116E-07	6.964E-07	7.019E-07
Δ_K	9.243E-07	9.243E-07	9.353E-07	9.038E-07	8.955E-07	9.059E-07	8.792E-07	8.752E-07
δ_J	1.949E-07	1.949E-07	1.935E-07	2.044E-07	2.036E-07	2.038E-07	1.987E-07	1.989E-07
δ_K	5.218E-07	5.218E-07	5.111E-07	5.857E-07	5.945E-07	5.914E-07	5.713E-07	5.731E-07
MD%	-7.4270	-7.4270	-8.2881	-1.6605	-0.7152	-0.9229	-3.4701	-3.2955
MAD%	7.5993	7.5993	8.9371	1.6605	2.1909	1.7337	3.4701	3.5509
Max Pos	0.4308	0.4308	1.6225	-0.7598	3.6894	2.0269	-0.1541	0.6383
Max Neg	-13.6076	-13.6076	-15.3811	-3.0365	-2.6960	-2.0900	-5.4133	-5.1249

[a] From Ref. (Medcraft et al., 2012).
[b] CCSD(T)/CBS + Δ(CV) + Δ(aug), from Ref. (Puzzarini et al., 2014a).

Table S37. CH_3CH_2SH Quartic Centrifugal Distortion Constants (in cm^{-1}) obtained at the different levels of theory and comparison to the experimental results.

CH_3CH_2SH	Exp[a]	CCSD(T)[b] cc-pVTZ	B3LYP-D3 SNSD	ωB97 jul-cc-pVDZ	ωB97 aug-cc-pVDZ	ωB97X jul-cc-pVDZ	ωB97X aug-cc-pVDZ	ωB97XD jul-cc-pVDZ	ωB97XD aug-cc-pVDZ
Δ_J	1.110E-07	1.122E-07	1.066E-07	1.040E-07	1.040E-07	1.043E-07	1.047E-07	1.086E-07	1.050E-07
Δ_{JK}	-6.135E-07	-6.367E-07	-6.034E-07	-6.026E-07	-6.096E-07	-5.924E-07	-6.006E-07	-5.410E-07	-5.606E-07
Δ_K	6.810E-06	6.881E-06	6.940E-06	6.467E-06	6.454E-06	6.561E-06	6.560E-06	6.514E-06	6.755E-06
δ_J	1.716E-08	1.781E-08	1.584E-08	1.657E-08	1.654E-08	1.633E-08	1.643E-08	1.841E-08	1.534E-08
δ_K	2.929E-07	3.322E-07	2.735E-07	2.880E-07	2.919E-07	2.812E-07	2.902E-07	3.782E-07	2.219E-07
MD%		4.6390	-3.5833	-3.6441	-3.2157	-4.3772	-3.3063	3.6158	-9.9386
MAD%		4.6390	4.3496	3.6441	3.2157	4.3772	3.3063	10.9433	9.9386
Max Pos		13.4267	1.9157	-1.6761	-0.3490	-3.4379	-0.9316	29.1113	-0.8007
Max Neg		1.0472	-7.6657	-6.2897	-6.2477	-5.9642	-5.6094	-11.8211	-24.2489

CH_3CH_2SH	PW6B95-D3 jul-cc-pVDZ	PW6B95-D3 aug-cc-pVDZ	PW6B95-D3 jun-cc-pVDZ	B2PLYP-D3 VTZ	B2PLYP-D3 augVTZ	B2PLYP-D3 may'VTZ	rev-DSD-PBEP86-D3 jun-cc-pVTZ	rev-DSD-PBEP86-D3 aug-cc-pVTZ
Δ_J	1.109E-07	1.120E-07	1.110E-07	1.098E-07	1.099E-07	1.100E-07	1.110E-07	1.112E-07
Δ_{JK}	-6.614E-07	-6.759E-07	-6.905E-07	-6.415E-07	-6.395E-07	-6.482E-07	-6.566E-07	-6.545E-07
Δ_K	7.047E-06	7.073E-06	7.137E-06	7.000E-06	6.982E-06	7.036E-06	6.864E-06	6.838E-06
δ_J	1.720E-08	1.751E-08	1.716E-08	1.675E-08	1.676E-08	1.677E-08	1.735E-08	1.740E-08
δ_K	2.996E-07	3.119E-07	3.093E-07	2.990E-07	2.990E-07	3.066E-07	3.115E-07	3.124E-07
MD%	2.7554	4.6999	4.6035	1.2084	1.1167	2.0939	3.0619	3.0716
MAD%	2.7662	4.6999	4.6035	2.5646	2.4194	3.3621	3.0619	3.0716
Max Pos	7.8120	10.1705	12.5510	4.5637	4.2422	5.6557	7.0267	6.6807
Max Neg	-0.0269	0.9241	0.0279	-2.3729	-2.3474	-2.2792	0.0452	0.2123

[a] From Ref. (Kolesniková et al., 2014).
[b] CCSD(T)/cc-pVTZ, from Ref. (Puzzarini et al., 2014b).

Table S38. SO_2 Quartic Centrifugal Distortion Constants (in cm^{-1}) obtained at the different levels of theory and comparison to the experimental results.

	Exp[a]	Cheap[b]	B3LYP-D3	ωB97		ωB97X		ωB97XD	
SO_2			SNSD	jul-cc-pVDZ	aug-cc-pVDZ	jul-cc-pVDZ	aug-cc-pVDZ	jul-cc-pVDZ	aug-cc-pVDZ
Δ_J	2.205E-07	2.196E-07	2.211E-07	2.068E-07	2.068E-07	2.084E-07	2.084E-07	2.099E-07	2.099E-07
Δ_{JK}	-3.901E-06	-3.757E-06	-3.347E-06	-3.716E-06	-3.716E-06	-3.719E-06	-3.719E-06	-3.734E-06	-3.734E-06
Δ_K	8.640E-05	8.017E-05	6.140E-05	7.458E-05	7.458E-05	7.550E-05	7.550E-05	7.671E-05	7.671E-05
δ_J	5.674E-08	5.653E-08	5.905E-08	5.493E-08	5.493E-08	5.509E-08	5.509E-08	5.513E-08	5.513E-08
δ_K	8.463E-07	7.475E-07	7.103E-07	6.918E-07	6.918E-07	7.002E-07	7.002E-07	7.095E-07	7.095E-07
MD%		-4.6745	-10.9755	-9.2274	-9.2274	-8.5873	-8.5873	-7.8610	-7.8610
MAD%		4.6745	12.7010	9.2274	9.2274	8.5873	8.5873	7.8610	7.8610
Max Pos		-0.3792	4.0637	-3.1982	-3.1982	-2.9046	-2.9046	-2.8417	-2.8417
Max Neg		-11.6694	-28.9332	-18.2557	-18.2557	-17.2571	-17.2571	-16.1593	-16.1593

	PW6B95-D3			B2PLYP-D3			rev-DSD-PBEP86-D3	
SO_2	jul-cc-pVDZ	aug-cc-pVDZ	jun-cc-pVDZ	VTZ	augVTZ	may'VTZ	jun-cc-pVTZ	aug-cc-pVTZ
Δ_J	2.151E-07	2.130E-07	2.113E-07	2.209E-07	2.239E-07	2.188E-07	2.167E-07	2.174E-07
Δ_{JK}	-3.813E-06	-3.270E-06	-3.741E-06	-3.537E-06	-3.545E-06	-3.767E-06	-3.746E-06	-3.748E-06
Δ_K	7.871E-05	6.841E-05	7.863E-05	7.159E-05	7.131E-05	8.095E-05	7.996E-05	7.974E-05
δ_J	5.626E-08	5.479E-08	5.498E-08	5.706E-08	5.804E-08	5.594E-08	5.565E-08	5.585E-08
δ_K	7.276E-07	7.179E-07	7.198E-07	7.311E-07	7.392E-07	7.465E-07	7.361E-07	7.374E-07
MD%	-5.6984	-11.8086	-7.0703	-7.8710	-7.0861	-4.7490	-5.6183	-5.5039
MAD%	5.6984	11.8086	7.0703	8.1574	8.6108	4.7490	5.6183	5.5039
Max Pos	-0.8554	-3.4290	-3.1112	0.5518	2.2956	-0.7981	-1.7258	-1.4276
Max Neg	-14.0211	-20.8249	-14.9471	-17.1397	-17.4698	-11.7866	-13.0144	-12.8688

[a] From Ref. (Müller and Brünken, 2005).
[b] Cheap scheme, from Ref. (Boussessi et al., 2020).

Table S39. $HCOCH_2OH$ Quartic Centrifugal Distortion Constants (in cm^{-1}) obtained at the different levels of theory and comparison to the experimental results.

$HCOCH_2OH$	Exp[a]	Cheap[b]	B3LYP-D3 SNSD	ωB97 jul-cc-pVDZ	ωB97 aug-cc-pVDZ	ωB97X jul-cc-pVDZ	ωB97X aug-cc-pVDZ	ωB97XD jul-cc-pVDZ	ωB97XD aug-cc-pVDZ
Δ_J	2.075E-07	2.102E-07	2.077E-07	1.945E-07	1.934E-07	1.979E-07	1.975E-07	1.976E-07	1.975E-07
Δ_{JK}	-6.804E-07	-6.765E-07	-6.982E-07	-6.164E-07	-6.087E-07	-6.350E-07	-6.305E-07	-6.420E-07	-6.391E-07
Δ_K	1.591E-06	1.546E-06	1.599E-06	1.424E-06	1.410E-06	1.460E-06	1.450E-06	1.493E-06	1.485E-06
δ_J	6.118E-08	6.210E-08	6.118E-08	5.744E-08	5.711E-08	5.840E-08	5.827E-08	5.812E-08	5.808E-08
δ_K	2.958E-07	2.894E-07	2.859E-07	2.689E-07	2.679E-07	2.727E-07	2.725E-07	2.736E-07	2.736E-07
MD%		-0.5549	-0.0209	-8.2744	-8.9486	-6.3755	-6.7344	-5.8157	-6.0153
MAD%		1.6862	1.3167	8.2744	8.9486	6.3755	6.7344	5.8157	6.0153
Max Pos		1.5046	2.6176	-6.1062	-6.6534	-4.5340	-4.7487	-4.7459	-4.8036
Max Neg		-2.8536	-3.3440	-10.5062	-11.3637	-8.2586	-8.8653	-7.4941	-7.4870

$HCOCH_2OH$	PW6B95-D3 jul-cc-pVDZ	PW6B95-D3 aug-cc-pVDZ	PW6B95-D3 jun-cc-pVDZ	B2PLYP-D3 VTZ	B2PLYP-D3 augVTZ	B2PLYP-D3 may'VTZ	rev-DSD-PBEP86-D3 jun-cc-pVTZ	rev-DSD-PBEP86-D3 aug-cc-pVTZ
Δ_J	2.053E-07	2.040E-07	2.050E-07	2.035E-07	2.086E-07	2.065E-07	2.050E-07	2.054E-07
Δ_{JK}	-6.483E-07	-6.399E-07	-6.536E-07	-6.509E-07	-6.866E-07	-6.762E-07	-6.632E-07	-6.680E-07
Δ_K	1.472E-06	1.458E-06	1.486E-06	1.511E-06	1.581E-06	1.568E-06	1.530E-06	1.541E-06
δ_J	6.078E-08	6.038E-08	6.054E-08	5.997E-08	6.150E-08	6.081E-08	6.041E-08	6.053E-08
δ_K	2.824E-07	2.812E-07	2.796E-07	2.813E-07	2.875E-07	2.846E-07	2.823E-07	2.831E-07
MD%	-3.6878	-4.4494	-3.6437	-3.6272	-0.2929	-1.3956	-2.6717	-2.2672
MAD%	3.6878	4.4494	3.6437	3.6272	1.0909	1.3956	2.6717	2.2672
Max Pos	-0.6425	-1.3063	-1.0378	-1.9362	0.9107	-0.4912	-1.1937	-0.9928
Max Neg	-7.4978	-8.3657	-6.6039	-4.9965	-2.8160	-3.7959	-4.5502	-4.3012

[a] From Ref. (Carroll et al., 2010).
[b] Cheap scheme, from Ref. (Boussessi et al., 2020).

Table S40. *E*-Ethanimine Quartic Centrifugal Distortion Constants (in cm^{-1}) obtained at the different levels of theory and comparison to the experimental results.

E-Ethanimine	Exp[a]	CCSD(T)[b] cc-pCVQZ	B3LYP-D3 SNSD	ωB97 jul-cc-pVDZ	ωB97 aug-cc-pVDZ	ωB97X jul-cc-pVDZ	ωB97X aug-cc-pVDZ	ωB97XD jul-cc-pVDZ	ωB97XD aug-cc-pVDZ
Δ_J	2.156E-07	2.161E-07	2.092E-07	2.043E-07	2.049E-07	2.054E-07	2.060E-07	2.074E-07	2.079E-07
Δ_{JK}	-4.740E-07	-5.504E-07	-5.661E-07	-5.442E-07	-5.174E-07	-5.210E-07	-4.946E-07	-5.143E-07	-4.911E-07
Δ_K	1.916E-05	1.895E-05	1.899E-05	1.802E-05	1.807E-05	1.815E-05	1.819E-05	1.835E-05	1.838E-05
δ_J	3.683E-08	3.636E-08	3.481E-08	3.469E-08	3.478E-08	3.469E-08	3.478E-08	3.486E-08	3.494E-08
δ_K	-2.232E-06	-1.785E-06	-1.654E-06	-1.740E-06	-1.828E-06	-1.788E-06	-1.874E-06	-1.801E-06	-1.870E-06
MD%		-1.2115	-3.1567	-4.8421	-5.0285	-5.1577	-5.3411	-4.8338	-5.0736
MAD%		7.7653	10.9328	10.7663	8.6911	9.1223	7.0786	8.2389	6.5165
Max Pos		16.1154	19.4403	14.8104	9.1566	9.9114	4.3435	8.5128	3.6074
Max Neg		-20.0418	-25.8724	-22.0261	-18.0874	-19.8835	-16.0204	-19.2962	-16.2000

E-Ethanimine	PW6B95-D3 jul-cc-pVDZ	PW6B95-D3 aug-cc-pVDZ	PW6B95-D3 jun-cc-pVDZ	B2PLYP-D3 VTZ	B2PLYP-D3 augVTZ	B2PLYP-D3 may'VTZ	rev-DSD-PBEP86-D3 jun-cc-pVTZ	rev-DSD-PBEP86-D3 aug-cc-pVTZ
Δ_J	2.131E-07	2.137E-07	2.104E-07	2.136E-07	2.132E-07	2.128E-07	2.125E-07	2.128E-07
Δ_{JK}	-6.553E-07	-6.290E-07	-5.937E-07	-5.763E-07	-5.583E-07	-5.404E-07	-5.701E-07	-5.725E-07
Δ_K	1.910E-05	1.916E-05	1.893E-05	1.906E-05	1.925E-05	1.920E-05	1.895E-05	1.896E-05
δ_J	3.626E-08	3.634E-08	3.568E-08	3.560E-08	3.547E-08	3.539E-08	3.560E-08	3.569E-08
δ_K	-1.634E-06	-1.715E-06	-1.703E-06	-1.633E-06	-1.717E-06	-1.750E-06	-1.707E-06	-1.709E-06
MD%	1.6850	1.4779	-1.0325	-2.0002	-1.9346	-2.5231	-1.8285	-1.6141
MAD%	13.6132	11.6129	11.1303	10.6372	9.2255	8.2024	9.9403	9.9305
Max Pos	38.2456	32.7036	25.2444	21.5925	17.7772	13.9994	20.2794	20.7910
Max Neg	-26.8014	-23.1395	-23.6790	-26.8303	-23.0816	-21.5901	-23.5266	-23.4260

[a] From Ref. (Melli et al., 2018).
[b] a.e.-CCSD(T)/cc-pCVQZ,, from Ref. (Melli et al., 2018).

1.6 Harmonic Frequencies

Table S41. CH_2F_2 Harmonic Frequencies in cm^{-1} obtained at the different levels of theory.

	CCSD(T)[a]	B3LYP-D3	ωB97		ωB97X		ωB97XD	
CH_2F_2	aug-cc-pCVQZ	SNSD	jul-cc-pVDZ	aug-cc-pVDZ	jul-cc-pVDZ	aug-cc-pVDZ	jul-cc-pVDZ	aug-cc-pVDZ
ω_1	3085	3066	3110	3112	3102	3103	3078	3079
ω_2	1552	1523	1500	1494	1509	1502	1513	1506
ω_3	1134	1102	1110	1110	1116	1117	1114	1115
ω_4	535	519	513	513	518	518	520	520
ω_5	1289	1264	1245	1244	1256	1255	1260	1259
ω_6	3160	3141	3206	3206	3192	3192	3166	3165
ω_7	1200	1172	1158	1158	1166	1166	1169	1169
ω_8	1470	1446	1422	1421	1432	1431	1437	1435
ω_9	1119	1067	1089	1087	1093	1092	1088	1086
MD		-27.2	-21.1	-22.0	-17.7	-18.8	-22.0	-23.4
MAD		27.2	36.9	38.3	28.4	29.9	23.3	24.5
Max Pos		-16.5	45.9	46.3	31.8	31.9	5.6	4.6
Max Neg		-51.8	-51.8	-58.1	-42.7	-49.8	-38.7	-46.5

	PW6B95-D3			B2PLYP-D3			rev-DSD-PBEP86-D3	
CH_2F2	jul-cc-pVDZ	aug-cc-pVDZ	jun-cc-pVDZ	VTZ	augVTZ	may'VTZ	jun-cc-pVTZ	aug-cc-pVTZ
ω_1	3099	3102	3095	3083	3088	3091	3087	3085
ω_2	1513	1503	1501	1555	1549	1550	1551	1553
ω_3	1118	1118	1123	1126	1114	1117	1126	1124
ω_4	519	519	528	531	526	528	531	530
ω_5	1257	1257	1260	1288	1280	1285	1286	1282
ω_6	3184	3186	3180	3158	3164	3166	3162	3161
ω_7	1166	1167	1164	1200	1191	1193	1196	1194
ω_8	1431	1431	1441	1480	1465	1470	1469	1468
ω_9	1093	1092	1100	1107	1082	1087	1104	1102
MD	-18.3	-18.7	-16.8	-1.8	-9.4	-6.3	-3.7	-5.1
MAD	26.6	28.2	23.4	4.7	11.1	8.9	4.4	5.4
Max Pos	23.8	25.9	20.1	10.0	4.4	5.9	1.7	0.7
Max Neg	-39.1	-49.0	-50.6	-12.3	-36.7	-32.2	-15.4	-17.0

[a] a.e.-CCSD(T)/aug-cc-pCVQZ level. From Ref. (Tasinato et al., 2012b).

Table S42. CH_2FCl Harmonic Frequencies in cm^{-1} obtained at the different levels of theory.

CH_2FCl	CCSD(T)[a] CBS+CV+aug	B3LYP-D3 SNSD	ωB97 jul-cc-pVDZ	ωB97 aug-cc-pVDZ	ωB97X jul-cc-pVDZ	ωB97X aug-cc-pVDZ	ωB97XD jul-cc-pVDZ	ωB97XD aug-cc-pVDZ
ω_1	3111.10	3097	3131	3132	3125	3126	3104	3104
ω_2	1512.80	1493	1476	1467	1483	1473	1484	1473
ω_3	1379.30	1362	1361	1360	1363	1361	1358	1356
ω_4	1087.50	1067	1072	1072	1079	1078	1077	1078
ω_5	781.20	731	817	816	795	794	769	768
ω_6	388.30	376	392	392	390	390	386	385
ω_7	3190.10	3179	3223	3223	3214	3215	3194	3193
ω_8	1264.10	1246	1237	1235	1243	1242	1243	1242
ω_9	1022.40	999	1007	1007	1007	1007	1001	1002
MD		-20.7	-2.3	-3.6	-4.3	-5.7	-13.5	-15.3
MAD		20.7	22.9	24.3	16.3	17.7	14.2	15.9
Max Pos		-11.5	35.9	35.3	24.3	24.5	3.5	2.6
Max Neg		-50.0	-36.7	-45.7	-30.2	-39.9	-29.1	-39.6

CH_2FCl	PW6B95-D3 jul-cc-pVDZ	PW6B95-D3 aug-cc-pVDZ	PW6B95-D3 jun-cc-pVDZ	B2PLYP-D3 VTZ	B2PLYP-D3 augVTZ	B2PLYP-D3 may'VTZ	rev-DSD-PBEP86-D3 jun-cc-pVTZ	rev-DSD-PBEP86-D3 aug-cc-pVTZ
ω_1	3124	3125	3121	3115	3116	3116	3111	3110
ω_2	1483	1470	1476	1521	1516	1517	1517	1519
ω_3	1351	1350	1359	1388	1383	1386	1388	1388
ω_4	1077	1078	1083	1094	1073	1075	1087	1085
ω_5	768	767	770	751	751	755	770	770
ω_6	384	383	387	383	382	384	387	387
ω_7	3212	3212	3207	3194	3195	3194	3189	3188
ω_8	1239	1238	1243	1269	1262	1266	1268	1265
ω_9	999	999	997	1018	1017	1016	1020	1021
MD	-11.1	-12.6	-10.5	-0.4	-4.5	-2.9	-0.0	-0.4
MAD	18.8	20.7	16.6	8.4	8.4	8.0	3.7	3.9
Max Pos	21.6	22.3	17.3	8.6	5.4	7.1	8.3	8.2
Max Neg	-30.1	-43.1	-37.1	-30.0	-29.9	-26.1	-10.8	-11.2

[a] CCSD(T)/CBS + Δ(CV) + Δ(aug), from Ref. (Pietropolli Charmet et al., 2013)

Table S43. *cis*-ClHC=CHF Harmonic Frequencies in cm^{-1} obtained at the different levels of theory.

cis-ClHC=CHF	Cheap[a]	B3LYP-D3 SNSD	ωB97 jul-cc-pVDZ	ωB97 aug-cc-pVDZ	ωB97X jul-cc-pVDZ	ωB97X aug-cc-pVDZ	ωB97XD jul-cc-pVDZ	ωB97XD aug-cc-pVDZ
ω_1	3248	3243	3286	3288	3279	3282	3264	3267
ω_2	3227	3213	3256	3260	3249	3254	3232	3236
ω_3	1695	1711	1775	1774	1769	1768	1750	1749
ω_4	1348	1343	1340	1346	1345	1352	1346	1351
ω_5	1244	1246	1238	1240	1248	1249	1255	1255
ω_6	1067	1066	1074	1075	1078	1079	1078	1079
ω_7	820	802	846	846	836	836	822	822
ω_8	661	651	671	672	670	671	665	665
ω_9	195	197	199	199	199	199	197	198
ω_{10}	863	878	921	922	918	919	907	907
ω_{11}	738	746	766	770	765	769	760	764
ω_{12}	445	452	463	464	463	464	461	461
MD		-0.2	23.6	25.4	22.4	24.2	15.5	17.0
MAD		8.5	25.9	26.4	22.9	24.2	15.9	17.0
Max Pos		16.0	80.1	79.1	73.9	72.8	55.0	53.8
Max Neg		-18.0	-8.4	-3.8	-2.9	3.3	-2.4	2.1

cis-ClHC=CHF	PW6B95-D3 jul-cc-pVDZ	PW6B95-D3 aug-cc-pVDZ	PW6B95-D3 jun-cc-pVDZ	B2PLYP-D3 VTZ	B2PLYP-D3 augVTZ	B2PLYP-D3 may'VTZ	rev-DSD-PBEP86-D3 jun-cc-pVTZ	rev-DSD-PBEP86-D3 aug-cc-pVTZ
ω_1	3277	3281	3276	3261	3259	3258	3256	3254
ω_2	3251	3256	3252	3230	3231	3231	3229	3227
ω_3	1746	1744	1748	1715	1706	1708	1714	1712
ω_4	1341	1347	1340	1368	1361	1365	1364	1362
ω_5	1247	1248	1248	1265	1258	1263	1261	1258
ω_6	1077	1078	1080	1083	1074	1077	1082	1080
ω_7	825	825	825	815	812	815	822	820
ω_8	665	666	666	662	660	663	666	665
ω_9	191	191	190	198	197	198	198	197
ω_{10}	896	896	892	897	893	899	895	887
ω_{11}	755	759	754	761	756	760	759	756
ω_{12}	460	460	459	460	457	459	457	456
MD	15.1	16.7	15.0	13.8	9.6	12.0	12.7	10.2
MAD	17.0	17.7	17.3	14.7	11.0	12.8	12.7	10.2
Max Pos	50.7	49.5	53.3	34.3	30.1	35.9	32.3	24.8
Max Neg	-6.9	-4.3	-8.6	-5.2	-8.2	-5.2	1.6	-0.0

[a] Cheap scheme, from Ref. (Boussessi et al., 2020).

Table S44. ClFC=CH_2 Harmonic Frequencies in cm^{-1} obtained at the different levels of theory.

ClFC=CH_2	CCSD(T)[a] CVTZ+CVTZ(F)	B3LYP-D3 SNSD	ωB97 jul-cc-pVDZ	ωB97 aug-cc-pVDZ	ωB97X jul-cc-pVDZ	ωB97X aug-cc-pVDZ	ωB97XD jul-cc-pVDZ	ωB97XD aug-cc-pVDZ
ω_1	3305	3287	3342	3344	3334	3337	3319	3321
ω_2	3195	3183	3222	3227	3216	3222	3202	3207
ω_3	1709	1712	1773	1772	1766	1766	1746	1746
ω_4	1412	1398	1375	1385	1383	1392	1388	1397
ω_5	1210	1176	1201	1205	1200	1204	1193	1196
ω_6	962	950	944	946	950	953	953	956
ω_7	706	689	730	731	721	721	708	708
ω_8	432	429	433	433	435	435	434	435
ω_9	369	370	376	377	377	377	376	377
ω_{10}	851	856	883	901	881	900	870	889
ω_{11}	724	717	725	725	727	727	723	723
ω_{12}	523	527	543	544	542	542	537	537
MD		-8.8	12.4	15.9	11.0	14.6	4.2	7.5
MAD		11.0	23.2	24.0	19.5	20.5	12.9	13.8
Max Pos		4.7	64.3	63.9	57.8	57.4	37.9	37.6
Max Neg		-34.0	-37.1	-27.8	-29.5	-20.3	-24.8	-15.9

ClFC=CH_2	PW6B95-D3 jul-cc-pVDZ	PW6B95-D3 aug-cc-pVDZ	PW6B95-D3 jun-cc-pVDZ	B2PLYP-D3 VTZ	B2PLYP-D3 augVTZ	B2PLYP-D3 may'VTZ	rev-DSD-PBEP86-D3 jun-cc-pVTZ	rev-DSD-PBEP86-D3 aug-cc-pVTZ
ω_1	3336	3339	3339	3312	3309	3309	3307	3305
ω_2	3218	3224	3221	3205	3203	3204	3199	3198
ω_3	1744	1744	1748	1713	1707	1707	1712	1711
ω_4	1383	1393	1385	1417	1414	1415	1414	1411
ω_5	1190	1194	1195	1205	1193	1196	1208	1205
ω_6	949	952	952	966	961	961	965	963
ω_7	710	710	711	699	698	701	708	707
ω_8	430	431	434	436	434	435	435	435
ω_9	371	371	373	374	372	374	372	371
ω_{10}	858	881	861	865	863	867	862	857
ω_{11}	721	721	719	731	727	728	730	728
ω_{12}	535	534	537	538	534	536	534	532
MD	3.7	7.9	6.4	5.1	1.1	2.8	3.9	1.9
MAD	15.0	16.3	15.9	7.2	6.0	6.5	4.4	3.1
Max Pos	35.5	35.1	39.2	15.0	11.9	15.8	10.8	8.9
Max Neg	-29.4	-19.4	-27.1	-7.4	-17.8	-14.8	-2.8	-5.3

[a] a.e.-CCSD(T)/cc-p CVTZ/aug-cc-pCVTZ(F), from Ref. (Pietropolli Charmet et al., 2016).

Table S45. ClFC=CF_2 Harmonic Frequencies in cm^{-1} obtained at the different levels of theory.

ClFC=CF_2	CCSD(T)[a] ANO2	B3LYP-D3 SNSD	ωB97 jul-cc-pVDZ	ωB97 aug-cc-pVDZ	ωB97X jul-cc-pVDZ	ωB97X aug-cc-pVDZ	ωB97XD jul-cc-pVDZ	ωB97XD aug-cc-pVDZ
ω_1	1842	1841	1909	1909	1904	1904	1884	1884
ω_2	1367	1316	1336	1336	1345	1345	1345	1345
ω_3	1241	1202	1223	1223	1225	1225	1221	1221
ω_4	1075	1053	1085	1085	1083	1083	1074	1074
ω_5	699	693	705	705	706	706	703	703
ω_6	521	513	512	512	516	516	518	518
ω_7	464	456	467	467	468	468	464	464
ω_8	340	339	339	339	341	341	342	342
ω_9	188	188	188	188	188	188	189	189
ω_{10}	546	567	607	607	607	607	595	595
ω_{11}	372	374	386	386	386	386	385	385
ω_{12}	170	168	173	173	174	174	172	172
MD		-9.6	8.9	8.9	10.0	10.0	5.5	5.5
MAD		13.5	18.7	18.7	17.0	17.0	13.3	13.3
Max Pos		21.1	66.7	66.7	62.1	62.1	49.3	49.3
Max Neg		-51.3	-30.7	-30.7	-21.9	-21.9	-22.3	-22.3

ClFC=CF_2	PW6B95-D3 jul-cc-pVDZ	PW6B95-D3 aug-cc-pVDZ	PW6B95-D3 jun-cc-pVDZ	B2PLYP-D3 VTZ	B2PLYP-D3 augVTZ	B2PLYP-D3 may'VTZ	rev-DSD-PBEP86-D3 jun-cc-pVTZ	rev-DSD-PBEP86-D3 aug-cc-pVTZ
ω_1	1883	1883	1886	1844	1838	1838	1847	1846
ω_2	1347	1347	1354	1349	1334	1338	1354	1351
ω_3	1222	1222	1227	1226	1213	1216	1231	1228
ω_4	1077	1077	1079	1069	1066	1069	1078	1077
ω_5	704	704	708	702	699	701	704	703
ω_6	515	515	518	522	519	521	521	521
ω_7	465	465	467	464	463	464	467	467
ω_8	336	336	337	341	340	342	341	341
ω_9	181	181	180	190	188	190	188	188
ω_{10}	584	584	581	572	570	577	575	568
ω_{11}	380	380	382	382	377	380	381	379
ω_{12}	172	172	172	172	171	172	173	172
MD	3.3	3.3	5.4	0.6	-4.0	-1.4	3.0	1.3
MAD	12.8	12.8	12.2	7.2	9.0	9.2	6.9	6.2
Max Pos	40.5	40.5	43.7	26.1	23.9	31.1	29.3	21.9
Max Neg	-20.4	-20.4	-13.9	-18.3	-33.4	-28.5	-13.2	-15.9

[a] CCSD(T)/ANOtz, from Ref. (Tasinato et al., 2012a).

Table S46. Oxirane Harmonic Frequencies in cm^{-1} obtained at the different levels of theory.

Oxirane	CCSD(T)[a] CBS+CV+aug	B3LYP-D3 SNSD (PES1)	ωB97 jul-cc-pVDZ	ωB97 aug-cc-pVDZ	ωB97X jul-cc-pVDZ	ωB97X aug-cc-pVDZ	ωB97XD jul-cc-pVDZ	ωB97XD aug-cc-pVDZ
ω_1	3125	3094	3147	3151	3138	3143	3117	3122
ω_2	1537	1536	1543	1548	1542	1548	1537	1542
ω_3	1299	1302	1333	1335	1331	1332	1321	1322
ω_4	1148	1146	1167	1171	1163	1168	1157	1161
ω_5	898	891	942	943	932	932	913	914
ω_6	3210	3170	3236	3239	3225	3228	3204	3207
ω_7	1177	1172	1160	1162	1164	1166	1163	1165
ω_8	1051	1043	1045	1043	1048	1046	1047	1046
ω_9	3223	3186	3249	3252	3239	3242	3219	3222
ω_{10}	1168	1166	1166	1165	1171	1169	1170	1168
ω_{11}	819	816	810	814	813	817	815	819
ω_{12}	3118	3088	3135	3141	3128	3134	3108	3114
ω_{13}	1501	1501	1478	1486	1484	1492	1486	1494
ω_{14}	1170	1158	1159	1160	1161	1162	1159	1160
ω_{15}	845	848	918	889	907	907	888	888
MD		-11.4	13.4	14.0	10.5	13.2	1.2	3.6
MAD		12.2	22.5	21.6	16.9	17.8	11.1	9.7
Max Pos		3.1	73.2	45.7	62.1	61.5	43.1	42.5
Max Neg		-39.1	-23.0	-15.7	-16.5	-11.0	-14.7	-12.2

Oxirane	PW6B95-D3 jul-cc-pVDZ	PW6B95-D3 aug-cc-pVDZ	PW6B95-D3 jun-cc-pVDZ	B2PYLP VTZ (PES5)	B2PLYP-D3 augVTZ (PES4)	B2PLYP-D3 may'VTZ (PES2)	rev-DSD-PBEP86-D3 jun-cc-pVTZ	rev-DSD-PBEP86-D3 aug-cc-pVTZ
ω_1	3132	3139	3131	3122	3125	3125	3125	3123
ω_2	1533	1539	1534	1552	1548	1550	1550	1548
ω_3	1322	1323	1326	1306	1303	1305	1308	1306
ω_4	1147	1152	1153	1156	1153	1156	1159	1156
ω_5	916	917	921	895	889	890	902	901
ω_6	3220	3225	3217	3200	3204	3203	3205	3203
ω_7	1159	1162	1159	1184	1184	1184	1185	1181
ω_8	1043	1043	1039	1054	1053	1055	1057	1053
ω_9	3234	3239	3231	3215	3219	3218	3220	3218
ω_{10}	1165	1165	1164	1176	1172	1174	1176	1173
ω_{11}	807	811	812	824	824	823	823	820
ω_{12}	3124	3131	3122	3115	3118	3119	3117	3115
ω_{13}	1484	1493	1480	1519	1516	1518	1514	1513
ω_{14}	1152	1154	1149	1162	1172	1174	1173	1169
ω_{15}	880	880	888	843	837	839	852	850
MD	2.1	5.7	2.6	2.4	1.9	3.1	5.2	2.7
MAD	12.8	13.5	13.9	7.0	5.4	6.4	6.3	5.1
Max Pos	35.1	35.0	42.6	17.9	15.2	17.3	13.6	11.8
Max Neg	-18.4	-15.6	-20.6	-9.2	-8.4	-7.8	-4.4	-6.9

[a] CCSD(T)/CBS + Δ(CV) + Δ(aug), from Ref. (Puzzarini et al., 2014a).

Table S47. SO_2 Harmonic Frequencies in cm^{-1} obtained at the different levels of theory.

SO_2	Cheap[a]	B3LYP-D3 SNSD	ωB97 jul-cc-pVDZ	ωB97 aug-cc-pVDZ	ωB97X jul-cc-pVDZ	ωB97X aug-cc-pVDZ	ωB97XD jul-cc-pVDZ	ωB97XD aug-cc-pVDZ
ω_1	1167	1102	1211	1211	1202	1202	1189	1189
ω_2	522	489	520	520	520	520	517	517
ω_3	1374	1257	1404	1404	1396	1396	1384	1384
MD		-71.7	23.4	23.4	17.8	17.8	8.7	8.7
MAD		71.7	24.9	24.9	19.6	19.6	12.1	12.1
Max Pos		-32.9	43.4	43.4	34.7	34.7	21.6	21.6
Max Neg		-117.0	-2.3	-2.3	-2.7	-2.7	-5.1	-5.1

SO_2	PW6B95-D3 jul-cc-pVDZ	PW6B95-D3 aug-cc-pVDZ	PW6B95-D3 jun-cc-pVDZ	B2PLYP-D3 VTZ	B2PLYP-D3 augVTZ	B2PLYP-D3 may'VTZ	rev-DSD-PBEP86-D3 jun-cc-pVTZ	rev-DSD-PBEP86-D3 aug-cc-pVTZ
ω_1	1168	1124	1178	1122	1112	1139	1151	1149
ω_2	510.26	516	518	507	503	511	515	514
ω_3	1364	1307	1378	1321	1304	1349	1364	1361
MD	-7.6	-39.4	3.1	-38.0	-48.4	-21.6	-11.4	-13.4
MAD	7.7	39.4	6.3	38.0	48.4	21.6	11.4	13.4
Max Pos	0.2	-6.6	10.6	-15.0	-19.5	-11.0	-7.5	-8.4
Max Neg	-12.2	-67.8	-4.8	-53.1	-70.1	-28.6	-16.2	-18.2

[a] Cheap scheme, from Ref. (Boussessi et al., 2020).

Table S48. $HCOCH_2OH$ Harmonic Frequencies in cm^{-1} obtained at the different levels of theory.

$HCOCH_2OH$	Cheap[a]	B3LYP-D3 SNSD	ωB97 jul-cc-pVDZ	ωB97 aug-cc-pVDZ	ωB97X jul-cc-pVDZ	ωB97X aug-cc-pVDZ	ωB97XD jul-cc-pVDZ	ωB97XD aug-cc-pVDZ
ω_1	3730	3707	3776	3774	3782	3780	3783	3780
ω_2	3017	2979	3047	3050	3031	3034	3004	3006
ω_3	2986	2938	3006	3011	2992	2996	2962	2967
ω_4	1772	1794	1836	1837	1835	1836	1827	1828
ω_5	1489	1467	1465	1461	1469	1465	1468	1464
ω_6	1448	1431	1449	1448	1449	1448	1445	1444
ω_7	1389	1381	1373	1378	1380	1385	1381	1386
ω_8	1301	1289	1307	1304	1308	1305	1306	1304
ω_9	1141	1137	1165	1165	1166	1166	1163	1163
ω_{10}	881	869	900	900	897	897	887	887
ω_{11}	760	762	765	765	768	769	768	768
ω_{12}	288	283	296	297	294	295	294	294
ω_{13}	3043	2993	3088	3091	3066	3068	3034	3035
ω_{14}	1264	1246	1249	1248	1253	1252	1253	1252
ω_{15}	1105	1096	1104	1107	1106	1110	1103	1107
ω_{16}	728	715	723	726	725	728	722	725
ω_{17}	414	413	405	412	407	414	404	409
ω_{18}	218	222	214	216	217	219	218	220
MD		-14.0	10.8	12.0	9.5	10.7	2.7	3.7
MAD		17.2	19.0	18.7	15.3	15.3	14.7	13.7
Max Pos		22.6	64.3	65.0	63.1	63.9	54.8	55.8
Max Neg		-49.8	-24.8	-28.6	-20.5	-24.7	-23.4	-25.2

[a] Cheap scheme, from Ref. (Boussessi et al., 2020).

$HCOCH_2OH$	PW6B95-D3 jul-cc-pVDZ	PW6B95-D3 aug-cc-pVDZ	PW6B95-D3 jun-cc-pVDZ	B2PLYP-D3 VTZ	B2PLYP-D3 augVTZ	B2PLYP-D3 may'VTZ	rev-DSD-PBEP86-D3 jun-cc-pVTZ	rev-DSD-PBEP86-D3 aug-cc-pVTZ
ω_1	3745	3743	3721	3724	3715	3727	3736	3731
ω_2	3020	3023	3019	3006	3009	3010	3011	3009
ω_3	2981	2986	2984	2964	2967	2968	2974	2971
ω_4	1818	1819	1826	1778	1768	1770	1778	1777
ω_5	1464	1459	1456	1496	1487	1491	1490	1491
ω_6	1436	1436	1432	1453	1444	1446	1452	1451
ω_7	1378	1383	1376	1402	1397	1400	1398	1397
ω_8	1296	1295	1296	1310	1300	1301	1305	1305
ω_9	1156	1155	1157	1141	1134	1136	1142	1142
ω_{10}	893	892	893	877	874	875	880	879
ω_{11}	765	766	767	768	763	763	763	763
ω_{12}	293	295	292	292	286	287	288	288
ω_{13}	3043	3046	3043	3027	3029	3030	3035	3034
ω_{14}	1245	1245	1244	1262	1261	1261	1263	1262
ω_{15}	1098	1102	1097	1116	1110	1112	1112	1111
ω_{16}	715	718	715	734	728	729	731	730
ω_{17}	418	422	404	414	409	403	402	403
ω_{18}	226	227	212	217	217	210	208	210
MD	0.9	2.2	-2.1	0.2	-4.2	-3.1	-0.3	-1.3
MAD	11.7	11.9	12.8	7.2	5.9	5.6	5.3	4.9
Max Pos	46.1	47.1	54.6	12.7	7.9	10.5	8.8	7.9
Max Neg	-25.3	-30.2	-32.9	-21.9	-19.1	-18.2	-12.2	-14.7

Table S49. *E*-Ethanimine Harmonic Frequencies in cm^{-1} obtained at the different levels of theory.

E-Ethanimine	CCSD(T)[a] pCVQZ	B3LYP-D3 SNSD	ωB97 jul-cc-pVDZ	ωB97 aug-cc-pVDZ	ωB97X jul-cc-pVDZ	ωB97X aug-cc-pVDZ	ωB97XD jul-cc-pVDZ	ωB97XD aug-cc-pVDZ
ω_1	3468	3460	3534	3537	3524	3526	3503	3504
ω_2	3156	3131	3205	3205	3189	3190	3166	3166
ω_3	3048	3023	3080	3082	3070	3071	3048	3048
ω_4	3039	3001	3068	3071	3055	3058	3029	3033
ω_5	1709	1724	1781	1780	1773	1773	1755	1754
ω_6	1483	1464	1454	1443	1459	1448	1460	1448
ω_7	1436	1429	1438	1435	1440	1438	1435	1434
ω_8	1395	1384	1374	1370	1379	1375	1378	1374
ω_9	1280	1272	1276	1274	1279	1278	1281	1280
ω_{10}	1067	1057	1070	1067	1071	1067	1067	1064
ω_{11}	933	923	942	938	941	938	936	933
ω_{12}	488	488	491	490	492	491	491	490
ω_{13}	3109	3073	3165	3165	3145	3146	3118	3118
ω_{14}	1489	1471	1461	1449	1467	1454	1467	1454
ω_{15}	1122	1116	1130	1132	1130	1133	1124	1127
ω_{16}	1081	1067	1069	1064	1072	1068	1071	1067
ω_{17}	680	675	682	679	683	681	681	680
ω_{18}	187	191	188	184	187	183	186	183
MD		-12.3	13.2	10.9	10.4	8.2	1.4	-0.8
MAD		14.5	23.6	25.5	18.3	20.4	10.8	12.8
Max Pos		14.7	71.7	71.0	64.2	63.6	45.6	45.4
Max Neg		-37.7	-28.9	-39.9	-23.6	-35.4	-23.5	-35.3

[a] a.e.-CCSD(T)/cc-pCVQZ, from Ref. (Boussessi et al., 2020).

E-Ethanimine	PW6B95-D3 jul-cc-pVDZ	PW6B95-D3 aug-cc-pVDZ	PW6B95-D3 jun-cc-pVDZ	B2PLYP-D3 VTZ	B2PLYP-D3 augVTZ	B2PLYP-D3 may'VTZ	rev-DSD-PBEP86-D3 jun-cc-pVTZ	rev-DSD-PBEP86-D3 aug-cc-pVTZ
ω_1	3496	3499	3487	3469	3470	3466	3471	3467
ω_2	3183	3184	3185	3158	3155	3158	3155	3153
ω_3	3064	3065	3066	3050	3048	3051	3046	3044
ω_4	3041	3045	3039	3028	3030	3029	3035	3033
ω_5	1752	1752	1758	1715	1706	1708	1710	1709
ω_6	1460	1445	1445	1484	1484	1486	1484	1485
ω_7	1433	1431	1431	1438	1434	1436	1435	1433
ω_8	1375	1369	1372	1399	1398	1400	1396	1396
ω_9	1274	1273	1268	1282	1278	1279	1279	1278
ω_{10}	1063	1059	1060	1067	1065	1066	1067	1066
ω_{11}	939	935	938	932	931	931	933	933
ω_{12}	485	483	485	491	491	491	488	488
ω_{13}	3128	3129	3132	3105	3103	3105	3105	3104
ω_{14}	1469	1452	1455	1492	1492	1494	1492	1493
ω_{15}	1123	1126	1122	1135	1127	1129	1125	1124
ω_{16}	1070	1064	1066	1086	1082	1082	1082	1082
ω_{17}	680	677	680	686	684	684	682	681
ω_{18}	194	190	191	193	190	188	189	189
MD	3.2	0.6	0.5	2.2	-0.1	0.7	0.2	-0.7
MAD	13.3	16.6	15.8	4.0	3.0	3.1	1.6	2.1
Max Pos	43.4	43.1	49.4	12.7	5.5	7.4	3.4	3.8
Max Neg	-22.6	-37.9	-38.4	-10.6	-9.3	-9.6	-3.8	-6.4

1.7 Harmonic Infrared Intensities

Table S50. CH_2F_2 harmonic IR intensities (km mol^{-1}) obtained at the different levels of theory.

	CCSD(T)[a]	B3LYP-D3	ωB97		ωB97X		ωB97XD	
CH_2F_2	aug-cc-pCVQZ	SNSD	jul-cc-pVDZ	aug-cc-pVDZ	jul-cc-pVDZ	aug-cc-pVDZ	jul-cc-pVDZ	aug-cc-pVDZ
ω_1	37.81	40.17	35.65	35.93	37.98	38.31	40.87	41.60
ω_2	0.27	0.18	0.33	0.08	0.28	0.01	0.33	0.07
ω_3	97.11	99.74	101.55	101.47	102.31	102.35	102.52	102.63
ω_4	5.18	4.73	5.10	5.12	5.07	5.09	4.90	4.89
ω_6	26.60	28.82	22.43	22.70	24.92	25.13	27.85	28.37
ω_7	16.70	15.11	15.06	15.40	15.26	15.64	15.32	15.72
ω_8	13.40	10.26	14.55	12.38	13.28	11.17	12.46	10.56
ω_9	244.78	262.40	265.89	266.36	264.12	264.81	263.46	264.37
MD		2.45	2.34	2.20	2.67	2.58	3.23	3.29
MAD		3.76	4.35	4.29	3.51	3.86	3.88	4.37
Max Pos		17.62	21.11	21.58	19.34	20.03	18.68	19.59
Max Neg		-3.14	-4.17	-3.90	-1.68	-2.23	-1.38	-2.84

	PW6B95-D3			B2PLYP-D3			rev-DSD-PBEP86-D3	
CH_2F_2	jul-cc-pVDZ	aug-cc-pVDZ	jun-cc-pVDZ	VTZ	augVTZ	may'VTZ	jun-cc-pVTZ	aug-cc-pVTZ
ω_1	39.32	40.00	40.89	45.16	39.58	41.88	40.40	40.45
ω_2	0.49	0.09	0.79	0.88	0.22	0.45	0.28	0.29
ω_3	101.28	101.08	115.83	99.80	98.82	104.36	99.14	99.15
ω_4	5.07	5.08	5.61	4.63	4.81	4.97	4.99	4.96
ω_6	27.09	27.18	30.08	41.80	27.45	31.22	29.14	28.97
ω_7	15.16	15.45	17.61	19.06	16.02	16.48	16.61	16.73
ω_8	12.14	10.32	23.77	17.86	11.61	13.92	13.00	12.95
ω_9	259.45	260.20	291.74	237.13	258.58	269.13	251.30	251.01
MD	2.27	2.19	10.56	3.06	1.90	5.07	1.63	1.58
MAD	3.00	3.35	10.56	5.11	2.63	5.18	1.80	1.75
Max Pos	14.67	15.42	46.96	15.20	13.80	24.35	6.52	6.23
Max Neg	-1.54	-3.08	0.43	-7.65	-1.79	-0.22	-0.40	-0.45

[a] a.e.-CCSD(T)/aug-cc-pCVQZ level. From Ref. (Tasinato et al., 2012b).

Table S51. CH_2ClF harmonic IR intensities (km mol^{-1}) obtained at the different levels of theory.

CH_2ClF	CCSD(T)[a] best	B3LYP-D3 SNSD	ωB97 jul-cc-pVDZ	ωB97 aug-cc-pVDZ	ωB97X jul-cc-pVDZ	ωB97X aug-cc-pVDZ	ωB97XD jul-cc-pVDZ	ωB97XD aug-cc-pVDZ
ω_1	17.06	18.82	18.04	18.54	18.41	18.91	18.98	19.63
ω_2	0.15	0.30	0.25	0.45	0.33	0.52	0.33	0.50
ω_3	29.16	28.86	30.07	29.31	28.98	28.32	28.61	28.09
ω_4	165.57	177.06	172.47	172.71	172.73	173.18	174.29	175.08
ω_5	84.54	101.60	87.85	88.15	92.13	92.42	96.65	97.00
ω_6	1.50	1.02	2.00	1.98	1.66	1.64	1.31	1.29
ω_7	3.01	4.64	3.25	3.53	3.49	3.77	3.95	4.30
ω_8	2.47	2.43	2.20	2.15	2.33	2.27	2.46	2.39
ω_9	0.38	0.32	0.37	0.42	0.28	0.34	0.21	0.26
MD		3.47	1.41	1.49	1.83	1.95	2.55	2.74
MAD		3.66	1.47	1.56	1.93	2.19	2.75	3.07
Max Pos		17.06	6.90	7.14	7.59	7.88	12.11	12.46
Max Neg		-0.48	-0.27	-0.32	-0.18	-0.84	-0.55	-1.07

CH_2ClF	PW6B95-D3 jul-cc-pVDZ	PW6B95-D3 aug-cc-pVDZ	PW6B95-D3 jun-cc-pVDZ	B2PLYP-D3 VTZ	B2PLYP-D3 augVTZ	B2PLYP-D3 may'VTZ	rev-DSD-PBEP86-D3 jun-cc-pVTZ	rev-DSD-PBEP86-D3 aug-cc-pVTZ
ω_1	18.55	19.30	20.01	22.91	18.56	20.12	19.56	19.62
ω_2	0.17	0.40	0.35	0.04	0.19	0.13	0.21	0.16
ω_3	27.73	27.22	41.79	35.14	30.18	34.18	31.15	31.05
ω_4	172.39	172.99	200.28	167.15	169.90	178.26	165.24	164.93
ω_5	96.09	96.26	104.56	96.44	98.16	99.81	90.77	90.75
ω_6	1.36	1.34	1.46	1.13	1.13	1.15	1.31	1.29
ω_7	4.05	4.37	4.89	8.74	4.12	5.59	4.81	4.74
ω_8	2.38	2.31	3.08	2.61	2.40	2.67	2.50	2.43
ω_9	0.21	0.26	0.23	0.58	0.40	0.30	0.45	0.46
MD	2.12	2.29	8.09	3.43	2.36	4.26	1.35	1.29
MAD	2.53	2.82	8.13	3.54	2.45	4.37	1.47	1.49
Max Pos	11.55	11.72	34.71	11.90	13.62	15.27	6.23	6.21
Max Neg	-1.43	-1.94	-0.15	-0.37	-0.37	-0.35	-0.33	-0.64

[a] CCSD(T)/CBS + Δ(CV) + Δ(aug), from Ref. (Pietropolli Charmet et al., 2013)

Table S52. *cis*-ClHC=CHF harmonic IR intensities (km mol^{-1}) obtained at the different levels of theory.

cis-ClHC=CHF	Cheap[a]	B3LYP-D3 SNSD	ωB97 jul-cc-pVDZ	ωB97 aug-cc-pVDZ	ωB97X jul-cc-pVDZ	ωB97X aug-cc-pVDZ	ωB97XD jul-cc-pVDZ	ωB97XD aug-cc-pVDZ
ω_1	3.64	5.81	5.57	5.64	5.65	5.72	6.17	6.32
ω_2	8.99	7.06	7.91	7.72	7.33	7.20	6.46	6.32
ω_3	57.46	64.20	61.17	61.64	62.25	62.70	64.28	64.73
ω_4	21.88	22.75	24.09	23.55	23.13	22.55	22.72	22.19
ω_5	35.05	34.84	34.37	34.56	36.09	36.45	36.90	37.50
ω_6	79.75	99.39	97.32	98.05	96.25	96.86	96.54	96.84
ω_7	16.64	18.76	22.46	22.39	21.17	21.09	19.73	19.63
ω_8	19.91	25.70	19.21	19.38	20.99	21.15	22.92	23.07
ω_9	1.51	1.41	1.55	1.56	1.50	1.51	1.42	1.42
ω_{10}	0.76	0.37	0.09	0.21	0.12	0.27	0.18	0.41
ω_{11}	42.50	46.36	45.16	45.86	46.16	46.88	46.87	47.42
ω_{12}	6.25	6.96	6.18	6.43	6.50	6.74	6.75	6.96
MD		3.27	2.56	2.72	2.73	2.90	3.05	3.21
MAD		3.71	3.10	3.19	3.12	3.28	3.58	3.73
Max Pos		19.64	17.57	18.29	16.50	17.11	16.79	17.09
Max Neg		-1.92	-1.08	-1.26	-1.66	-1.79	-2.53	-2.67

cis-ClHC=CHF	PW6B95-D3 jul-cc-pVDZ	PW6B95-D3 aug-cc-pVDZ	PW6B95-D3 jun-cc-pVDZ	B2PLYP-D3 VTZ	B2PLYP-D3 augVTZ	B2PLYP-D3 may'VTZ	rev-DSD-PBEP86-D3 jun-cc-pVTZ	rev-DSD-PBEP86-D3 aug-cc-pVTZ
ω_1	4.84	4.77	3.98	7.17	5.41	5.76	5.56	5.48
ω_2	7.99	7.97	9.22	5.40	7.08	7.28	6.56	6.74
ω_3	63.02	63.61	69.59	59.99	62.18	63.81	61.75	61.61
ω_4	23.35	22.58	26.67	21.52	22.04	22.12	22.01	21.86
ω_5	37.36	38.04	42.41	35.30	34.58	36.11	35.41	35.81
ω_6	91.86	92.21	106.02	89.00	92.45	97.34	85.99	85.60
ω_7	20.33	20.24	22.85	16.37	17.21	17.61	16.99	16.96
ω_8	21.59	21.71	21.64	23.19	23.28	22.62	21.19	21.14
ω_9	1.45	1.44	1.50	1.34	1.40	1.43	1.45	1.45
ω_{10}	0.09	0.27	0.17	0.20	0.47	0.36	0.50	0.53
ω_{11}	44.78	45.32	46.91	43.30	44.53	47.87	43.96	44.01
ω_{12}	6.49	6.68	7.88	7.41	6.87	7.25	6.88	6.92
MD	2.40	2.54	5.38	1.32	1.93	2.94	1.16	1.15
MAD	2.69	2.80	5.47	2.14	2.39	3.30	1.62	1.57
Max Pos	12.11	12.45	26.27	9.25	12.70	17.59	6.24	5.85
Max Neg	-1.00	-1.02	-0.58	-3.59	-1.91	-1.71	-2.43	-2.25

[a] Cheap scheme, from Ref. (Boussessi et al., 2020).

Table S53. ClFC=CH_2 harmonic IR intensities (km mol^{-1}) obtained at the different levels of theory.

ClFC=CH_2	CCSD(T)[a] CVTZ+CVTZ(F)	B3LYP-D3 SNSD	ωB97 jul-cc-pVDZ	ωB97 aug-cc-pVDZ	ωB97X jul-cc-pVDZ	ωB97X aug-cc-pVDZ	ωB97XD jul-cc-pVDZ	ωB97XD aug-cc-pVDZ
ω_1	0.23	0.42	0.43	0.36	0.39	0.33	0.29	0.23
ω_2	3.11	4.53	5.95	5.53	5.32	4.96	4.43	4.12
ω_3	125.37	161.35	163.09	164.50	163.39	164.79	162.55	163.72
ω_4	2.49	1.64	0.55	0.64	0.80	0.91	1.03	1.17
ω_5	175.98	187.95	194.33	190.69	192.74	189.15	191.69	188.74
ω_6	39.91	49.64	41.71	43.43	42.25	43.89	43.53	44.90
ω_7	36.93	47.94	39.10	39.54	41.44	41.90	44.44	44.85
ω_8	1.86	0.99	1.97	1.98	1.68	1.68	1.25	1.25
ω_9	0.07	0.04	0.02	0.02	0.02	0.02	0.03	0.03
ω_{10}	54.84	57.23	58.73	58.23	59.80	59.18	60.45	59.80
ω_{11}	0.06	0.11	0.00	0.01	0.00	0.02	0.00	0.02
ω_{12}	0.78	2.04	2.27	2.24	2.46	2.41	2.52	2.45
MD		6.02	5.54	5.46	5.72	5.63	5.88	5.80
MAD		6.31	5.88	5.79	6.05	5.94	6.24	6.14
Max Pos		35.98	37.72	39.13	38.02	39.42	37.18	38.35
Max Neg		-0.87	-1.94	-1.85	-1.69	-1.58	-1.46	-1.32

ClFC=CH_2	PW6B95-D3 jul-cc-pVDZ	PW6B95-D3 aug-cc-pVDZ	PW6B95-D3 jun-cc-pVDZ	B2PLYP-D3 VTZ	B2PLYP-D3 augVTZ	B2PLYP-D3 may'VTZ	rev-DSD-PBEP86-D3 jun-cc-pVTZ	rev-DSD-PBEP86-D3 aug-cc-pVTZ
ω_1	0.42	0.33	1.01	0.38	0.54	0.47	0.33	0.35
ω_2	5.39	4.89	6.72	3.94	4.34	4.43	3.78	3.73
ω_3	159.40	160.32	170.87	155.47	153.16	156.83	144.54	144.37
ω_4	1.04	1.20	2.23	2.72	2.28	2.64	2.35	2.32
ω_5	193.28	189.44	225.89	182.93	179.10	191.34	177.53	176.93
ω_6	41.84	43.57	45.44	42.06	47.64	48.04	42.37	42.42
ω_7	42.83	43.25	47.08	45.62	43.98	44.33	40.22	40.17
ω_8	1.59	1.59	1.55	1.10	1.16	1.23	1.49	1.49
ω_9	0.01	0.01	0.03	0.05	0.02	0.02	0.01	0.01
ω_{10}	57.89	56.87	54.02	59.59	56.29	61.09	55.48	55.66
ω_{11}	0.04	0.08	0.13	0.15	0.10	0.08	0.08	0.11
ω_{12}	2.20	2.13	1.89	1.02	1.98	1.95	1.87	1.86
MD	5.36	5.17	9.60	4.45	4.08	5.90	2.37	2.32
MAD	5.66	5.44	9.84	4.58	4.24	6.01	2.46	2.42
Max Pos	34.03	34.95	49.91	30.10	27.79	31.46	19.17	19.00
Max Neg	-1.45	-1.29	-0.82	-0.76	-0.70	-0.63	-0.37	-0.37

[a] a.e.-CCSD(T)/cc-p CVTZ/aug-cc-pCVTZ(F), from Ref. (Pietropolli Charmet et al., 2016).

Table S54. $ClFC{=}CF_2$ harmonic IR intensities (km mol^{-1}) obtained at the different levels of theory.

$ClFC{=}CF_2$	CCSD(T)[a]	B3LYP-D3	ωB97		ωB97X		ωB97XD	
	ANO2	SNSD	jul-cc-pVDZ	aug-cc-pVDZ	jul-cc-pVDZ	aug-cc-pVDZ	jul-cc-pVDZ	aug-cc-pVDZ
ω_1	59.27	60.33	60.44	60.44	62.91	62.91	64.96	64.96
ω_2	159.46	189.61	180.19	180.19	180.97	180.97	183.52	183.52
ω_3	187.55	211.17	207.83	207.83	209.64	209.64	211.82	211.82
ω_4	218.46	241.99	256.67	256.67	251.88	251.88	246.73	246.73
ω_5	2.89	3.79	1.70	1.70	2.30	2.30	3.07	3.07
ω_6	1.22	1.11	0.82	0.82	0.90	0.90	0.98	0.98
ω_7	0.97	0.48	1.18	1.18	0.93	0.93	0.67	0.67
ω_8	1.39	1.40	1.23	1.23	1.30	1.30	1.36	1.36
ω_9	2.53	2.61	2.61	2.61	2.60	2.60	2.54	2.54
ω_{10}	2.23	1.87	2.37	2.37	2.40	2.40	2.33	2.33
ω_{11}	1.41	1.19	1.63	1.63	1.43	1.43	1.15	1.15
ω_{12}	0.05	0.05	0.05	0.05	0.05	0.05	0.05	0.05
MD		6.51	6.61	6.61	6.66	6.66	6.81	6.81
MAD		6.71	6.90	6.90	6.83	6.83	6.95	6.95
Max Pos		30.15	38.21	38.21	33.42	33.42	28.27	28.27
Max Neg		-0.49	-1.19	-1.19	-0.59	-0.59	-0.30	-0.30

$ClFC{=}CF_2$	PW6B95-D3			B2PLYP-D3			rev-DSD-PBEP86-D3	
	jul-cc-pVDZ	aug-cc-pVDZ	jun-cc-pVDZ	VTZ	augVTZ	may'VTZ	jun-cc-pVTZ	aug-cc-pVTZ
ω_1	62.70	62.70	65.33	61.38	63.36	66.55	63.60	63.64
ω_2	187.17	187.17	214.95	172.62	183.38	189.98	176.67	177.97
ω_3	204.65	204.65	238.22	207.17	200.63	212.95	198.11	196.05
ω_4	245.26	245.26	268.73	233.68	233.04	239.84	228.72	228.29
ω_5	2.77	2.77	3.15	3.66	3.35	3.24	2.91	2.85
ω_6	1.08	1.08	1.30	1.00	1.06	1.10	1.07	1.07
ω_7	0.93	0.93	0.83	0.44	0.62	0.65	0.82	0.83
ω_8	1.31	1.31	1.46	1.35	1.34	1.38	1.33	1.33
ω_9	2.66	2.66	3.08	2.59	2.55	2.67	2.54	2.54
ω_{10}	2.20	2.20	2.65	2.05	1.92	2.03	1.94	1.96
ω_{11}	1.34	1.34	1.74	0.89	1.08	1.17	1.09	1.07
ω_{12}	0.06	0.06	0.08	0.05	0.05	0.06	0.05	0.05
MD	6.22	6.22	13.67	4.12	4.58	7.02	3.45	3.35
MAD	6.30	6.30	13.70	4.37	4.78	7.16	3.61	3.52
Max Pos	27.71	27.71	55.49	19.62	23.92	30.52	17.21	18.51
Max Neg	-0.14	-0.14	-0.14	-0.53	-0.35	-0.32	-0.32	-0.34

[a] CCSD(T)/ANOtz, from Ref. (Tasinato et al., 2012a).

Table S55. Oxirane harmonic IR intensities (km mol^{-1}) obtained at the different levels of theory.

Oxirane	CCSD(T)[a] CBS+CV+aug	B3LYP-D3 SNSD (PES1)	ωB97 jul-cc-pVDZ	ωB97 aug-cc-pVDZ	ωB97X jul-cc-pVDZ	ωB97X aug-cc-pVDZ	ωB97XD jul-cc-pVDZ	ωB97XD aug-cc-pVDZ
ω_1	15.05	17.78	15.35	15.71	15.71	16.10	16.68	17.05
ω_2	1.89	3.02	4.34	3.89	3.84	3.37	3.35	2.93
ω_3	12.85	16.65	12.94	14.15	14.29	15.61	15.12	16.39
ω_4	0.01	0.00	0.93	0.70	0.32	0.19	0.03	0.01
ω_5	71.34	71.88	76.74	76.98	76.69	76.74	75.97	75.76
ω_9	25.24	41.53	34.20	34.08	36.57	36.34	40.64	40.71
ω_{10}	4.37	4.01	4.28	4.59	4.20	4.51	4.07	4.38
ω_{11}	0.30	0.13	0.38	0.26	0.39	0.27	0.37	0.24
ω_{12}	26.06	37.59	31.01	31.95	33.10	33.90	36.44	37.65
ω_{13}	-0.04	0.07	0.61	0.68	0.59	0.68	0.40	0.45
ω_{14}	0.36	0.17	0.30	0.23	0.29	0.22	0.21	0.16
ω_{15}	8.02	11.07	11.10	10.92	10.43	10.29	10.14	10.15
MD		3.21	2.23	2.39	2.58	2.73	3.17	3.37
MAD		3.33	2.25	2.42	2.62	2.76	3.24	3.41
Max Pos		16.29	8.96	8.84	11.33	11.10	15.41	15.47
Max Neg		-0.36	-0.09	-0.13	-0.17	-0.14	-0.30	-0.20

Oxirane	PW6B95-D3 jul-cc-pVDZ	PW6B95-D3 aug-cc-pVDZ	PW6B95-D3 jun-cc-pVDZ	B2PYLP VTZ (PES5)	B2PLYP-D3 augVTZ (PES4)	B2PLYP-D3 may'VTZ (PES2)	rev-DSD-PBEP86-D3 jun-cc-pVTZ	rev-DSD-PBEP86-D3 aug-cc-pVTZ
ω_1	16.13	16.70	13.32	14.21	16.04	16.03	15.41	15.54
ω_2	3.53	3.01	6.24	2.15	2.09	2.56	2.04	2.07
ω_3	14.30	15.68	15.02	12.38	13.37	13.59	12.68	12.74
ω_4	0.01	0	0.51	0.02	0.05	0.02	0.01	0.01
ω_5	71.72	71.38	77.40	64.17	71.33	73.59	70.86	71.11
ω_9	39.05	38.93	37.09	43.79	34.44	37.26	34.42	34.53
ω_{10}	3.70	3.99	3.35	3.38	3.90	3.38	4.14	4.18
ω_{11}	0.27	0.17	0.31	0.01	0.25	0.21	0.25	0.24
ω_{12}	33.49	34.70	29.31	38.24	32.69	33.68	32.00	32.35
ω_{13}	0.15	0.23	0.09	0.03	0.08	0.02	0.10	0.10
ω_{14}	0.13	0.09	0.20	1.50	0.28	0.60	0.23	0.21
ω_{15}	10.30	10.30	11.54	10.42	9.55	9.88	8.92	9.08
MD	2.28	2.48	2.41	2.07	1.55	2.12	1.30	1.39
MAD	2.43	2.61	2.90	3.70	1.65	2.30	1.48	1.52
Max Pos	13.81	13.69	11.86	18.55	9.20	12.02	9.19	9.30
Max Neg	-0.67	-0.37	-1.73	-7.17	-0.47	-0.99	-0.48	-0.23

[a] CCSD(T)/CBS + Δ(CV) + Δ(aug), from Ref. (Puzzarini et al., 2014a).

Table S56. CH_3CH_2SH harmonic IR intensities (km mol^{-1}) obtained at the different levels of theory.

	CCSD[a]	B3LYP-D3	ωB97		ωB97X		ωB97XD	
CH_3CH_2SH	VTZ	SNSD	jul-cc-pVDZ	aug-cc-pVDZ	jul-cc-pVDZ	aug-cc-pVDZ	jul-cc-pVDZ	aug-cc-pVDZ
ω_1	30.20	25.69	22.87	22.71	24.44	24.12	26.27	24.97
ω_2	34.10	3.44	20.53	16.11	19.74	13.99	25.71	24.88
ω_3	0.60	29.23	5.86	9.69	8.24	13.32	5.72	3.53
ω_4	8.20	10.15	14.23	14.88	13.99	14.51	15.31	13.51
ω_5	6.80	27.44	22.68	22.12	24.08	23.35	26.48	26.44
ω_6	5.40	4.46	1.04	1.23	1.62	1.91	2.26	2.65
ω_7	2.40	2.67	3.35	3.36	3.33	3.38	3.00	2.96
ω_8	15.70	9.54	9.69	10.83	9.74	10.95	9.41	10.54
ω_9	102.30	1.06	1.49	1.41	1.59	1.54	1.52	1.60
ω_{10}	1.10	3.44	6.17	6.65	5.79	6.31	4.94	5.39
ω_{11}	43.90	22.02	15.00	15.16	15.86	15.94	16.65	18.80
ω_{12}	5.90	3.20	2.05	2.31	2.18	2.46	2.26	3.27
ω_{13}	5.30	5.57	5.58	5.25	5.90	5.53	6.23	5.22
ω_{14}	0	0.24	0.92	0.78	0.71	0.59	0.37	0.82
ω_{15}	0	7.70	5.35	5.07	5.79	5.54	6.13	7.01
ω_{16}	3.30	4.88	6.14	5.79	6.06	5.68	5.94	5.40
ω_{17}	1.20	1.44	1.57	1.55	1.72	1.69	1.74	1.75
ω_{18}	2.90	4.60	2.63	2.49	2.73	2.59	2.68	4.11
ω_{19}	1	1.08	1.16	1.14	1.03	1.02	0.72	1.39
ω_{20}	2.60	0.98	1.62	1.22	1.61	1.12	0.67	2.36
ω_{21}	13.50	12.78	16.21	15.66	15.58	15.12	16.31	12.32
MD		-4.99	-5.73	-5.76	-5.46	-5.51	-5.05	-5.12
MAD		11.24	10.09	10.39	10.11	10.52	9.79	9.39
Max Pos		28.63	15.88	15.32	17.28	16.55	19.68	19.64
Max Neg		-101.24	-100.81	-100.89	-100.71	-100.76	-100.78	-100.70

[a] from Ref. (Hochlaf et al., 2015).

	PW6B95-D3			B2PLYP-D3			rev-DSD-PBEP86-D3	
CH_3CH_2SH	jul-cc-pVDZ	aug-cc-pVDZ	jun-cc-pVDZ	VTZ	augVTZ	may'VTZ	jun-cc-pVTZ	aug-cc-pVTZ
ω_1	24.90	24.93	20.32	26.51	23.05	24.32	23.78	23.72
ω_2	19.25	13.86	17.78	9.31	7.26	12.09	12.88	12.42
ω_3	10.51	15.64	7.85	20.93	21.38	17.20	16.08	16.58
ω_4	13.57	14.44	14.74	12.53	11.16	11.89	12.42	12.37
ω_5	25.84	25.92	25.79	25.25	24.73	25.23	24.24	24.19
ω_6	2.28	2.90	4.09	4.24	2.58	3.17	2.17	2.29
ω_7	2.44	2.46	3.72	2.46	2.51	2.81	2.44	2.39
ω_8	8.64	9.89	11.84	9.12	9.53	9.59	9.45	9.36
ω_9	1.13	1.18	1.45	1.29	1.26	1.39	1.28	1.30
ω_{10}	3.97	4.54	3.52	3.30	3.52	3.73	3.57	3.61
ω_{11}	18.56	18.37	23.50	19.58	19.58	20.11	18.02	17.76
ω_{12}	2.95	3.35	3.90	2.77	2.72	2.94	2.28	2.32
ω_{13}	5.81	5.52	8.31	6.24	6.32	7.62	6.56	6.53
ω_{14}	1.18	1.03	1.52	0.20	0.23	0.32	0.28	0.29
ω_{15}	6.58	6.38	6.89	6.28	6.43	6.10	5.34	5.21
ω_{16}	5.42	5.00	7.67	5.59	5.20	5.82	5.16	5.09
ω_{17}	1.73	1.65	2.17	1.52	1.44	1.57	1.37	1.39
ω_{18}	3.28	3.17	3.29	3.27	3.41	3.10	2.63	2.62
ω_{19}	1.24	1.27	1.86	1.39	1.11	1.25	1.15	1.13
ω_{20}	1.80	1.06	1.48	1.51	1.04	1.34	1.19	1.16
ω_{21}	12.68	12.51	16.91	13.85	12.58	13.82	12.39	12.41
MD	-5.36	-5.30	-4.66	-5.20	-5.69	-5.29	-5.80	-5.82
MAD	10.01	10.47	10.17	10.59	10.89	10.44	10.44	10.48
Max Pos	19.04	19.12	18.99	20.33	20.78	18.43	17.44	17.39
Max Neg	-101.17	-101.12	-100.85	-101.01	-101.04	-100.91	-101.02	-101.00

Table S57. SO_2 harmonic IR intensities (km mol^{-1}) obtained at the different levels of theory.

SO_2	Cheap[a]	B3LYP-D3 SNSD	ωB97 jul-cc-pVDZ	ωB97 aug-cc-pVDZ	ωB97X jul-cc-pVDZ	ωB97X aug-cc-pVDZ	ωB97XD jul-cc-pVDZ	ωB97XD aug-cc-pVDZ
ω_1	22.82	24.95	34.47	34.47	33.89	33.89	32.36	32.36
ω_2	28.08	27.02	31.98	31.98	31.83	31.83	30.83	30.83
ω_3	176.82	205.54	248.88	248.88	242.90	242.90	233.70	233.70
MD		9.93	29.21	29.21	26.97	26.97	23.06	23.06
MAD		10.64	29.21	29.21	26.97	26.97	23.06	23.06
Max Pos		28.73	72.06	72.06	66.08	66.08	56.88	56.88
Max Neg		-1.06	3.90	3.90	3.76	3.76	2.76	2.76

SO_2	PW6B95-D3 jul-cc-pVDZ	PW6B95-D3 aug-cc-pVDZ	PW6B95-D3 jun-cc-pVDZ	B2PLYP-D3 VTZ	B2PLYP-D3 augVTZ	B2PLYP-D3 may'VTZ	rev-DSD-PBEP86-D3 jun-cc-pVTZ	rev-DSD-PBEP86-D3 aug-cc-pVTZ
ω_1	30.68	29.54	39.75	20.44	20.81	25.50	23.47	23.39
ω_2	30.01	28.84	36.95	27.64	26.20	28.85	27.40	27.28
ω_3	223.25	217.22	250.22	156.07	176.63	188.88	181.93	181.65
MD	18.74	15.96	33.07	-7.86	-1.36	5.17	1.70	1.53
MAD	18.74	15.96	33.07	7.86	1.36	5.17	2.14	2.07
Max Pos	46.43	40.40	73.40	-0.44	-0.19	12.06	5.11	4.83
Max Neg	1.93	0.76	8.87	-20.75	-2.01	0.77	-0.67	-0.80

[a] Cheap scheme, from Ref. (Boussessi et al., 2020).

Table S58. $HCOCH_2OH$ harmonic IR intensities (km mol^{-1}) obtained at the different levels of theory.

$HCOCH_2OH$	cheap[a]	B3LYP-D3 SNSD	ωB97 jul-cc-pVDZ	ωB97 aug-cc-pVDZ	ωB97X jul-cc-pVDZ	ωB97X aug-cc-pVDZ	ωB97XD jul-cc-pVDZ	ωB97XD aug-cc-pVDZ
ω_1	68.38	66.15	79.37	77.19	77.03	74.69	72.53	69.74
ω_2	60.30	68.65	56.46	56.86	60.10	60.51	64.63	65.76
ω_3	38.09	55.33	46.55	47.65	48.41	49.53	53.65	54.54
ω_4	120.87	160.27	161.67	160.12	162.24	160.66	162.54	161.15
ω_5	16.75	20.68	26.47	27.13	24.47	24.48	25.85	25.73
ω_6	36.41	40.26	34.30	32.39	36.98	35.37	37.82	35.96
ω_7	14.96	22.43	23.21	24.20	24.26	25.61	26.29	28.10
ω_8	44.21	44.59	44.70	43.23	44.71	43.35	41.69	40.42
ω_9	77.35	83.55	84.46	83.62	86.12	85.51	85.93	85.55
ω_{10}	44.05	49.81	43.23	42.68	44.91	44.38	47.02	46.56
ω_{11}	11.83	10.26	13.21	12.98	12.61	12.33	11.60	11.28
ω_{12}	20.51	22.99	23.00	22.53	23.35	22.94	22.98	22.62
ω_{13}	12.02	16.42	13.68	14.17	14.78	15.26	16.33	16.93
ω_{14}	1.69	2.09	1.24	1.46	1.40	1.62	1.52	1.76
ω_{15}	0.51	0.25	0.12	0.21	0.14	0.25	0.16	0.29
ω_{16}	0.21	0.01	0.14	0.07	0.13	0.06	0.09	0.04
ω_{17}	88.63	85.70	94.27	90.04	94.25	89.94	92.62	88.48
ω_{18}	2.89	2.99	3.92	3.51	3.90	3.54	4.03	3.80
MD		5.15	5.02	4.47	5.56	5.02	5.98	5.50
MAD		5.95	5.87	5.63	5.67	5.28	6.35	6.09
Max Pos		39.40	40.80	39.26	41.37	39.79	41.67	40.28
Max Neg		-2.93	-3.84	-4.02	-0.37	-1.03	-2.52	-3.79

[a] Cheap scheme, from Ref. (Boussessi et al., 2020).

$HCOCH_2OH$	PW6B95-D3			B2PLYP-D3			rev-DSD-PBEP86-D3	
	jul-cc-pVDZ	aug-cc-pVDZ	jun-cc-pVDZ	VTZ	augVTZ	may'VTZ	jun-cc-pVTZ	aug-cc-pVTZ
ω_1	73.83	71.60	84.70	64.51	67.28	68.13	67.64	66.75
ω_2	67.34	68.46	68.02	61.77	61.64	61.07	63.49	63.37
ω_3	50.56	51.06	47.86	64.43	56.92	60.17	51.45	52.00
ω_4	159.87	158.10	181.13	123.13	133.09	136.63	122.87	123.02
ω_5	23.71	24.62	31.81	16.55	17.88	17.94	16.09	15.77
ω_6	35.87	32.95	33.40	55.50	41.59	43.35	41.76	42.79
ω_7	28.92	31.34	30.69	18.11	18.50	20.29	17.72	16.68
ω_8	41.18	39.74	29.49	47.67	47.17	44.08	45.48	45.77
ω_9	88.20	87.39	94.84	76.83	79.14	80.36	78.39	78.42
ω_{10}	46.51	45.94	50.82	50.33	50.13	51.86	47.37	47.31
ω_{11}	11.09	10.81	10.13	10.24	10.55	10.31	11.27	11.29
ω_{12}	23.26	22.71	23.51	23.35	22.12	22.74	21.79	21.65
ω_{13}	15.29	15.83	15.24	20.85	14.84	16.05	16.00	16.04
ω_{14}	1.74	1.98	2.62	1.98	1.87	2.19	1.77	1.80
ω_{15}	0.11	0.27	0.09	0.34	0.32	0.34	0.54	0.52
ω_{16}	0.06	0.02	0.02	0.01	0.02	0.00	0.02	0.03
ω_{17}	89.63	85.58	100.49	82.93	86.39	92.76	86.30	86.06
ω_{18}	3.23	3.01	6.17	3.00	3.16	4.55	4.01	3.55
MD	5.60	5.10	8.41	3.44	2.94	4.07	1.91	1.84
MAD	6.14	6.48	10.63	4.80	3.50	4.32	2.40	2.50
Max Pos	39.00	37.23	60.26	26.34	18.83	22.08	13.36	13.91
Max Neg	-3.03	-4.47	-14.72	-5.70	-2.24	-1.52	-2.33	-2.57

Table S59. *E*-Ethanimine harmonic IR intensities (km mol^{-1}) obtained at the different levels of theory.

	CCSD(T)^a	B3LYP-D3	ωB97		ωB97X		ωB97XD	
E-Ethanimine	pCVQZ	SNSD	jul-cc-pVDZ	aug-cc-pVDZ	jul-cc-pVDZ	aug-cc-pVDZ	jul-cc-pVDZ	aug-cc-pVDZ
ω_1	0.72	0.99	1.45	1.85	1.10	1.46	0.71	1.06
ω_2	9.12	11.62	8.97	8.72	9.86	9.57	11.22	11.00
ω_3	29.63	14.63	18.51	21.77	16.40	18.16	15.37	16.92
ω_4	28.37	56.79	39.77	36.50	45.20	43.27	51.97	50.50
ω_5	50.68	83.95	86.97	87.64	86.27	86.86	84.74	85.47
ω_6	11.40	16.93	13.17	8.96	14.30	11.57	15.29	14.28
ω_7	23.14	19.86	23.31	29.82	22.75	27.96	20.75	23.95
ω_8	10.94	10.99	11.15	9.58	11.81	10.27	11.50	9.97
ω_9	26.66	27.20	22.76	25.25	23.60	26.27	25.50	28.16
ω_{10}	29.88	29.72	27.30	27.47	27.89	28.03	27.82	27.86
ω_{11}	5.44	7.50	4.79	4.59	5.61	5.38	6.70	6.47
ω_{12}	17.67	17.92	19.57	19.46	19.51	19.34	19.11	18.90
ω_{13}	12.09	12.94	11.18	11.01	11.92	11.69	12.96	12.83
ω_{14}	8.00	8.77	9.29	9.89	9.39	10.06	9.15	9.71
ω_{15}	3.29	2.25	3.37	3.74	3.23	3.62	3.09	3.40
ω_{16}	13.13	14.38	12.98	13.04	13.18	13.24	13.16	13.26
ω_{17}	51.02	53.17	54.43	53.75	55.55	54.84	55.98	55.12
ω_{18}	1.95	1.99	2.26	2.26	2.21	2.19	2.08	2.05
MD		3.25	2.12	2.34	2.59	2.81	3.00	3.21
MAD		5.41	4.28	4.33	4.69	4.46	5.23	4.95
Max Pos		33.26	36.29	36.96	35.59	36.18	34.06	34.78
Max Neg		-15.00	-11.12	-7.86	-13.23	-11.46	-14.26	-12.71

[a] a.e.-CCSD(T)/cc-pCVQZ, from Ref. (Melli et al., 2018).

	PW6B95-D3			B2PLYP-D3			rev-DSD-PBEP86-D3	
E-Ethanimine	jul-cc-pVDZ	aug-cc-pVDZ	jun-cc-pVDZ	VTZ	augVTZ	may'VTZ	jun-cc-pVTZ	aug-cc-pVTZ
ω_1	0.71	1.00	0.51	0.63	1.01	0.98	0.98	1.07
ω_2	10.28	10.29	7.58	10.70	9.76	9.88	10.06	10.07
ω_3	12.78	13.92	11.76	13.15	14.30	13.27	23.23	21.81
ω_4	56.66	55.57	59.88	60.01	52.06	55.86	40.15	41.56
ω_5	83.89	84.66	90.28	59.58	69.75	69.29	62.93	63.37
ω_6	14.38	13.17	19.75	12.86	15.65	15.78	14.06	14.22
ω_7	20.62	24.28	16.80	23.79	19.28	18.71	19.72	19.09
ω_8	10.71	9.38	10.29	13.41	13.51	13.56	11.57	12.25
ω_9	24.43	27.49	25.19	31.42	26.88	27.17	25.55	25.33
ω_{10}	30.85	30.68	30.06	31.70	31.51	31.67	30.37	30.54
ω_{11}	5.68	5.37	5.87	7.26	7.32	7.23	5.97	6.06
ω_{12}	18.66	18.39	18.45	17.20	17.93	18.07	18.15	18.08
ω_{13}	11.67	11.81	9.28	13.78	11.64	12.36	12.45	12.43
ω_{14}	8.16	8.88	12.14	7.98	8.48	9.15	8.36	8.27
ω_{15}	3.82	4.23	5.07	4.25	1.84	2.05	1.95	1.84
ω_{16}	13.18	13.17	11.93	13.76	13.78	14.10	13.57	13.72
ω_{17}	53.04	52.10	56.91	50.36	52.75	55.32	52.32	52.54
ω_{18}	1.83	1.79	1.48	1.46	2.02	2.10	2.10	2.12
MD	2.68	2.95	3.34	2.23	2.02	2.41	1.13	1.18
MAD	5.16	4.92	6.96	4.26	4.36	4.86	2.50	2.81
Max Pos	33.21	33.97	39.60	31.65	23.70	27.49	12.25	13.19
Max Neg	-16.85	-15.71	-17.87	-16.47	-15.33	-16.36	-6.40	-7.82

1.8 Anharmonic Fundamental Frequencies

Table S60. CH_2F_2 anharmonic fundamental frequencies (cm^{-1}) obtained at the different levels of theory and comparison to experimental data.

CH_2F_2	Exp[a]	CCSD(T)[b] aug-cc-pCVQZ	B3LYP-D3 SNSD	ωB97 jul-cc-pVDZ	ωB97 aug-cc-pVDZ	ωB97X jul-cc-pVDZ	ωB97X aug-cc-pVDZ	ωB97XD jul-cc-pVDZ	ωB97XD aug-cc-pVDZ
ν_1	2948	2955	2918	2969	2967	2989	2985	2897	2887
ν_2	1510	1508	1483	1459	1455	1469	1464	1471	1467
ν_3	1112	1112	1080	1090	1090	196	1096	1093	1093
ν_4	528	529	513	508	508	513	513	514	515
ν_5	1257	1256	1232	1214	1213	1224	1224	1225	1224
ν_6	3014	3015	2994	3006	3000	3024	3018	3004	2995
ν_7	1179	1179	1153	1137	1136	1147	1144	1149	1148
ν_8	1436	1436	1413	1391	1390	1399	1400	1401	1399
ν_9	1090	1091	1040	1064	1063	168	1066	1061	1059
MD		0.8	-27.6	-26.0	-27.9	-16.1	-18.1	-28.6	-31.7
MAD		1.3	27.6	30.7	32.1	27.4	27.1	28.6	31.7
Max Pos		7.1	-15.7	21.4	18.9	40.9	37.2	-10.5	-13.5
Max Neg		-1.6	-50.2	-50.9	-54.3	-41.0	-45.9	-51.3	-60.7

CH_2F_2	PW6B95-D3 jul-cc-pVDZ	PW6B95-D3 aug-cc-pVDZ	PW6B95-D3 jun-cc-pVDZ	B2PLYP-D3 VTZ	B2PLYP-D3 augVTZ	B2PLYP-D3 may'VTZ	rev-DSD-PBEP86-D3 jun-cc-pVTZ	rev-DSD-PBEP86-D3 aug-cc-pVTZ	Hyb revDSD PW6
ν_1	3007	3009	3007	2966	2957	2960	2960	2960	2965
ν_2	1474	1470	1467	1516	1508	1509	1510	1511	1518
ν_3	1097	1097	1101	1104	1092	1095	1103	1103	1105
ν_4	513	514	522	525	520	522	525	524	526
ν_5	1224	1224	1227	1257	1249	1253	1254	1253	1254
ν_6	3047	3047	3046	3019	3025	3025	3025	3024	3033
ν_7	1150	1151	1149	1180	1171	1174	1176	1175	1181
ν_8	1398	1398	1407	1446	1432	1437	1436	1436	1435
ν_9	1066	1065	1072	1079	1054	1059	1075	1075	1076
MD	-10.8	-11.1	-8.3	2.3	-7.3	-4.4	-0.9	-1.3	2.1
MAD	31.1	31.7	28.3	6.9	11.6	9.8	6.2	6.8	8.1
Max Pos	59.0	60.7	58.6	18.4	10.6	12.5	12.3	12.5	18.6
Max Neg	-37.9	-40.0	-42.4	-10.5	-35.8	-31.1	-14.8	-14.9	-13.9

[a] From Ref. (Tasinato et al., 2012b).
[b] Geometry and quadratic force constants obtained at the CCSD(T)/aug-cc-pCVQZ level; cubic and semi-diagonal force constants evaluated at the CCSD(T) level employing the aug-cc-pCVQZ basis set for F atoms and the aug-cc-pCVTZ basis for H and C atoms. From Ref. (Tasinato et al., 2012b).

Table S61. CH_2ClF anharmonic fundamental frequencies (cm^{-1}) obtained at the different levels of theory and comparison to experimental data.

CH_2ClF	Exp[a]	CCSD(T)[b] CBS+CV+aug	B3LYP-D3 SNSD	ωB97 jul-cc-pVDZ	ωB97 aug-cc-pVDZ	ωB97X jul-cc-pVDZ	ωB97X aug-cc-pVDZ	ωB97XD jul-cc-pVDZ	ωB97XD aug-cc-pVDZ
ν_1	2993	2996	2987	3047	3044	3033	3030	3009	3004
ν_2	1474	1465	1454	1456	1451	1458	1452	1459	1453
ν_3	1353	1348	1333	1356	1355	1352	1351	1344	1343
ν_4	1068	1062	1040	1049	1049	1055	1055	1053	1053
ν_5	760	764	716	802	802	785	785	762	762
ν_6	384	384	372	388	388	387	387	383	383
ν_7	3035	3038	3031	3112	3107	3093	3089	3066	3058
ν_8	1237	1230	1217	1222	1220	1222	1221	1217	1216
ν_9	1001	1001	985	1023	1021	1017	1016	1017	1015
MD		-1.8	-19.0	16.9	14.9	11.0	9.1	0.9	-2.0
MAD		4.1	19.0	28.3	27.8	20.9	20.8	13.8	13.1
Max Pos		3.6	-4.8	76.9	71.9	58.0	53.3	31.1	22.8
Max Neg		-8.5	-43.6	-18.7	-22.4	-16.0	-21.4	-20.1	-21.6

CH_2ClF	PW6B95-D3 jul-cc-pVDZ	PW6B95-D3 aug-cc-pVDZ	PW6B95-D3 jun-cc-pVDZ	B2PLYP-D3 VTZ	B2PLYP-D3 augVTZ	B2PLYP-D3 may'VTZ	rev-DSD-PBEP86-D3 jun-cc-pVTZ	rev-DSD-PBEP86-D3 aug-cc-pVTZ	Hyb revDSD PW6
ν_1	3017	3018	3014	3018	3015	3015	3013	3013	3030
ν_2	1448	1442	1443	1482	1476	1478	1477	1479	1485
ν_3	1325	1326	1331	1358	1353	1357	1358	1358	1360
ν_4	1051	1051	1055	1067	1045	1047	1059	1058	1060
ν_5	752	751	754	736	735	739	754	754	755
ν_6	380	380	384	379	378	380	383	382	384
ν_7	3075	3075	3071	3051	3053	3050	3048	3047	3055
ν_8	1213	1212	1215	1239	1233	1237	1238	1236	1241
ν_9	990	990	986	1003	1001	1001	1005	1005	1009
MD	-5.9	-6.6	-5.7	3.1	-1.6	-0.0	3.6	3.1	8.2
MAD	20.2	21.0	18.5	9.6	11.1	10.1	6.7	7.0	11.1
Max Pos	40.0	39.4	35.5	25.6	22.6	22.2	20.5	20.0	37.3
Max Neg	-27.6	-31.9	-30.8	-24.3	-24.7	-20.6	-8.4	-9.7	-8.0

[a] From Ref. (Pietropolli Charmet et al., 2013).
[b] CCSD(T)/CBS + CV + aug harmonic frequencies augmented by the CCSD(T)/aug-cc-pVTZ cubic and quartic semi-diagonal force constants, from Ref. (Pietropolli Charmet et al., 2013).

Table S62. *cis*-ClHC=CHF anharmonic fundamental frequencies (cm^{-1}) obtained at the different levels of theory and comparison to experimental data.

cis-ClHC=CHF	Exp[a]	CCSD(T)[b]	B3LYP-D3 SNSD	ωB97 jul-cc-pVDZ	ωB97 aug-cc-pVDZ	ωB97X jul-cc-pVDZ	ωB97X aug-cc-pVDZ	ωB97XD jul-cc-pVDZ	ωB97XD aug-cc-pVDZ
ν_1	3114	3109	3106	2979	2984	3050	3052	2952	2955
ν_2	3102	3100	3094	3164	3166	3157	3158	3148	3147
ν_3	1661	1660	1672	1738	1737	1731	1730	1711	1710
ν_4	1335	1332	1315	1310	1313	1316	1318	1310	1312
ν_5	1232	1231	1222	1212	1213	1219	1220	1218	1218
ν_6	1062	1057	1047	1048	1049	1054	1055	1051	1052
ν_7	812	810	789	830	831	819	820	805	806
ν_8	656	661	642	662	662	661	662	657	657
ν_9	200	201	196	202	203	201	201	197	198
ν_{10}	857	854	855	886	896	882	891	865	874
ν_{11}	735	728	731	728	729	732	734	719	720
ν_{12}	442	438	446	461	462	458	459	455	456
MD		-2.3	-7.8	1.2	3.0	5.9	7.7	-10.1	-8.5
MAD		3.3	10.3	34.6	34.7	24.0	24.2	29.7	29.7
Max Pos		5.0	11.1	77.4	76.2	70.4	69.2	50.4	48.9
Max Neg		-7.0	-23.0	-134.6	-130.3	-64.1	-61.5	-161.7	-159.2

cis-ClHC=CHF	PW6B95-D3 jul-cc-pVDZ	PW6B95-D3 aug-cc-pVDZ	PW6B95-D3 jun-cc-pVDZ	B2PLYP-D3 VTZ	B2PLYP-D3 augVTZ	B2PLYP-D3 may'VTZ	rev-DSD-PBEP86-D3 jun-cc-pVTZ	rev-DSD-PBEP86-D3 aug-cc-pVTZ	Hyb revDSD PW6
ν_1	3149	3152	3152	3128	3124	3122	3124	3122	3130
ν_2	3143	3147	3146	3116	3115	3116	3117	3115	3125
ν_3	1707	1706	1709	1676	1668	1669	1675	1673	1676
ν_4	1317	1319	1316	1340	1333	1337	1336	1334	1340
ν_5	1224	1224	1224	1239	1233	1237	1235	1233	1236
ν_6	1057	1057	1059	1065	1056	1058	1063	1060	1061
ν_7	811	811	812	802	800	802	809	808	808
ν_8	658	659	659	654	651	654	658	657	659
ν_9	200	200	201	198	197	198	198	196	208
ν_{10}	867	877	877	874	872	874	870	871	879
ν_{11}	751	757	757	748	744	746	747	745	762
ν_{12}	451	453	452	453	451	452	451	450	451
MD	10.6	12.8	13.1	7.0	3.1	4.7	6.3	4.7	10.5
MAD	16.1	17.7	18.2	9.4	7.7	7.7	7.1	6.3	11.3
Max Pos	46.2	45.4	48.0	16.8	15.4	17.0	15.2	14.2	26.9
Max Neg	-18.2	-16.2	-19.3	-9.9	-12.5	-9.7	-2.6	-4.0	-3.9

[a] From Ref. (Craig et al., 1970).
[b] Scaled CCSD(T)/cc-pVTZ, from Ref. (Gambi et al., 2002).

Table S63. ClFC=CH_2 anharmonic fundamental frequencies (cm^{-1}) obtained at the different levels of theory and comparison to experimental data.

ClFC=CH_2	Exp[a]	CCSD(T)[b] CVTZ+CVTZ(F)	B3LYP-D3 SNSD	ωB97 jul-cc-pVDZ	ωB97 aug-cc-pVDZ	ωB97X jul-cc-pVDZ	ωB97X aug-cc-pVDZ	ωB97XD jul-cc-pVDZ	ωB97XD aug-cc-pVDZ
ν_1	3159	3160	3146	3133	3140	3115	3121	3052	3058
ν_2	3072	3072	3075	3008	3012	2996	3001	2935	2939
ν_3	1656	1654	1662	1735	1728	1730	1722	1708	1709
ν_4	1375	1377	1338	1332	1340	1336	1342	1367	1332
ν_5	1188	1185	1153	1180	1182	1180	1182	1173	1173
ν_6	948	945	934	927	930	934	935	934	934
ν_7	700	697	679	722	722	715	715	704	703
ν_8	432	430	427	432	432	433	433	432	432
ν_9	370	368	369	374	375	375	376	373	373
ν_{10}	836	835	838	851	863	846	859	823	835
ν_{11}	n.a.	709	701	707	706	700	701	686	686
ν_{12}	515	516	519	530	530	529	530	522	522
MD		-1.3	-9.9	-2.5	0.2	-5.5	-3.0	-20.8	-21.7
MAD		2.0	13.1	27.0	25.6	27.2	25.9	32.7	33.8
Max Pos		2.1	6.6	78.9	72.4	73.8	66.6	51.8	53.0
Max Neg		-3.6	-37.1	-64.3	-60.5	-75.9	-70.9	-137.3	-132.6

ClFC=CH_2	PW6B95-D3 jul-cc-pVDZ	PW6B95-D3 aug-cc-pVDZ	PW6B95-D3 aug-cc-pVDZ	B2PLYP-D3 VTZ	B2PLYP-D3 augVTZ	B2PLYP-D3 may'VTZ	rev-DSD-PBEP86-D3 jun-cc-pVTZ	rev-DSD-PBEP86-D3 aug-cc-pVTZ	Hyb revDSD PW6
ν_1	3178	3181	3182	3173	3170	3170	3169	3167	3148
ν_2	3096	3103	3099	3098	3092	3093	3092	3090	3084
ν_3	1709	1716	1712	1666	1661	1663	1662	1662	1663
ν_4	1331	1340	1332	1357	1352	1357	1352	1387	1388
ν_5	1167	1169	1170	1180	1167	1171	1182	1179	1182
ν_6	930	932	931	949	943	944	947	946	944
ν_7	701	701	702	689	689	692	699	698	698
ν_8	428	428	431	433	432	433	434	433	432
ν_9	368	368	370	373	371	373	371	371	369
ν_{10}	836	852	840	847	845	849	842	840	840
ν_{11}	701	702	700	715	711	713	715	714	710
ν_{12}	529	529	531	532	526	530	529	525	528
MD	2.0	6.1	4.6	4.2	-0.3	2.1	2.5	4.4	2.5
MAD	18.2	20.0	18.8	10.9	10.9	10.9	8.2	6.7	6.7
Max Pos	53.1	59.8	56.4	25.4	19.9	21.1	19.6	17.6	13.6
Max Neg	-43.8	-34.9	-43.0	-17.6	-23.0	-18.2	-23.1	-8.7	-10.6

[a] From Ref. (Pietropolli Charmet et al., 2016).
[b] a.e.-CCSD(T)/cc-p CVTZ/aug-cc-pCVTZ(F), from Ref. (Pietropolli Charmet et al., 2016).

Table S64. ClFC=CF_2 anharmonic fundamental frequencies (cm^{-1}) obtained at the different levels of theory and comparison to experimental data.

ClFC=CF_2	Exp[a]	Hyb2[b]	B3LYP-D3 SNSD	ωB97 jul-cc-pVDZ	ωB97 aug-cc-pVDZ	ωB97X jul-cc-pVDZ	ωB97X aug-cc-pVDZ	ωB97XD jul-cc-pVDZ	ωB97XD aug-cc-pVDZ
ν_1	1801	1801	1801	1865	1865	1863	1863	1845	1845
ν_2	1334	1339	1289	1312	1312	1317	1317	1314	1314
ν_3	1216	1220	1175	1210	1210	1199	1199	1193	1193
ν_4	1060	1051	1036	1070	1070	1067	1067	1057	1057
ν_5	691	687	681	695	695	696	696	692	692
ν_6	516	515	508	507	507	512	512	512	512
ν_7	462	459	452	463	463	463	463	460	460
ν_8	338	335	336	337	337	339	339	339	339
ν_9	188	186	188	187	187	189	189	188	188
ν_{10}	539	532	552	589	589	588	588	575	575
ν_{11}	368	367	369	382	382	381	381	379	379
ν_{12}	174	167	166	171	171	170	170	168	168
MD		-2.4	-11.2	8.4	8.4	8.1	8.1	3.0	2.9
MAD		3.8	13.8	15.5	15.5	15.2	15.2	12.7	12.7
Max Pos		4.6	13.7	64.6	64.6	62.0	62.0	44.6	44.6
Max Neg		-8.7	-45.8	-22.7	-22.8	-17.7	-17.7	-23.2	-23.2

ClFC=CF_2	PW6B95-D3 jul-cc-pVDZ	PW6B95-D3 aug-cc-pVDZ	PW6B95-D3 jun-cc-pVDZ	B2PLYP-D3 VTZ	B2PLYP-D3 augVTZ	B2PLYP-D3 may'VTZ	rev-DSD-PBEP86-D3 jun-cc-pVTZ	rev-DSD-PBEP86-D3 aug-cc-pVTZ	Hyb revDSD PW6
ν_1	1842	1842	1845	1805	1798	1799	1807	1807	1804
ν_2	1320	1319	1325	1320	1305	1310	1325	1322	1325
ν_3	1192	1192	1197	1197	1186	1189	1215	1197	1199
ν_4	1061	1061	1062	1052	1048	1052	1060	1058	1061
ν_5	693	693	697	691	687	690	692	691	692
ν_6	510	510	513	516	514	515	516	515	517
ν_7	460	460	463	459	458	460	462	461	463
ν_8	334	334	336	338	337	338	338	336	340
ν_9	184	184	184	188	187	189	187	186	192
ν_{10}	568	567	566	558	557	562	558	554	561
ν_{11}	375	375	377	377	373	376	376	372	376
ν_{12}	170	170	170	169	168	169	170	169	171
MD	1.8	1.7	3.9	-1.4	-5.9	-3.2	1.6	-1.6	1.1
MAD	11.6	11.6	10.9	7.0	9.7	8.6	4.4	5.9	6.1
Max Pos	41.0	41.0	44.0	19.6	18.1	23.6	19.7	15.0	21.8
Max Neg	-24.0	-24.0	-19.5	-19.4	-30.5	-27.2	-9.0	-19.7	-17.5

[a] From Ref. (Tasinato et al., 2012a).
[b] Geometry and harmonic force field at CCSD(T)/ANOtz level; cubic and quartic force constants at CCSD(T)/cc-pVTZ level, from Ref. (Tasinato et al., 2012a).

Table S65. Oxirane anharmonic fundamental frequencies (cm^{-1}) obtained at the different levels of theory and comparison to experimental data.

Oxirane	Exp[a]	CCSD(T)[b] CBS+CV+aug	B3LYP-D3 SNSD (PES1)	ωB97 jul-cc-pVDZ	ωB97 aug-cc-pVDZ	ωB97X jul-cc-pVDZ	ωB97X aug-cc-pVDZ	ωB97XD jul-cc-pVDZ	ωB97XD aug-cc-pVDZ
ν_1	3018	3022	2888	2879	2884	2897	2900	2846	2845
ν_2	1498	1490	1490	1503	1506	1503	1506	1499	1502
ν_3	1270	1266	1273	1306	1308	1303	1304	1290	1291
ν_4	n.a.	1115	1118	1119	1124	1120	1124	1106	1110
ν_5	877	874	869	919	921	907	908	884	885
ν_6	n.a.	3065	3020	2964	2967	2982	2982	2926	2922
ν_7	n.a.	1160	1151	1137	1139	1145	1147	1147	1148
ν_8	n.a.	1027	1018	1006	1007	1011	1012	1004	1004
ν_9	3066	3076	3035	2977	2980	2996	2996	2940	2937
ν_{10}	n.a.	1145	1144	1140	1140	1146	1145	1144	1143
ν_{11}	808	810	804	784	787	795	797	798	800
ν_{12}	3007	3009	2900	2847	2852	2866	2869	2821	2819
ν_{13}	1471	1461	1464	1448	1453	1455	1460	1459	1463
ν_{14}	n.a.	1154	1133	1111	1118	1119	1125	1110	1115
ν_{15}	822	816	820	889	889	876	877	853	853
MD		-1.6	-32.7	-31.7	-28.7	-26.7	-24.6	-49.7	-49.2
MAD		5.5	33.2	65.1	63.4	53.8	53.0	62.8	63.4
Max Pos		10.0	2.6	66.6	67.1	53.9	54.3	30.5	30.8
Max Neg		-10.6	-130.4	-159.5	-154.4	-141.0	-138.0	-186.0	-187.2

Oxirane	PW6B95-D3 jul-cc-pVDZ	PW6B95-D3 aug-cc-pVDZ	PW6B95-D3 jun-cc-pVDZ	B2PYLP VTZ (PES5)	B2PLYP-D3 augVTZ (PES4)	B2PLYP-D3 may'VTZ (PES2)	rev-DSD-PBEP86-D3 jun-cc-pVTZ	rev-DSD-PBEP86-D3 aug-cc-pVTZ	Hyb revDSD PW6
ν_1	3030	3036	3033	2919	2917	2920	2992	2988	3062
ν_2	1491	1495	1492	1504	1500	1503	1503	1500	1506
ν_3	1295	1296	1300	1275	1272	1274	1278	1276	1280
ν_4	1121	1126	1126	1126	1124	1127	1129	1127	1131
ν_5	896	896	900	872	866	867	880	879	879
ν_6	3069	3071	3071	3054	3057	3056	3060	3058	3061
ν_7	1144	1147	1144	1164	1163	1163	1162	1161	1156
ν_8	1019	1019	1017	1029	1028	1029	1030	1028	1034
ν_9	3083	3085	3085	3069	3071	3070	3074	3072	3075
ν_{10}	1144	1144	1145	1153	1149	1151	1152	1150	1169
ν_{11}	804	807	805	812	811	811	810	808	816
ν_{12}	3017	3023	3020	2931	3026	2932	3026	3023	3029
ν_{13}	1453	1459	1450	1480	1478	1480	1476	1474	1485
ν_{14}	1129	1134	1129	1140	1147	1148	1145	1144	1153
ν_{15}	852	853	859	815	808	811	824	822	821
MD	9.5	12.6	11.8	-17.8	-9.7	-18.8	2.8	0.5	12.9
MAD	15.9	16.2	18.6	23.8	18.3	24.3	8.7	7.4	13.1
Max Pos	30.0	30.9	37.2	8.9	19.5	8.9	20.0	16.2	43.8
Max Neg	-18.2	-12.8	-21.5	-99.6	-101.3	-98.2	-26.5	-30.2	-0.9

[a] From Ref. (Lafferty et al., 2013; Flaud et al., 2012; Russell and Wesendrup, 2003).
[b] CCSD(T)/CBS + Δ(CV) + Δ(aug) harmonic force field; cubic and semi-diagonal force constants evaluated at the CCSD(T)/cc-pVQZ level, from Ref. (Puzzarini et al., 2014a).

Table S66. CH_3CH_2SH anharmonic fundamental frequencies (cm^{-1}) obtained at the different levels of theory and comparison to experimental data.

CH_3CH_2SH	Exp[a]	CCSD(T)-F12[b] VTZ-F12	B3LYP-D3 SNSD	ωB97 jul-cc-pVDZ	ωB97 aug-cc-pVDZ	ωB97X jul-cc-pVDZ	ωB97X aug-cc-pVDZ	ωB97XD jul-cc-pVDZ	ωB97XD aug-cc-pVDZ
ν_1	2980	2987	3004	3032	3045	3038	3048	3009	2963
ν_2	2967	2972	2955	2968	2960	2960	2949	2941	2944
ν_3	2930	2962	2944	2936	2938	2949	2940	2926	2913
ν_4	2902	2955	2927	2929	2932	2936	2936	2905	2882
ν_5	2875	2988	2910	2895	2893	2906	2901	2892	2878
ν_6	2591	2596	2552	2426	2431	2452	2439	2298	2490
ν_7	1462	1469	1452	1431	1426	1440	1434	1438	1429
ν_8	1452	1467	1442	1431	1427	1436	1428	1434	1425
ν_9	1437	1394	1428	1421	1418	1432	1428	1436	1409
ν_{10}	1377	1386	1376	1376	1375	1377	1375	1377	1367
ν_{11}	1269	1322	1272	1283	1286	1280	1282	1282	1246
ν_{12}	1246	1266	1243	1257	1259	1255	1257	1258	1240
ν_{13}	1093	1102	1092	1113	1113	1111	1112	1109	1088
ν_{14}	1051	1149	1039	1066	1065	1062	1060	1054	1042
ν_{15}	970	966	954	993	993	991	990	980	969
ν_{16}	867	871	856	910	905	903	903	901	859
ν_{17}	735	743	727	745	749	745	747	755	722
ν_{18}	658	666	629	705	701	688	688	687	636
ν_{19}	319	326	326	340	340	339	339	340	349
ν_{20}	n.a.	253	236	208	210	216	217	226	279
ν_{21}	n.a.	211	202	194	199	193	195	141	198
MD		27.2	-1.8	-7.4	-7.3	-2.9	-5.3	-17.8	-18.7
MAD		33.9	12.6	28.7	29.8	25.2	27.7	33.9	23.9
Max Pos		113.0	34.7	27.1	29.7	34.4	34.4	21.2	30.0
Max Neg		-43.0	-39.2	-164.9	-159.7	-139.3	-151.7	-293.4	-100.9

[a] From Ref. (Smith et al., 1968; Wolff and Szydlowski, 1985; Miller et al., 2009).
[b] CCSD(T)-F12/cc-pVTZ-F12,level, from Ref. (Hochlaf et al., 2015).

	PW6B95-D3			B2PLYP-D3			rev-DSD-PBEP86-D3		Hyb revDSD
CH_3CH_2SH	jul-cc-pVDZ	aug-cc-pVDZ	jun-cc-pVDZ	VTZ	augVTZ	may'VTZ	jun-cc-pVTZ	aug-cc-pVTZ	PW6
ν_1	3064	3071	3066	3043	3040	3042	3042	3041	3046
ν_2	2991	2983	2996	2968	2971	2975	2970	2965	2978
ν_3	2977	2974	2985	2960	2971	2967	2966	2964	2974
ν_4	2976	2973	2974	2942	2956	2958	2958	2953	2960
ν_5	2927	2930	2929	2875	2880	2883	2871	2874	2864
ν_6	2619	2618	2621	2597	2596	2595	2615	2612	2633
ν_7	1447	1444	1439	1472	1470	1473	1469	1468	1473
ν_8	1438	1430	1431	1464	1462	1464	1462	1461	1466
ν_9	1423	1418	1420	1448	1447	1448	1445	1446	1449
ν_{10}	1367	1365	1365	1391	1389	1391	1387	1386	1388
ν_{11}	1260	1261	1265	1278	1277	1279	1281	1281	1282
ν_{12}	1241	1242	1242	1261	1260	1260	1259	1259	1260
ν_{13}	1096	1098	1099	1107	1106	1107	1108	1107	1112
ν_{14}	1047	1047	1048	1052	1051	1050	1053	1053	1077
ν_{15}	987	969	972	964	963	965	970	968	971
ν_{16}	861	864	864	868	868	870	870	869	872
ν_{17}	732	734	736	739	739	739	740	739	741
ν_{18}	654	656	656	647	646	648	659	659	660
ν_{19}	333	335	335	331	331	332	333	331	337
ν_{20}	236	243	251	242	242	243	243	238	247
ν_{21}	206	207	219	209	208	213	209	208	209
MD	7.6	6.6	7.4	11.4	11.9	13.2	13.1	12.5	17.9
MAD	18.9	20.2	20.3	11.4	11.9	13.3	12.0	10.9	17.0
Max Pos	74.1	70.5	72.2	40.2	54.1	55.9	55.5	51.3	58.3
Max Neg	-14.9	-22.4	-22.8	-0.2	0.3	-0.9	-4.5	-0.7	-11.3

Table S67. SO_2 anharmonic fundamental frequencies (cm^{-1}) obtained at the different levels of theory and comparison to experimental data.

SO_2	Exp[a]	Cheap[b]	B3LYP-D3 SNSD	ωB97 jul-cc-pVDZ	ωB97 aug-cc-pVDZ	ωB97X jul-cc-pVDZ	ωB97X aug-cc-pVDZ	ωB97XD jul-cc-pVDZ	ωB97XD aug-cc-pVDZ
ν_1	1152	1152	1090	1205	1205	1201	1201	1193	1193
ν_2	518	518	486	515	515	516	516	513	513
ν_3	1362	1355	1241	1395	1395	1394	1394	1389	1389
MD		-2.4	-71.5	27.8	27.8	26.2	26.2	21.1	21.1
MAD		2.6	71.5	29.8	29.8	27.7	27.7	24.3	24.3
Max Pos		0.3	-31.9	53.0	53.0	49.2	49.2	41.6	41.6
Max Neg		-7.3	-120.6	-2.9	-2.9	-2.4	-2.4	-4.8	-4.8

SO_2	PW6B95-D3 jul-cc-pVDZ	PW6B95-D3 aug-cc-pVDZ	PW6B95-D3 jun-cc-pVDZ	B2PLYP-D3 VTZ	B2PLYP-D3 augVTZ	B2PLYP-D3 may'VTZ	rev-DSD-PBEP86-D3 jun-cc-pVTZ	rev-DSD-PBEP86-D3 aug-cc-pVTZ	Hyb revDSD PW6
ν_1	1159	1115	1167	1106	1096	1123	1135	1133	1139
ν_2	507	513	514	503	499	507	510	510	511
ν_3	1352	1295	1364	1302	1285	1330	1344	1342	1348
MD	-4.8	-36.3	4.4	-40.2	-50.6	-24.0	-13.9	-15.7	-11.1
MAD	9.4	36.3	7.1	40.2	50.6	24.0	13.9	15.7	11.1
Max Pos	6.8	-4.9	15.5	-14.7	-19.2	-10.8	-7.5	-8.3	-6.7
Max Neg	-10.8	-67.2	-4.0	-60.1	-77.1	-32.5	-17.7	-20.5	-14.1

[a] From Ref. (Flaud et al., 2012).
[b] Cheap composite scheme, from Ref. (Boussessi et al., 2020).

Table S68. $HCOCH_2OH$ anharmonic fundamental frequencies (cm^{-1}) obtained at the different levels of theory and comparison to experimental data.

	Exp[a]	cheap[b]	B3LYP-D3	ωB97		ωB97X		ωB97XD	
$HCOCH_2OH$			SNSD	jul-cc-pVDZ[c]	aug-cc-pVDZ[c]	jul-cc-pVDZ	aug-cc-pVDZ[c]	jul-cc-pVDZ	aug-cc-pVDZ
ν_1	3549	3549	3527	3659	3664	3649	3660	3580	3588
ν_2	2885	2878	2810	2918	2851	2915	2929	2885	2879
ν_3	2832	2829	2796	2980	2975	2953	2951	2972	2967
ν_4	1754	1752	1769	1819	1818	1814	1814	1804	1803
ν_5	1458	1449	1419	1447	1459	1419	1457	1412	1385
ν_6		1399	1388	1474	1498	1490	1515	904	1012
ν_7	1364	1358	1352	1353	1357	1341	1360	1368	1370
ν_8	1275	1266	1257	1398	1450	1382	1466	719	832
ν_9	1115	1111	1107	1164	1177	1154	1177	1006	1027
ν_{10}	861	860	848	882	882	879	880	863	866
ν_{11}	752	744	746	769	780	762	780	641	662
ν_{12}		274	272	462	556	404	573	-711	-503
ν_{13}	2881	2893	2838	2904	2904	2909	2906	2889	2882
ν_{14}	1229	1234	1217	1211	1211	1216	1216	1215	1212
ν_{15}	1084	1083	1074	1088	1090	1084	1087	1083	1086
ν_{16}	700	714	702	701	702	703	703	699	698
ν_{17}	360	362	352	370	366	432	424	514	520
ν_{18}	207	218	216	204	203	223	221	236	238
MD		-0.4	-17.3	34.9	36.4	33.0	45.3	-26.3	-18.2
MAD		5.8	20.5	40.3	44.4	42.4	47.5	78.5	71.5
Max Pos		13.8	15.0	148.0	174.4	120.7	191.0	154.5	160.9
Max Neg		-9.0	-74.8	-18.0	-34.2	-38.8	-13.3	-556.7	-443.8

[a] From Ref. (Johnson et al., 2013).
[b] Cheap harmonic frequencies, cubic and semidiagonal quartic force constants at B2PLYP-D3/maug-cc-pVTZ-dH level, from Ref. (Boussessi et al., 2020).
[c] Normal mode 11 was removed from the anharmonic calculation.

$HCOCH_2OH$	PW6B95-D3 jul-cc-pVDZ	PW6B95-D3 aug-cc-pVDZ	PW6B95-D3 jun-cc-pVDZ	B2PLYP-D3 VTZ	B2PLYP-D3 augVTZ	B2PLYP-D3 may'VTZ	rev-DSD-PBEP86-D3 jun-cc-pVTZ	rev-DSD-PBEP86-D3 aug-cc-pVTZ	Hyb revDSD PW6
ν_1	3581	3577	3559	3544	3535	3547	3556	3553	3575
ν_2	2915	2911	2918	2866	2846	2850	2868	2867	2886
ν_3	2810	2833	2808	2827	2822	2823	2832	2830	2843
ν_4	1797	1797	1806	1755	1745	1748	1756	1755	1756
ν_5	1420	1421	1433	1464	1457	1460	1439	1440	1458
ν_6	1384	1380	1385	1405	1394	1398	1404	1402	1408
ν_7	1349	1351	1348	1371	1366	1368	1368	1368	1370
ν_8	1263	1258	1286	1276	1265	1265	1270	1268	1296
ν_9	1129	1128	1140	1111	1104	1106	1114	1112	1125
ν_{10}	870	870	870	855	852	852	858	859	857
ν_{11}	749	749	754	751	746	745	744	746	751
ν_{12}	270	263	326	280	273	273	275	279	322
ν_{13}	2897	2897	2898	2876	2879	2879	2888	2887	2892
ν_{14}	1221	1220	1220	1231	1230	1231	1232	1233	1239
ν_{15}	1073	1076	1073	1092	1086	1088	1088	1088	1089
ν_{16}	703	703	703	719	712	714	716	715	717
ν_{17}	416	416	407	358	349	347	355	355	412
ν_{18}	232	231	231	215	210	209	217	215	229
MD	7.6	8.3	9.3	0.3	-6.3	-4.5	-0.3	-0.9	11.9
MAD	21.2	19.3	20.0	6.3	8.7	8.1	6.9	6.5	12.4
Max Pos	56.8	56.4	51.8	18.9	11.5	14.0	15.8	14.2	52.7
Max Neg	-37.8	-37.5	-25.3	-19.3	-38.8	-34.9	-18.9	-18.4	-3.6

Table S69. *E*-Ethanimine anharmonic fundamental frequencies (cm^{-1}) obtained at the different levels of theory and comparison to experimental data.

	Exp[a]	Hyb[b]	B3LYP-D3	ωB97		ωB97X		ωB97XD	
E-Ethanimine			SNSD	jul-cc-pVDZ	aug-cc-pVDZ	jul-cc-pVDZ	aug-cc-pVDZ	jul-cc-pVDZ	aug-cc-pVDZ
ν_1	3247	3300	3288	3421	3427	3370	3377	3332	3340
ν_2	3018	3015	2989	3028	3023	3025	3020	3008	3000
ν_3	2925	2941	2917	2855	2854	2871	2870	2839	2833
ν_4	2885	2881	2836	2905	2905	2908	2913	2894	2892
ν_5	1652	1665	1682	1745	1744	1729	1728	1710	1710
ν_6	1438	1442	1427	1400	1386	1423	1398	1418	1404
ν_7	1392	1395	1403	1389	1397	1391	1392	1384	1383
ν_8	1358	1362	1351	1336	1334	1343	1341	1340	1338
ν_9	1252	1250	1241	1241	1240	1244	1243	1240	1240
ν_{10}	1045	1044	1033	1044	1042	1043	1041	1037	1036
ν_{11}	950	924	914	931	930	931	929	927	925
ν_{12}	485	488	489	492	491	492	492	491	492
ν_{13}		2967	2930	2894	2893	2911	2908	2857	2851
ν_{14}	1435	1444	1428	1427	1420	1429	1422	1433	1427
ν_{15}	1106	1095	1094	1125	1129	1118	1122	1118	1123
ν_{16}	1052	1050	1038	1045	1043	1048	1046	1050	1048
ν_{17}	668	679	679	698	697	694	694	702	703
ν_{18}		171	178	135	132	148	146	151	148
MD		4.2	-6.2	10.9	9.8	9.4	7.5	1.0	-1.0
MAD		10.3	18.3	33.2	35.5	25.1	28.1	24.5	28.0
Max Pos		53.0	41.4	174.3	180.4	123.2	130.0	84.7	92.9
Max Neg		-26.0	-49.1	-70.1	-71.0	-54.4	-55.1	-86.2	-91.7

[a] From Ref. (Melli et al., 2018).
[b] Geometry and harmonic force field at a.e-CCSD(T)/cc-pCVQZ level; cubic and quartic force constants at B2PLYP-D3/maug-cc-pVTZ-dH level, from Ref. (Melli et al., 2018).

E-Ethanimine	PW6B95-D3 jul-cc-pVDZ	PW6B95-D3 aug-cc-pVDZ	PW6B95-D3 jun-cc-pVDZ	B2PLYP-D3 VTZ	B2PLYP-D3 augVTZ	B2PLYP-D3 may'VTZ	rev-DSD-PBEP86-D3 jun-cc-pVTZ	rev-DSD-PBEP86-D3 aug-cc-pVTZ	Hyb revDSD PW6
ν_1	3296	3299	3284	3298	3300	3298	3300	3298	3267
ν_2	3032	3032	3036	3018	3016	3017	3018	3017	3009
ν_3	2948	2946	2952	2948	2945	2946	2946	2946	2953
ν_4	2872	2876	2869	2862	2860	2860	2874	2872	2865
ν_5	1708	1708	1714	1672	1663	1665	1666	1665	1666
ν_6	1426	1420	1424	1445	1444	1446	1444	1445	1451
ν_7	1407	1405	1390	1413	1390	1392	1391	1390	1409
ν_8	1344	1341	1343	1366	1364	1366	1362	1362	1366
ν_9	1243	1242	1239	1253	1249	1249	1250	1249	1250
ν_{10}	1036	1034	1035	1043	1041	1042	1043	1042	1042
ν_{11}	931	930	930	921	921	921	923	923	925
ν_{12}	481	480	485	490	490	491	487	488	487
ν_{13}	2990	2988	2997	2966	2963	2964	2969	2968	2974
ν_{14}	1422	1415	1412	1449	1447	1449	1447	1448	1450
ν_{15}	1092	1095	1092	1111	1105	1106	1103	1094	1094
ν_{16}	1043	1042	1039	1055	1050	1051	1049	1050	1055
ν_{17}	667	667	665	687	684	684	681	683	665
ν_{18}	204	202	204	180	178	175	177	180	197
MD	2.6	1.4	0.1	7.6	3.8	4.6	4.9	4.0	3.0
MAD	17.1	18.1	17.8	14.3	12.3	12.4	11.0	11.9	12.1
Max Pos	56.4	56.5	61.7	50.7	52.6	50.7	53.4	50.9	27.9
Max Neg	-18.8	-20.2	-23.1	-28.7	-29.1	-29.2	-26.8	-27.1	-24.7

1.9 Anharmonic Infrared Intensities

Table S70. CH_2F_2 anharmonic IR intensities (km mol^{-1}) obtained at the different levels of theory and comparison to experimental values.

Integration limits	Exp[a]	B3LYP-D3 SNSD	PW6B95-D3 jun-cc-pVDZ	PW6B95-D3 jul-cc-pVDZ	PW6B95-D3 aug-cc-pVDZ
425-625	4.97	4.44	5.31	4.79	4.81
924-1250	337	376.82	425.10	377.12	377.42
1371-1475	10.03	8.22	19.61	9.87	8.16
1478-1550	0.38	0.28	0	0.30	0
2737-3151	67.30	77.12	77.80	73.08	74.01
MD		9.44	21.63	9.10	8.94
MAD		10.41	21.78	9.26	9.91
Max Pos		39.82	88.10	40.12	40.42
Max Neg		-1.81	-0.38	-0.18	-1.87

Integration limits	B2PLYP-D3 VTZ	B2PLYP-D3 augVTZ	B2PLYP-D3 may'VTZ	rev-DSD-PBEP86-D3 jun-cc-pVTZ	rev-DSD-PBEP86-D3 aug-cc-pVTZ	Hyb revDSD/PW6
425-625	4.39	4.52	4.68	4.73	4.70	4.73
924-1250	356.30	372.50	389.07	366.07	365.42	366.99
1371-1475	14.72	9.15	11.21	10.55	10.46	9.74
1478-1550	0.78	0.17	0.37	0.22	0.23	0.23
2737-3151	93.78	74.33	79.72	76.01	76.27	76.06
MD	10.06	8.20	13.07	7.58	7.48	7.61
MAD	10.29	8.81	13.20	7.74	7.65	7.88
Max Pos	26.48	35.50	52.07	29.07	28.42	29.99
Max Neg	-0.58	-0.88	-0.29	-0.24	-0.27	-0.29

[a] From Ref. (Tasinato et al., 2012b).

Table S71. CH_2ClF anharmonic IR intensities (km mol^{-1}) obtained at the different levels of theory and comparison to experimental values.

Integration limits	Exp[a]	B3LYP-D3 SNSD	PW6B95-D3 jun-cc-pVDZ	PW6B95-D3 jul-cc-pVDZ	PW6B95-D3 aug-cc-pVDZ
700-800	78.60	104.35	107.82	99.01	99.18
950-1175	144	173.25	196.46	169.55	169.91
1175-1290	2.40	2.38	2.98	2.34	2.28
1290-1410	25.41	25.96	36.36	24.71	24.28
1430-1550	1.51	1.46	1.51	1.25	1.49
2850-3115	26.30	29.70	20.69	28.05	29.50
MD		9.81	14.60	7.78	8.07
MAD		9.84	16.47	8.12	8.49
Max Pos		29.25	52.46	25.55	25.91
Max Neg		-0.05	-5.61	-0.70	-1.13

Integration limits	B2PLYP-D3 VTZ	B2PLYP-D3 augVTZ	B2PLYP-D3 may'VTZ	rev-DSD-PBEP86-D3 jun-cc-pVTZ	rev-DSD-PBEP86-D3 aug-cc-pVTZ	Hyb revDSD/ PW6
700-800	98.88	100.86	102.78	93.23	93.02	93.59
950-1175	163.80	166.33	174.50	161.97	161.73	162.25
1175-1290	2.62	2.36	2.59	2.45	2.39	2.44
1290-1410	31.36	26.74	30.25	27.72	27.56	26.85
1430-1550	1.14	1.33	1.37	1.25	1.22	1.34
2850-3115	37.53	28.08	31.32	29.60	29.64	30.15
MD	9.52	7.91	10.77	6.33	6.22	6.40
MAD	9.64	7.99	10.81	6.42	6.33	6.45
Max Pos	20.28	22.33	30.50	17.97	17.73	18.25
Max Neg	-0.37	-0.18	-0.14	-0.26	-0.29	-0.17

[a] From Ref. (Pietropolli Charmet et al., 2013).

Table S72. *cis*-$ClFC=CH_2$ anharmonic IR intensities (km mol^{-1}) obtained at the different levels of theory and comparison to experimental values.

Integration limits	Exp[a]	B3LYP-D3 SNSD	PW6B95-D3 jun-cc-pVDZ	PW6B95-D3 jul-cc-pVDZ	PW6B95-D3 aug-cc-pVDZ
400-455	1.63	0.81	1.46	1.48	1.48
500-520	0.94	1.93	0	0	0
650-750	39.30	49.49	47.93	43.88	44.08
780-900	50.60	55.44	57.04	56.95	56.30
900-1000	41.90	50.98	46.82	43.28	44.43
1090-1260	166.20	181.08	218.06	187.30	184.67
1260-1440	5.27	4.51	4.53	3.82	3.86
1580-1790	130	164.88	173.28	162.37	163.46
2920-3190	1.87	2.95	5.04	3.91	3.45
MD		8.26	12.94	7.25	7.11
MAD		8.61	13.35	7.82	7.67
Max Pos		34.88	51.86	32.37	33.46
Max Neg		-0.82	-0.94	-1.45	-1.41

Integration limits	B2PLYP-D3 VTZ	B2PLYP-D3 augVTZ	B2PLYP-D3 may'VTZ	rev-DSD-PBEP86-D3 jun-cc-pVTZ	rev-DSD-PBEP86-D3 aug-cc-pVTZ	Hyb revDSD/ PW6
400-455	0.95	1.01	1.08	1.30	1.33	1.41
500-520	0	0	0	0	0	0
650-750	46.85	45.26	45.51	41.32	41.39	41.22
780-900	58.73	54.33	58.57	53.83	53.99	58.39
900-1000	42.89	48.42	49.39	42.36	42.86	43.21
1090-1260	176.53	174.11	183.93	172.69	171.68	171.48
1260-1440	5.15	4.49	4.84	4.32	4.58	4.31
1580-1790	159.35	157.34	161.34	148.78	148.20	148.87
2920-3190	2.47	2.81	2.80	2.37	2.38	2.39
MD	6.13	5.56	7.75	3.25	3.19	3.73
MAD	6.52	6.08	8.18	3.74	3.62	4.20
Max Pos	29.35	27.34	31.34	18.78	18.20	18.87
Max Neg	-0.94	-0.94	-0.94	-0.95	-0.94	-0.96

[a] From Ref. (Craig et al., 1970).

Table S73. $ClFC{=}CF_2$ anharmonic IR intensities (km mol^{-1}) obtained at the different levels of theory and comparison to experimental values.

Integration limits	Exp[a]	B3LYP-D3 SNSD	PW6B95-D3 jun-cc-pVDZ	PW6B95-D3 jul-cc-pVDZ	PW6B95-D3 aug-cc-pVDZ
430-490	0.95	0.43	0.77	0.87	0.87
490-575	3.16	2.89	3.76	3.10	3.10
660-775	3.30	4.23	3.45	3.10	3.10
950-1120	210.20	240.04	256.82	235.11	235.09
1168-1275	189	206.68	231.34	199.33	199.14
1280-1365	153.80	181.01	204.26	178.63	179.32
1690-1859	55.24	55.15	60.33	57.95	57.95
MD		10.68	20.73	8.92	8.99
MAD		10.93	20.78	9.02	9.09
Max Pos		29.84	50.46	24.91	25.52
Max Neg		-0.52	-0.18	-0.20	-0.20

Integration limits	B2PLYP-D3 VTZ	B2PLYP-D3 augVTZ	B2PLYP-D3 may'VTZ	rev-DSD-PBEP86-D3 jun-cc-pVTZ	rev-DSD-PBEP86-D3 aug-cc-pVTZ	Hyb revDSD/ PW6
430-490	0.41	0.58	0.61	0.76	0.79	0.76
490-575	2.95	2.85	2.99	2.87	2.98	2.83
660-775	4.13	3.72	3.59	3.27	3.26	3.21
950-1120	231.68	231.39	229.64	226.48	225.39	216.88
1168-1275	201.70	194.05	206.57	191.55	190.13	192.77
1280-1365	165.11	177.16	182.86	170.98	172.40	167.72
1690-1859	57.81	59.46	61.55	59.96	59.87	59.53
MD	6.88	7.65	10.31	5.75	5.59	4.01
MAD	7.09	7.85	10.45	5.89	5.70	4.18
Max Pos	21.48	23.36	29.06	17.18	18.60	13.92
Max Neg	-0.54	-0.37	-0.34	-0.29	-0.18	-0.33

[a] From Ref. (Pietropolli Charmet et al., 2016).

Table S74. SO_2 anharmonic IR intensities (km mol^{-1}) obtained at the different levels of theory and comparison to experimental values.

Band	Exp[a]	B3LYP-D3 SNSD	PW6B95-D3 jun-cc-pVDZ	PW6B95-D3 jul-cc-pVDZ	PW6B95-D3 aug-cc-pVDZ
ν_1	21.20	24.24	36.41	29.82	28.43
ν_2	25.54	26.62	38.59	29.60	28.71
ν_3	167.47	200.69	244.23	218.69	212.56
MD		12.45	35.01	21.30	18.50
MAD		12.45	35.01	21.30	18.50
Max Pos		33.22	76.76	51.22	45.09
Max Neg		1.08	13.05	4.06	3.17

Integration limits	B2PLYP-D3 VTZ	B2PLYP-D3 augVTZ	B2PLYP-D3 may'VTZ	rev-DSD-PBEP86-D3 jun-cc-pVTZ	rev-DSD-PBEP86-D3 aug-cc-pVTZ	Hyb revDSD/ PW6
1	19.49	19.92	24.43	22.42	22.35	22.58
2	27.06	25.64	28.25	26.87	26.74	26.90
3	149.81	170.27	182.43	175.30	175.01	176.74
MD	-5.95	0.54	6.97	3.46	3.30	4.01
MAD	6.96	1.39	6.97	3.46	3.30	4.01
Max Pos	1.52	2.80	14.97	7.83	7.54	9.27
Max Neg	-17.65	-1.28	2.71	1.22	1.15	1.36

[a] From Ref. (Tasinato et al., 2010).

1.10 Global Statistics

Table S75. Equilibrium bond length global statistics in Å obtained at the different levels of theory.

Global	CCSD(T)	B3 SNSD	ωB97 julDZ	ωB97 augDZ	ωB97X julDZ	ωB97X augDZ	ωB97XD julDZ	ωB97XD augDZ
MD	-0.0005	0.0099	0.0082	0.0079	0.0067	0.0063	0.0069	0.0068
MAD	0.0012	0.0100	0.0095	0.0091	0.0074	0.0071	0.0074	0.0071
Max Pos	0.0026	0.0514	0.0214	0.0212	0.0143	0.0140	0.0131	0.0131
Max Neg	-0.0030	-0.0015	-0.0101	-0.0101	-0.0063	-0.0063	-0.0041	-0.0036

Global	PW6 julDZ	PW6 augDZ	PW6 junDZ	B2 VTZ	B2 augVTZ	B2 may'VTZ	rDSD junTZ	rDSD augTZ
MD	0.0036	0.0032	0.0034	0.0027	0.0032	0.0022	0.0031	0.0033
MAD	0.0050	0.0047	0.0060	0.0035	0.0039	0.0030	0.0035	0.0037
Max Pos	0.0146	0.0146	0.0113	0.0254	0.0273	0.0133	0.0113	0.0118
Max Neg	-0.0089	-0.0089	-0.0197	-0.0060	-0.0060	-0.0054	-0.0035	-0.0037

Table S76. Global bond angles statistics in degree obtained at the different levels of theory and comparison to the semi-experimental equilibrium geometry.

Global	CCSD(T)	B3 SNSD	ωB97 julDZ	ωB97 augDZ	ωB97X julDZ	ωB97X augDZ	ωB97XD julDZ	ωB97XD augDZ
MD	-0.03	0.18	0.10	0.09	0.13	0.11	0.13	0.16
MAD	0.11	0.42	0.29	0.28	0.30	0.28	0.31	0.34
Max Pos	0.23	2.56	1.50	1.10	1.96	1.55	1.50	2.91
Max Neg	-0.42	-1.99	-0.65	-0.65	-0.71	-0.70	-0.76	-0.74

Global	PW6 julDZ	PW6 augDZ	PW6 junDZ	B2 VTZ	B2 augVTZ	B2 may'VTZ	rDSD junTZ	rDSD augTZ
MD	0.08	0.08	0.11	0.05	0.10	0.10	0.03	0.03
MAD	0.21	0.27	0.30	0.30	0.28	0.27	0.16	0.15
Max Pos	0.68	1.19	1.02	1.52	1.48	0.82	0.68	0.68
Max Neg	-0.73	-0.96	-0.88	-0.84	-0.96	-0.80	-0.58	-0.57

Table S77. Global rotational constant statistics in cm^{-1} obtained at the different levels of theory and comparison to the experimental results.

Global	CCSD(T)	B3 SNSD	ωB97 julDZ	ωB97 augDZ	ωB97X julDZ	ωB97X augDZ	ωB97XD julDZ	ωB97XD augDZ
MD	-0.61675	-2.30166	-1.06868	-1.05336	-0.84824	-0.70369	-0.96248	-1.03876
MAD	0.82126	2.30166	1.06868	1.05336	0.91946	0.77624	0.99006	1.13944
Max Pos	0.99761	-0.04675	-0.08659	-0.04236	0.57800	0.55825	0.28127	0.70065
Max Neg	-11.95	-12.13	-3.13	-3.13	-2.80	-2.80	-2.54	-2.54

Global	PW6 julDZ	PW6 augDZ	PW6 junDZ	B2 VTZ	B2 augVTZ	B2 may'VTZ	rDSD junTZ	rDSD augTZ
MD	-0.58	-0.76	-0.46	-1.00	-1.15	-0.80	-0.75	-0.77
MAD	0.64	0.84	0.59	1.06	1.20	0.89	0.75	0.77
Max Pos	0.43	0.49	0.83	0.40	0.57	0.56	0.10	-0.01
Max Neg	-2.67	-6.48	-1.91	-5.75	-6.46	-1.82	-1.68	-1.85

Table S78. Global Quartic Centrifugal Distortion Constant statistics (in cm^{-1}) obtained at the different levels of theory and comparison to the experimental results.

Global	CCSD(T)	B3 SNSD	ωB97 julDZ	ωB97 augDZ	ωB97X julDZ	ωB97X augDZ	ωB97XD julDZ	ωB97XD augDZ
MD	-1.80	-3.23	-5.89	-5.94	-5.42	-5.38	-3.98	-5.39
MAD	3.77	5.53	6.70	6.53	6.00	5.72	6.39	6.10
Max Pos	16.12	19.44	14.81	9.16	9.91	4.34	29.11	5.31
Max Neg	-20.04	-25.87	-22.03	-18.09	-19.88	-16.02	-19.30	-16.20

Global	PW6 julDZ	PW6 augDZ	PW6 junDZ	B2 VTZ	B2 augVTZ	B2 may'VTZ	rDSD junTZ	rDSD augTZ
MD	-1.45	-1.97	-2.56	-2.19	-1.69	-1.62	-2.27	-2.16
MAD	5.58	6.24	6.12	4.50	3.53	3.35	3.93	3.95
Max Pos	38.25	32.7036	25.24	21.5925	17.78	14.00	20.28	20.79
Max Neg	-26.80	-23.14	-23.68	-26.83	-23.08	-21.59	-23.53	-23.43

Table S79. Global Harmonic Frequencies statistics in cm^{-1} obtained at the different levels of theory.

Global	B3 SNSD	ωB97 julDZ	ωB97 augDZ	ωB97X julDZ	ωB97X augDZ	ωB97XD julDZ	ωB97XD augDZ
MD	-14.0	9.5	9.8	8.2	8.9	0.9	1.3
MAD	16.6	23.4	24.0	18.8	19.8	13.9	14.4
Max Pos	22.6	80.1	79.1	73.9	72.8	55.0	55.8
Min Neg	-117.0	-51.8	-58.1	-42.7	-49.8	-38.7	-46.5

Global	PW6 julDZ	PW6 augDZ	PW6 junDZ	B2 VTZ	B2 augVTZ	B2 may'VTZ	rDSD junTZ	rDSD augTZ
MD	0.8	0.6	0.9	1.7	-2.2	0.2	2.3	0.7
MAD	14.9	17.0	15.3	8.2	8.1	7.5	5.7	5.2
Max Pos	50.7	49.5	54.6	34.3	30.1	35.9	32.3	24.8
Min Neg	-39.1	-67.76	-50.6	-53.1	-70.1	-32.2	-16.2	-18.2

Table S80. Global harmonic IR intensity statistics (km mol^{-1}) obtained at the different levels of theory.

Global	B3 SNSD	ωB97 julDZ	ωB97 augDZ	ωB97X julDZ	ωB97X augDZ	ωB97XD julDZ	ωB97XD augDZ
MD	4.42	4.33	4.30	4.59	4.57	4.87	4.88
MAD	5.20	5.15	5.14	5.16	5.12	5.52	5.51
Max Pos	39.40	72.06	72.06	66.08	66.08	56.88	56.88
Max Neg	-15.00	-11.12	-7.86	-13.23	-11.46	-14.26	-12.71

Global	PW6 julDZ	PW6 augDZ	PW6 junDZ	B2 VTZ	B2 augVTZ	B2 may'VTZ	rDSD junTZ	rDSD augTZ
MD	4.21	4.11	8.08	2.67	2.57	4.10	1.77	1.75
MAD	4.92	4.91	9.20	4.20	3.32	4.68	2.23	2.28
Max Pos	46.43	40.40	73.40	31.65	27.79	31.46	19.17	19.00
Max Neg	-16.85	-15.71	-17.87	-20.75	-15.33	-16.36	-6.40	-7.82

Table S81. Global anharmonic fundamental frequency statistics (cm^{-1}) obtained at the different levels of theory and comparison to experimental data.

Global	CCSD(T)	B3 SNSD	ωB97 julDZ	ωB97 augDZ	ωB97X julDZ	ωB97X augDZ	ωB97XD julDZ	ωB97XD augDZ
MD	3	-15	4	4	5	6	-14	-14
MAD	8	20	33	34	29	30	36	35
Max Pos	113	41	174	180	123	191	155	161
Max Neg	-43	-130	-165	-160	-141	-152	-557	-444

Global	PW6 julDZ	PW6 augDZ	PW6 junDZ	B2 VTZ	B2 augVTZ	B2 may'VTZ	rDSD junTZ	rDSD augTZ	Hyb rDSD PW6
MD	3	3	4	1	-2	-1	3	2	7
MAD	18	20	18	12	12	12	8	8	11
Max Pos	74	71	72	51	54	56	56	51	58
Max Neg	-44	-67	-43	-100	-101	-98	-27	-30	-25

Table S82. Global anharmonic IR intensities statistic (km mol^{-1}) obtained at the different levels of theory and comparison to experimental values.

Global	B3 SNSD	PW6 junDZ	PW6 julDZ	PW6 augDZ	B2 VTZ	B2 augVTZ	B2 may'VTZ	rDSD junTZ	rDSD augTZ	Hyb rDSD/ PW6
MD	9.75	18.74	9.46	9.19	6.43	6.46	9.76	5.19	5.08	5.00
MAD	10.08	19.28	9.75	9.62	7.95	6.86	9.95	5.42	5.29	5.24
Max Pos	39.82	88.10	51.22	45.09	29.35	35.50	52.07	29.07	28.42	29.99
Max Neg	-1,81	-5.61	-1.45	-1.87	-17.65	-1.28	-0.94	-0.95	-0.94	-0.96

REFERENCES

Alonso, J. L., Lesarri, A., Leal, L. A., and Lòpez, J. C. (1993). The millimeter-wave spectra of 1-chloro-1-fluoroethylene and cis-1-chloro-2-fluoroethylene. *Journal of Molecular Spectroscopy* 162, 4–9. doi:10.1006/jmsp.1993.1265

Blanco, S., Lesarri, A., Alonso, J. L., and Guarnieri, A. (1995). The rotational spectrum of chlorofluoromethane. *Journal of Molecular Spectroscopy* 174, 397–416. doi:10.1006/jmsp.1995.0011

Boussessi, R., Ceselin, G., Tasinato, N., and Barone, V. (2020). DFT meets the segmented polarization consistent basis sets: Performances in the computation of molecular structures, rotational and vibrational spectroscopic properties. *Journal of Molecular Structure* 1208, 127886. doi:10.1016/j.molstruc.2020.127886

Carlotti, M., Nivellini, G., Tullini, F., and Carli, B. (1988). The far-infrared spectrum of methylene fluoride. *Journal of Molecular Spectroscopy* 132, 158–165. doi:10.1016/0022-2852(88)90065-3

Carroll, P. B., Drouin, B. J., and Widicus-Weaver, S. L. (2010). The submillimeter spectrum of glycolaldehyde. *The Astrophysical Journal* 723, 845–849. doi:10.1088/0004-637X/723/1/845

Craig, N. C., Lo, Y. S., Piper, L. G., and Wheeler, J. C. (1970). Vibrational assignments and potential constants for cis- and trans-1-chloro-2-fluoroethylenes and their deuterated modifications. *Journal of Physical Chemistry* 74, 1712–1727. doi:10.1021/j100703a011

Flaud, J.-M., Lafferty, W. J., Kwabia-Tchana, F., Perrin, A. M., and Landsheere, X. (2012). First high-resolution analysis of the ν_{15}, ν_{12}, ν_5, ν_{10} and ν_2 bands of oxirane. *Journal of Molecular Spectroscopy* 271, 38–43. doi:10.1016/j.jms.2011.11.005

Gambi, A., Pietropolli Charmet, A., Stoppa, P., Tasinato, N., Ceselin, G., and Barone, V. (2019). Molecular synthons for accurate structural determinations: The equilibrium geometry of 1-chloro-1-fluoroethene. *Physical Chemistry Chemical Physics* 21, 3615–3625. doi:10.1039/C8CP04888F

Gambi, A., Puzzarini, C., Cazzoli, G., Dore, L., and Palmieri, P. (2002). The anharmonic force field of cis-1-chloro-2-fluoroethylene. *Molecular Physics* 100, 3535–3543. doi:10.1080/00268970210130155

Hillig, K. W., Bittner, E. R., Kuczkowski, R. L., Lewis-Bevan, W., and Gerry, M. C. L. (1988). The chlorine nuclear quadrupole coupling tensor in chlorotrifluoroethylene. *Journal of Molecular Spectroscopy* 132, 369–379. doi:10.1016/0022-2852(88)90332-3

Hochlaf, M., Puzzarini, C., and Senent, M. L. (2015). Towards the computations of accurate spectroscopic parameters and vibrational spectra for organic compounds. *Molecular Physics* 113, 1661–1673. doi:10.1080/00268976.2014.1003986

Johnson, T. J., Sams, R. L., Profeta, L. T. M., Akagi, S. K., Burling, I. R., Yokelson, R. J., et al. (2013). Quantitative IR spectrum and vibrational assignments for glycolaldehyde vapor: Glycolaldehyde measurements in biomass burning plumes. *Journal of Phyisical Chemistry A* 117, 4096–4107. doi:10.1021/jp311945p

Kolesniková, L., Tercero, B., Cernicharo, J., Alonso, J. L., Daly, A. M., Gordon, B. P., et al. (2014). Spectroscopic characterization and detection of ethyl mercaptan in orion. *Astrophysical Journal Letters* 784, L7. doi:10.1088/2041-8205/784/1/L7

Lafferty, W. J., Flaud, J. M., Tchana, F. K., and Fernandez, J. M. (2013). Raman and infrared spectra of the ν_1 band of oxirane. *Molecular Physics* 111, 1983–1986. doi:10.1080/00268976.2013.775516

Leung, H. O., Marshall, M. D., Vasta, A. L., and Craig, N. C. (2009). Microwave spectra of eight isotopic modifications of 1-chloro-1-fluoroethylene. *Journal of Molecular Spectroscopy* 253, 116–121. doi:10.1016/j.jms.2008.11.002

Medcraft, C., Thompson, C. D., Robertson, E. G., Appadoo, D. R. T., and McNaughton, D. (2012). The far-infrared rotational spectrum of ethylene oxide. *The Astrophysical Journal* 753, 18. doi:10.1088/

0004-637x/753/1/18

Melli, A., Melosso, M., Tasinato, N., Bosi, G., Spada, L., Bloino, J., et al. (2018). Rotational and infrared spectroscopy of ethanimine: A route toward its astrophysical and planetary detection. *The Astrophysical Journal* 855, 123. doi:10.3847/1538-4357/aaa899

Miller, B. J., Howard, D. L., Lane, J. R., Kjaergaard, H. G., Dunn, M. E., and Vaida, V. (2009). SH-stretching vibrational spectra of ethanethiol and $tert$-butylthiol. *Journal of Physical Chemistry A* 113, 7576–7583. doi:10.1021/jp9017162

Müller, H. S. and Brünken, S. (2005). Accurate rotational spectroscopy of sulfur dioxide, SO_2, in its ground vibrational and first excited bending states, v_2 = 0, 1, up to 2 thz. *Journal of Molecular Spectroscopy* 232, 213–222. doi:10.1016/j.jms.2005.04.010

Piccardo, M., Penocchio, E., Puzzarini, C., Biczysko, M., and Barone, V. (2015). Semi-experimental equilibrium structure determinations by employing B3LYP/SNSD anharmonic force fields: Validation and application to semirigid organic molecules. *Journal of Physical Chemistry A* 119, 2058–2082. doi:10.1021/jp511432m

Pietropolli Charmet, A., Stoppa, P., Tasinato, N., Giorgianni, S., Barone, V., Biczysko, M., et al. (2013). An integrated experimental and quantum-chemical investigation on the vibrational spectra of chlorofluoromethane. *Journal of Chemical Physics* 139, 164302. doi:10.1063/1.4825380

Pietropolli Charmet, A., Stoppa, P., Tasinato, N., Giorgianni, S., and Gambi, A. (2016). Study of the vibrational spectra and absorption cross sections of 1-chloro-1-fluoroethene by a joint experimental and ab initio approach. *Journal of Physical Chemistry A* 120, 8369–8386. doi:10.1021/acs.jpca.6b07426

Puzzarini, C., Biczysko, M., Bloino, J., and Barone, V. (2014a). Accurate spectroscopic characterization of oxirane: a valuable route to its identification in titan's atmosphere and the assignment of unidentified infrared bands. *The Astrophysical Journal* 785, 107. doi:10.1088/0004-637X/785/2/107

Puzzarini, C., Senent, M. L., Domínguez-Gómez, R., Carvajal, M., Hochlaf, M., and Al-Mogren, M. M. (2014b). Accurate spectroscopic characterization of ethyl mercaptan and dimethyl sulfide isotopologues: a route toward their astrophysical detection. *Astrophysical Journal* 796, 50. doi:10.1088/0004-637X/796/1/50

Russell, D. K. and Wesendrup, R. (2003). Tunable diode laser study of the ν_3 band of oxirane. *Journal of Molecular Spectroscopy* 217, 59–71. doi:10.1016/S0022-2852(02)00010-3

Smith, D., Devlin, J. P., and Scott, D. W. (1968). Conformational analysis of ethanethiol and 2-propanethiol. *Journal of Molecular Spectroscopy* 25, 174–184. doi:10.1016/0022-2852(68)80004-9

Tasinato, N., Pietropolli Charmet, A., Stoppa, P., Giorgianni, S., and Buffa, G. (2010). Spectroscopic measurements of SO_2 line parameters in the 9.2 μm atmospheric region and theoretical determination of self-broadening coefficients. *Journal of Chemical Physics* 132, 044315. doi:10.1063/1.3299274

Tasinato, N., Pietropolli Charmet, A., Stoppa, P., Giorgianni, S., and Gambi, A. (2012a). Quantum-chemical ab initio investigation of the vibrational spectrum of halon 1113 and its anharmonic force field: A joint experimental and computational approach. *Chemical Physics* 397, 55–64. doi:10.1016/j.chemphys.2011.12.015

Tasinato, N., Regini, G., Stoppa, P., Pietropolli Charmet, A., and Gambi, A. (2012b). Anharmonic force field and vibrational dynamics of CH_2F_2 up to 5000 cm^{-1} studied by fourier transform infrared spectroscopy and state-of-the-art *ab initio* calculations. *Journal of Chemical Physics* 136, 214302. doi:10.1063/1.4720502

Wolff, H. and Szydlowski, J. (1985). Vibrational spectra and rotational isomerism of ethanethiol and ethanethiol-d_1. *Canadian Journal of Chemistry* 63, 1708–1712. doi:10.1139/v85-287
